# Leveraging chromatin accessibility for transcriptional regulatory network inference in T Helper 17 Cells

**DOI:** 10.1101/gr.238253.118

**Published:** 2019-03

**Authors:** Emily R. Miraldi, Maria Pokrovskii, Aaron Watters, Dayanne M. Castro, Nicholas De Veaux, Jason A. Hall, June-Yong Lee, Maria Ciofani, Aviv Madar, Nick Carriero, Dan R. Littman, Richard Bonneau

**Affiliations:** 1 Divisions of Immunobiology and Biomedical Informatics, Cincinnati Children's Hospital, Cincinnati, Ohio 45229, USA;; 2 Department of Pediatrics, University of Cincinnati College of Medicine, Cincinnati, Ohio 45257, USA;; 3 Molecular Pathogenesis Program, The Kimmel Center for Biology and Medicine of the Skirball Institute, New York, New York 10016, USA;; 4 Center for Computational Biology, Flatiron Institute, New York, New York 10010, USA;; 5 Department of Biology, New York University, New York, New York 10012, USA;; 6 Department of Immunology, Duke University School of Medicine, Durham, North Carolina 27710, USA;; 7 The Howard Hughes Medical Institute;; 8 Center for Data Science, New York University, New York, New York 10010, USA

## Abstract

Transcriptional regulatory networks (TRNs) provide insight into cellular behavior by describing interactions between transcription factors (TFs) and their gene targets. The assay for transposase-accessible chromatin (ATAC)–seq, coupled with TF motif analysis, provides indirect evidence of chromatin binding for hundreds of TFs genome-wide. Here, we propose methods for TRN inference in a mammalian setting, using ATAC-seq data to improve gene expression modeling. We test our methods in the context of T Helper Cell Type 17 (Th17) differentiation, generating new ATAC-seq data to complement existing Th17 genomic resources. In this resource-rich mammalian setting, our extensive benchmarking provides quantitative, genome-scale evaluation of TRN inference, combining ATAC-seq and RNA-seq data. We refine and extend our previous Th17 TRN, using our new TRN inference methods to integrate all Th17 data (gene expression, ATAC-seq, TF knockouts, and ChIP-seq). We highlight newly discovered roles for individual TFs and groups of TFs (“TF–TF modules”) in Th17 gene regulation. Given the popularity of ATAC-seq, which provides high-resolution with low sample input requirements, we anticipate that our methods will improve TRN inference in new mammalian systems, especially in vivo, for cells directly from humans and animal models.

Advances in genome-scale measurement and mathematical modeling herald opportunities for high-quality reconstruction of transcriptional regulatory networks (TRNs). TRNs describe the control of gene expression patterns by transcription factors (TFs) (Hecker et al. 2009; Chai et al. 2014), providing mechanistic (and often genome-scale) insight into the complex regulation of cellular behavior (Bonneau et al. 2007). Measurements of chromatin state represent one such advance for TRN inference. For example, chromatin immunoprecipitation with sequencing (ChIP-seq) (Robertson et al. 2007) enables identification of an individual TF's binding sites genome-wide. These data provide evidence for regulatory interactions based on proximity of the TF binding site to the gene locus and have proved valuable for TRN inference (Lee et al. 2002; Ouyang et al. 2009; Ciofani et al. 2012). However, ChIP-seq might not be feasible for cell types and physiological settings in which sample material and a priori knowledge of key transcriptional regulators are scarce.

Genome-scale chromatin accessibility measurements (Giresi et al. 2007; Xi et al. 2007; Boyle et al. 2008; Buenrostro et al. 2013) and ChIP-seq for histone marks (Barski et al. 2007) correlate with promoters, enhancers, and/or other locus control regions. These data can partially overcome limitations in a priori knowledge of cell-type–specific TF regulators if integrated with TF DNA-binding motifs (Pique-Regi et al. 2011). Large-scale efforts to characterize TF motifs are ongoing, with motifs currently available for approximately 1000 TFs in human (∼60% coverage) (Jolma et al. 2010; Weirauch et al. 2014; Lambert et al. 2018). Thus, chromatin state experiments integrated with TF motif analysis provide indirect DNA-binding evidence for hundreds of TFs. This scale would be difficult to attain from individual TF ChIP-seq experiments. Of techniques available, the assay for transposase-accessible chromatin (ATAC)–seq (Buenrostro et al. 2013) best overcomes limitations in sample abundance, requiring two orders of magnitude fewer cells than a typical ChIP-seq, FAIRE-seq, or DNase I hypersensitive sites (DHS) experiment in standard, widely adopted protocols. ATAC-seq is also possible at single-cell resolution (Buenrostro et al. 2015).

In the context of TRN inference, chromatin state measurements provide an initial set of putative TF–gene interactions based on evidence of TF binding near a gene locus. Evidence, be it direct (TF ChIP-seq) or indirect (e.g., TF motif occurrence in accessible chromatin), can be used to refine gene expression modeling (Qin et al. 2014; Blatti et al. 2015; Wilkins et al. 2016). Integration of chromatin state data in TRN inference could mitigate false-positive and false-negative TF–gene interactions expected from chromatin-state data analyzed in isolation (Äijö and Bonneau 2016; Siahpirani and Roy 2016). Sources of false positives and negatives include (1) nonfunctional binding, (2) long-range interactions between genes and regulatory regions, (3) the limited availability of individual TF ChIP experiments and incomplete knowledge of TF motifs, and (4) nonbound accessible motifs. Thus, an initial TRN derived solely from chromatin state data can be considered a useful but noisy prior, to be integrated with other data types for TRN inference.

Genome-scale inference of TRNs in mammalian settings is an outstanding challenge, given the increased complexity of regulatory mechanisms relative to simpler eukaryotes. Thus, chromatin state data are especially important for mammalian TRN inference. Construction of a genome-scale TRN for T Helper Cell Type 17 (Th17) differentiation provided a proof-of-concept for this idea (Ciofani et al. 2012). Rich genomics data sets informed the Th17 TRN: 143 RNA-seq experiments (including knockout [KO] of 20 TFs), ChIP-seq of nine TFs, and microarray from the Immunological Genome Project (Heng et al. 2008). We used the Inferelator algorithm (Bonneau et al. 2006; Madar et al. 2010) to infer TRNs from the RNA-seq and microarray and used independent methods to build networks from TF ChIP and KO data. We showed that rank combination of the networks performed best at recovering known Th17 genes and GWAS disease genes associated with Th17 pathologies.

Since the original Th17 TRN publication, the Inferelator algorithm underwent developments that improve inference in unicellular organisms and are expected to improve TRN inference in a mammalian setting (Greenfield et al. 2013; Arrieta-Ortiz et al. 2015). Although the Inferelator’s core model of transcriptional regulation still describes differential gene expression as a sparse multivariate linear function of TF activities (TFAs), the methods to solve for the TF–gene interaction terms and estimate TFAs have advanced. For example, the current Inferelator (Arrieta-Ortiz et al. 2015) uses a Bayesian approach to incorporate prior information (Greenfield et al. 2013).

The focus of this work is development of mammalian TRN inference methods from chromatin accessibility and gene expression, data types available or likely feasible for an ever-growing number of cell types, and biological conditions. In the context of mammalian TRN inference, several studies build TRNs directly from chromatin accessibility without further refinement by multivariate gene expression modeling (Neph et al. 2012; Rendeiro et al. 2016). Several other studies leverage variance in paired RNA-seq and ATAC-seq data sets; these TRN methods are exciting developments but require that ATAC-seq data for all or most RNA-seq conditions (Duren et al. 2017; Karwacz et al. 2017; Ramirez et al. 2017). In contrast, the present work is geared for TRN inference from RNA-seq and ATAC-seq, in which ATAC-seq need not exist for more than one gene expression condition.

Development of any TRN inference method requires a comprehensive benchmark with a realistic experimental design, a recurrent challenge in computational biology. We previously developed substantial genomic resources in Th17 cells (Ciofani et al. 2012), and with the addition of ATAC-seq to these resources, Th17 could be a powerful system to compare network inference from RNA-seq and ATAC-seq to a “gold standard” (GS) network constructed through the more laborious approach of TF ChIP-seq and TF KO RNA-seq. Given the central role of Th17 cells in the etiology of autoimmune and inflammatory diseases (Littman and Rudensky 2010; Stadhouders et al. 2018), an updated map of transcriptional regulation in Th17 (incorporating new experimental and computational advances) could also enhance our understanding of Th17 biology in health and disease.

## Results

### Construction of Th17 benchmark for TRN inference from ATAC-seq and RNA-seq

**Figure 1.**
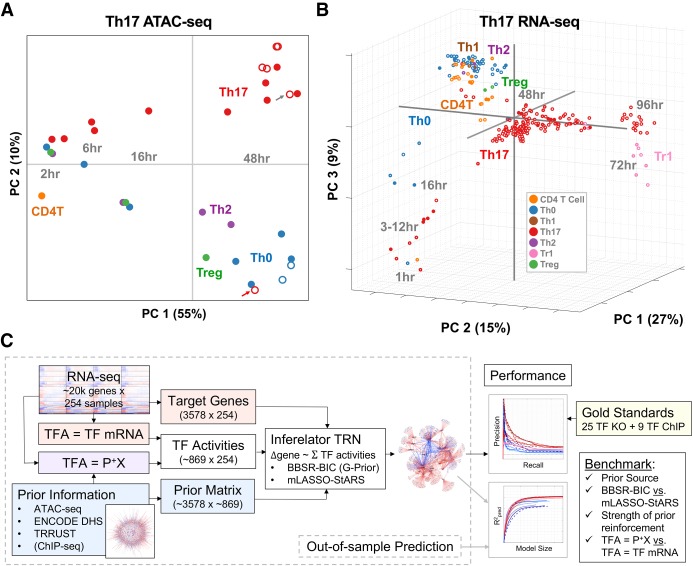
New and existing genomic benchmark resources for TRN inference in Th17. (*A*) PCA of chromatin accessibility profiles. The 33 ATAC-seq samples are plotted as a function of ATAC-seq peak intensities in PCA space, using the reference set of 63,049 ATAC-seq peaks identified. Open circles denote experimental conditions that deviate from the standard T cell differentiation conditions (e.g., gene deletion, additional cytokines). Gray and red arrows indicate *Maf* and *Stat3* KO Th17 conditions, respectively. (*B*) PCA of gene expression profiles. The 254 RNA-seq samples are plotted as a function of all genes in PCA space. (*C*) Study design (see text).

To test the feasibility of TRN inference from chromatin accessibility and gene expression alone, we generated an ATAC-seq data set in Th17 cells and other in vitro polarized T Helper (Th) cells, matching a subset of experimental conditions from the original publication (Ciofani et al. 2012) (Fig. 1A). We identified 63,049 accessible regions (peaks), and clustering revealed that most dynamically changed over the Th polarization time courses (Supplemental Fig. S1). These patterns are also apparent from principal component analysis (PCA) (Fig. 1A). Time was the most important driver of accessibility patterns. The first principal component (PC) explained 55% of the variance and captured peaks changing from two to 48 h in Th17, Th0, Th2, Treg. The second PC captured accessibility differences between Th17 and the other Th polarizations. The ATAC-seq data set contains additional perturbations, including TF KO of *Stat3* and *Maf* for Th17 and Th0 conditions (48 h). STAT3 is required for Th17 differentiation, and *Stat3* KO dramatically altered Th17 chromatin accessibility, leading to a Th0-like profile (Fig. 1A, red arrow; Supplemental Fig. S1), whereas *Maf* KO clustered with Th17 (Fig. 1A, gray arrow; Supplemental Fig. S1).

To the 143 RNA-seq experiments from the original publication, we added an additional 111 RNA-seq experiments for a total of 254 (Methods) (Fig. 1B). The majority (166 samples) were Th17, spanning 1–108 h and involving KO, siRNA knockdown, and/or drug inhibitors of TFs and signaling molecules. The study design also included other Th polarizations (Th0 [53], Tr1 [9], and Th1/Th2/Treg [two each]), as well as naive CD4^+^ T cells (25). Mirroring chromatin accessibility patterns, PCA of the gene expression data revealed time and T cell polarization conditions to be important drivers of transcriptional variation (Fig. 1B; Supplemental Fig. S2).

Although gene expression data are the only required input for the Inferelator, we hypothesized that inclusion of ATAC-seq data would improve TRN inference. Integration of ATAC-seq with TF motifs provides indirect evidence for the TF binding events driving altered chromatin state (Fig. 1A) and transcription (Fig. 1B). We generated two “prior” networks of TF–gene interactions from the ATAC-seq data: the A(Th17) prior, limited to Th17 48-h conditions, and the A(Th) prior, including all 33 Th samples (Methods). The ATAC priors contained more than 1 million putative TF–gene interactions for approximately 800 TFs (Supplemental Table S1A). To test these noisy priors in TRN inference, we developed a study design enabling quantitative performance evaluation of the resulting TRNs (Fig. 1C). Specifically, the TRNs are evaluated based on precision recall of an independent GS composed of TF–gene interactions supported by TF KO and TF ChIP data. As precision recall is limited to the TFs previously selected for KO (25 TFs) and/or ChIP-seq (nine TFs), we also evaluate TRN methods based on out-of-sample gene expression prediction.

By using precision recall and out-of-sample prediction metrics, we evaluate the effects of several key modeling decisions. Figure 1C outlines inputs to the Inferelator algorithm. From the gene expression data set, we seek to model the expression patterns of 3578 “target” genes (Methods) as functions of TFAs. Protein TFAs are rarely measured and technically infeasible for most TRN experimental designs. Thus, TFA is a hidden (or latent) variable in TRN inference (Liao et al. 2003; Fu et al. 2011). TF mRNA is the most common TFA estimate. However, many TF transcriptional activities require protein posttranslational modification. Thus, TF mRNA can be a poor proxy for protein TFA. TFA estimation based on prior knowledge of TF target genes provides an alluring alternative as it appears to be technically feasible, requiring only partial a priori knowledge of TF–gene interactions and gene expression data (Methods). “Prior-based” TFAs improved TRN inference in unicellular organisms (Arrieta-Ortiz et al. 2015; Tchourine et al. 2018). Here, we evaluate this approach in a mammalian setting.

We test two methods for model building: (1) Bayesian best subset regression with Bayesian information criteria for model selection (BBSR-BIC) (Arrieta-Ortiz et al. 2015) and (2) an alternative proposed here, modified least absolute shrinkage and selection operator (Studham et al. 2014; Gustafsson et al. 2015) with stability approach to regularization selection (Liu et al. 2010) (mLASSO-StARS). We hypothesized that mLASSO-StARS would scale better with the increased transcriptional complexity of a mammalian setting (e.g., requiring larger models; Methods). Thus, we compare mLASSO-StARS and state-of-the-art BBSR-BIC.

Prior information can enter the inference procedure at two steps: (1) to estimate prior-based TFA (described above) and (2) to reinforce prior-supported TF–gene interactions at the multivariate regression step, using BBSR-BIC or mLASSO-StARS (Fig. 1C). The strength of prior reinforcement is an important TRN inference parameter; it controls the relative contribution of the prior (e.g., TF ChIP, ATAC-seq motif analysis) to evidence from the gene expression model (variance explained by individual TFs). Thus, we test several levels of reinforcement in our study design and compare sources of prior information, in addition to ATAC-seq.

### mLASSO-StARS improves inference of a mammalian TRN

As illustrated in Figure 1C, we use precision recall to evaluate the impact of modeling decisions on TRN inference. This analysis depends on the quality of the GS. Both TF KO and TF ChIP-seq GS have caveats. Differential expression analysis of TF KOs yields an imperfect GS, as cellular TRNs adapt to the KO over time. Paralog compensation can lead to false negatives, whereas regulators downstream from the knocked-out TF can lead to secondary gene expression changes (false positives). The TF ChIP GS will also contain false positives (ChIP-seq peaks are not necessarily functional) and false negatives (peak–gene associations are based on linear proximity). Generating a GS from edges supported by both TF KO and TF ChIP reduces false positives but at the expense of false negatives. Thus, precision recall is a nuanced metric of method quality. For each GS, Supplemental Table S1A summarizes the number of edges, TFs, and target genes, as well as the percentage of overlap with other priors. Because we have KO data for 25 TFs but KO + ChIP data for only nine TFs, we also evaluate precision recall of the KO GS.

**Figure 2.**
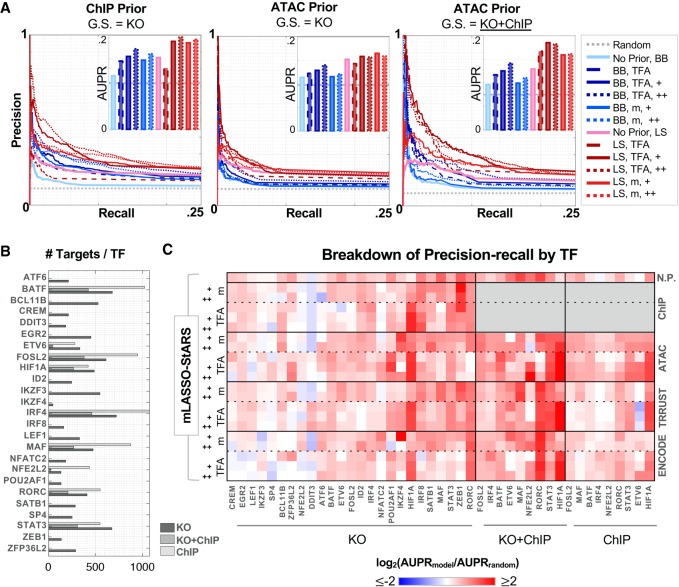
A prior network derived from ATAC-seq data improves TRN recovery of gold-standard TF–gene interactions. (*A*) Precision recall of Th17 TRNs. The *left* two panels enable comparison of TRNs built from ChIP versus Th17 ATAC priors, quantified by precision recall of the KO GS (25 TFs, 8875 interactions); *insets* display AUPR. The performance of several TRNs are plotted for each prior, based on Inferelator method. (LS) mLASSO-StARS; (BB) BBSR-BIC; (m) TF mRNA; (TFA) prior-based TFA; (+) indicates strength of prior reinforcement. Random and “no prior” TRNs serve as references. The *right* panel shows precision recall of the KO-ChIP GS (nine TFs, 2375 edges) for TRNs built from the Th17 ATAC prior. (*B*) Number of targets per TF in the GSs. Targets per TF are limited to the 3578 considered by the model. (*C*) TF-specific TRN performance. For each GS, AUPRs were calculated for each TF individually. TF-specific performance of TRNs is quantified as the log_2_ fold-change between AUPR of the TRN model relative to random. +, m, and TFA are as in *A*.

As expected, a TF ChIP-seq prior improves Th17 TRN inference (Fig. 2A, left). The ATAC prior boosts performance relative to the “no prior” control TRNs (Fig. 2A, central and right; Supplemental Fig. S3). In comparison to the ChIP-seq prior, the boost from the ATAC prior on the KO GS is smaller, likely reflecting increased levels of noise (e.g., from motif-based TF binding prediction). Also, in contrast to ChIP prior results, increasing the strength of prior reinforcement from moderate to high yields no advantage for the noisier ATAC prior. This suggests ATAC-seq prior reinforcement should be limited to moderate rather than high; gene expression data should be relied on to select a small subset of the regulatory hypothesis from the ATAC-seq prior network. For similar levels of prior reinforcement, prior-based TFA models outperform TF mRNA at low recall. For all ATAC TRNs and both GSs, mLASSO-StARS outperforms BBSR-BIC.

To explore experimental designs without context-specific chromatin accessibility, we tested two contrasting, publicly available prior information sources. The first is TF motif analysis of ENCODE DHS data from 25 mouse tissues, none of which include Th17 (Stergachis et al. 2014). The second is derived from the curated TRRUST database of human TF–gene interactions (Han et al. 2015). Although the ENCODE DHS prior includes about 1.5 million interactions between 546 TFs and about 17,000 genes (similar scale to the ATAC priors), the TRRUST prior is sparse: about 7000 interactions between 582 TFs and approximately 2000 genes (Supplemental Table S1A). The TRRUST and ENCODE priors overlap less with the GSs than context-specific priors, and this is reflected in lower precision recall relative to ChIP and ATAC priors (Supplemental Fig. S3). However, use of either the ENCODE or TRRUST prior improves performance relative to the no prior control; this improvement is substantial for prior-based TFA models. Again, across priors, TFA methods, and levels of prior reinforcement, mLASSO-StARS outperformed BBSR-BIC.

To evaluate performance on experimental designs with fewer gene expression samples, we reduced the gene expression matrix from 254 to 50 randomly selected samples (Supplemental Fig. S4). The reduced sample size had a minor impact on precision recall, especially for context-specific ChIP and ATAC priors. These results bode well for extension of our methods to contexts in which gene expression data are less abundant.

We also evaluated how the different modeling decisions affect target prediction for each TF (Fig. 2C). There is nearly an order-of-magnitude difference in TF degree in the KO + ChIP GS (Fig. 2B), so this per-TF analysis additionally ensured that results were not dominated by a few high-degree TFs. Overall, mLASSO-StARS also outperformed BBSR-BIC at TF-specific AUPR resolution (Supplemental Fig. S5).

AUPRs for many TFs were dependent on TFA estimation procedure (Fig. 2C). TF mRNA should work well for TFs whose main source of regulation is transcriptional, whereas for TFs regulated by posttranslational modification, prior-based TFA would be preferable. Consistent with this, prior-based TFA models have higher AUPR for STAT3, whereas prior-based TFA did not always improve prediction of RORC targets. With the ChIP prior (which included RORC TF ChIP), prior-based TFA AUPR was on par with TF mRNA AUPR. However, for the noisier ATAC-seq prior, prior-based TFA performed only slightly better than random, whereas TF mRNA models (including the “no prior” control) performed well across GSs. For ATAC-based TRN inference, target prediction for some TFs was better using prior-based TFA (HIF1A, STAT3, NFE2L2), whereas TF mRNA was better for some TFs (RORC, MAF, FOSL2) and roughly equivalent for others. Summarizing across priors and parameter sets, no TFA method dominates (Supplemental Fig. S5B). Based on these results, we later construct “final” Th17 TRNs using both TFA estimation methods.

### Th17 TRN models predict out-of-sample gene expression patterns

**Figure 3.**
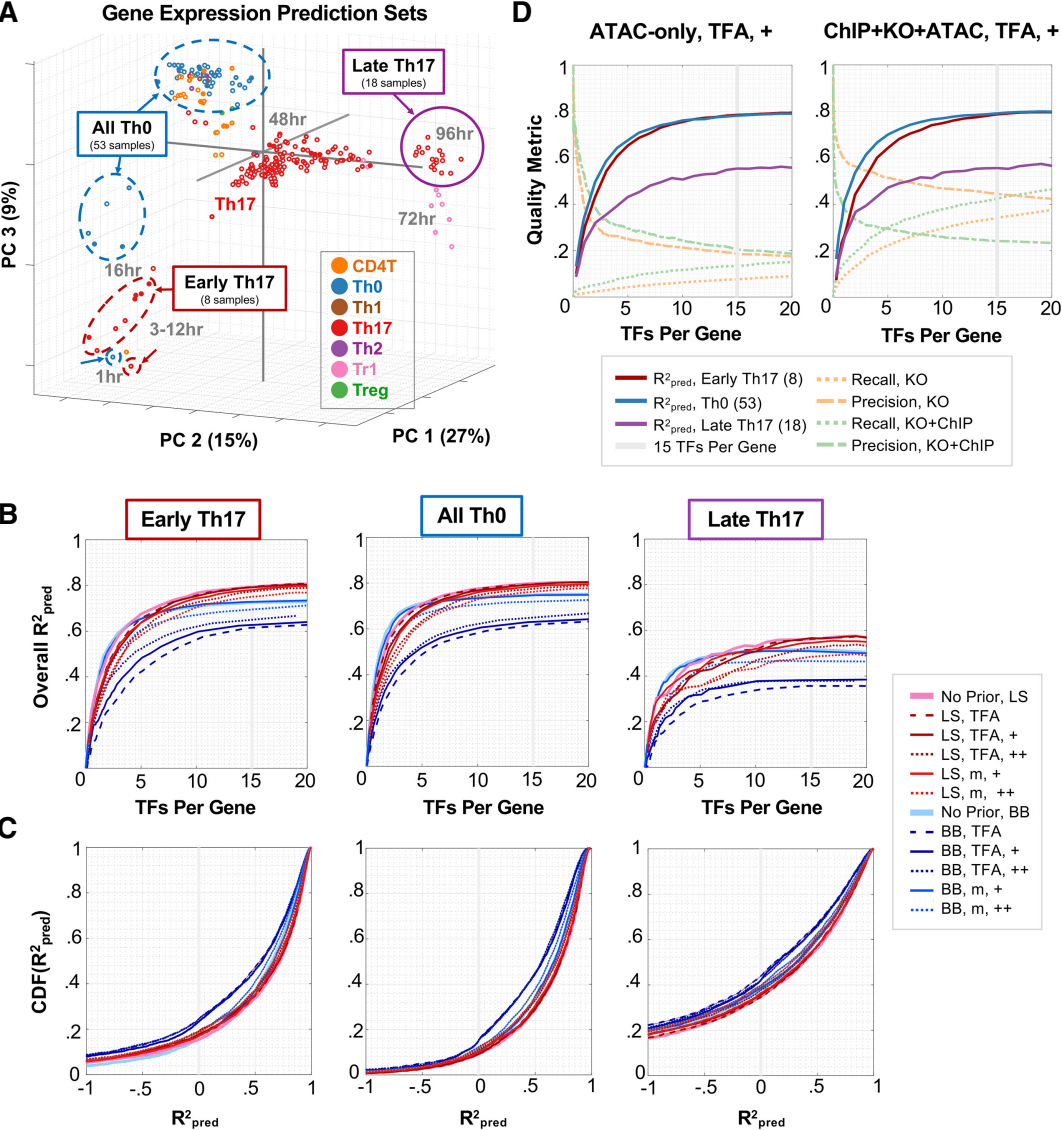
TRNs derived from RNA-seq and ATAC-seq data predict out-of-sample gene expression. (*A*) Leave-out sets plotted in PCA space. (*B*) Gene expression prediction. *R*^2^_pred_ for each leave-out set is plotted as a function of mean number of TFs per gene. (LS) mLASSO-StARS; (BB) BBSR-BIC; (m) TF mRNA; (TFA) prior-based TFA; (+) indicates strength of prior reinforcement. The gray line corresponds to a model-size cutoff of mean 15 TFs per gene. (*C*) Distributions of *R*^2^_pred_ values. Empirical cumulative distribution functions (CDFs) of per-gene *R*^2^_pred_ values for each method (model-size cutoff = mean 15 TFs per gene). (*D*) Model quality metrics versus model size. For two TRN models built with Th17 ATAC (*left*) or ChIP + KO + ATAC (*right*) priors (mLASSO-StARS, bias = 0.5, TFA = P^+^X), the quality metrics (*R*^2^_pred_ for each leave-out set, precision and recall) are plotted as a function of model size. The model size used for subsequent analyses is highlighted.

We next evaluated whether the TRN models could predict out-of-sample gene expression patterns. In contrast to precision recall, gene expression prediction provides the opportunity to evaluate all interactions in the model. This evaluation method is especially important in poorly characterized cellular contexts, for which GSs do not exist. We chose three out-of-sample prediction leave-out sets, each with distinct patterns of gene expression (highlighted in Fig. 3A). The three leave-out sets were “early Th17” (all Th17 time points between 1–16 h; eight samples), “all Th0” (Th0 samples for all time points and perturbations; 53 samples), and “late Th17” (18 Th17 samples from 60 to 108 h after TCR stimulation). For both the BBSR-BIC and mLASSO-StARS methods, we tested prediction over a range of edge confidence values and corresponding model sizes. We quantified performance using *r*-squared of prediction, $R_{\mathrm{pred}}^{2}$ (Fig. 3B,C); $R_{\mathrm{pred}}^{2}>0$ indicates that the model has predictive benefit (Methods). Across all leave-out sets and methods, out-of-sample prediction improved most as models expanded from average size of one to five TFs/gene (Fig. 3B). Most methods performed similarly well from zero to 10 TFs/gene, with the exception of BBSR-BIC models using prior-based TFA, in which prediction was worse. Predictive performance plateaued at about 10–15 TFs/gene, depending on the leave-out set (Fig. 3B, Supplemental Fig. S6). For model sizes of 10–15 TFs/genes, the mLASSO-StARS models outperformed BBSR-BIC models (Fig. 3B,C). These results, together with precision recall analyses, support mLASSO-StARS over BBSR-BIC for mammalian TRN inference.

Although we recommend StARS edge stabilities to rank interactions, we used the best quality metrics at hand (precision, recall, and $R_{\mathrm{pred}}^{2}$) to guide selection of model-size cutoff for the “final” Th17 TRN (Methods). These quality metrics are plotted versus model size (Fig. 3D; Supplemental Figs. S6, S7) for TRNs with the Th17 ATAC (“ATAC-only”) or ChIP + KO + ATAC priors. (The ChIP + KO + ATAC prior [Methods] represents our best [combined] source of prior information and is later used to derive our “final” Th17 TRN.) Once average model sizes reach approximately 15 TFs/gene (Supplemental Fig. S6), predictive performance plateaus, suggesting an average of 15 TFs/gene as a cutoff for edge inclusion in the network. Standardizing network sizes to 53,000 TF–gene interactions (about 15 TFs/gene), we calculated the percentage edge overlap among TRNs built from ATAC, ChIP, KO, ENCODE DHS, TRRUST, and combined priors. For each prior, we considered five modeling modes: prior-based TFA with no, moderate, or strong prior reinforcement and TF mRNA TFA with moderate or strong prior reinforcement. The percentage of shared edges between TRNs ranged from 83% to 10%. We clustered the networks to visualize how the modeling decisions affected resulting TRNs on a global scale (Supplemental Fig. S8; Supplemental Note 1).

### “Core” Th17 TRNs contain literature-supported TF–gene interactions

**Figure 4.**
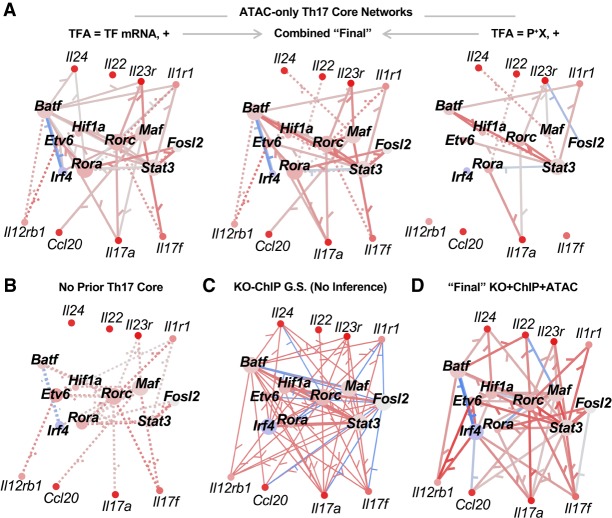
The Th17 TRNs recover key TF–gene interactions from the literature. (*A*–*D*) Th17 core TRN models. “Core” Th17 genes and TFs were selected from the literature for visual comparison with jp_gene_viz software. Network size was limited to an average of 15 TFs per gene for Inferelator networks using the following: (*A*) Th17 ATAC prior; (*B*) no prior; or (*D*) ChIP + ATAC + KO prior. The edges in Inferelator TRNs are colored according to partial correlation (red indicates positive; blue, negative) and weighted proportionally to edge stability. Solid edges have prior support, whereas dotted edges were learned from gene expression modeling alone. (*C*) The full KO-ChIP GS from Ciofani et al. (2012), where edge sign is based on differential gene expression analysis between TF KO and control. Nodes are colored according to *z*-scored gene expression at 48 h in Th17, relative to the other Th cell time points (red/blue indicates increased/decreased expression). The “final” KO + ChIP + ATAC (*D*) and ATAC-only (*B*) TRNs max-combine networks built using TF mRNA and prior-based TFA.

Our primary objective is to assess the feasibility of high-quality TRN inference from gene expression and ATAC-seq data. Therefore, it is important to examine the Th17 TRNs at high resolution. Here, we focus analysis on TRN predictions for 18 “core” Th17 TFs and genes readily familiar to Th17 biologists (Fig. 4). TF–gene interactions in this “core” have been the focus of many studies (Christie and Zhu 2014; Li et al. 2014), which we leverage to evaluate the ATAC-based Th17 core TRNs.

From the literature and the KO-ChIP data (Fig. 4C), there is support for edges between RORC and several key Th17 cytokines and receptors: *Il17a*, *Il17f*, *Il22*, *Il1r1*, and *Il23r*. Two of these interactions (*Il17a*, *Il23r*) were present in the ATAC prior and resulting TRNs (Fig. 4A). Gene expression modeling with TF mRNA was sufficient to recover four interactions (*Il17a*, *Il17f*, *Il1r1*, and *Il23r*) in the no prior TRN Figure 4B; these were also recovered in the ATAC TF mRNA TRN. The prior-based TFA ATAC TRN (Fig. 4A, right) recovers a data-driven edge between RORC and *Il22*. By combining predictions from both ATAC TRNs (Methods), all five RORC targets are recovered (Fig. 4A, central). As expected, inclusion of RORC ChIP and/or KO in the prior also leads to recovery of all five TF targets (Fig. 4D).

STAT3 is required for Th17 differentiation, playing a crucial role in driving *Rorc* expression. There is support for this interaction not only from context-specific ATAC-seq prior but also the ENCODE DHS prior. Consistent with the poor quality of mRNA-based STAT3 predictions (Fig. 2C), this interaction is not present in the no prior TRN. It is, however, recovered by all ATAC and ENCODE TRNs, even those built with TF mRNA, in which prior reinforcement likely overcomes weak correlation between TF mRNA and protein activity level (Fig. 4A–C; Supplemental Fig. S9).

MAF is another key regulator of Th17 cytokine and receptor expression, with KO-ChIP support for *Il17a*, *Il17f*, *Il23r*, and *Il1rb*. There is ATAC-seq support for MAF regulation of *Il17a*, *Il17f*, and *Il23r*. The prior-supported targets are recovered by the TF-mRNA ATAC models, but only the *Il23r* interaction is present in prior-based TFA models (Fig. 4A). Similar to *Rorc*, *Maf* mRNA might be the better proxy for TFA. Prior reinforcement also played a role, as only two of the four interactions are present in the no prior TRN (Fig. 4B). In the absence of context-specific prior information and a strong signal from the gene expression model, only a single edge (*Il23r*) was recovered by one of the ENCODE models (Supplemental Fig. S9).

These results highlight the potential for TRN inference in new settings, in which integration of chromatin accessibility and gene expression is more feasible than sequential TF ChIP and KO experiments. Consistent with the TF-resolved AUPR analysis (Fig. 2C), they also suggest that there is value to building models from both TFA methods. For construction of the “final” Th17 TRN, we combine models based on both TFA methods (Methods). The literature-curated core of our final Th17 TRN contains the RORC and MAF cytokine and receptor interactions highlighted from the literature, as well as the established connection between STAT3 and *Rorc* (Fig. 4D).

### ATAC-derived Th17 TRNs contain known and novel Th17 TFs

Having verified that the Th17 TRNs contain core Th17 TF–gene interactions from the literature, we develop a global, unbiased analysis of the final ChIP + ATAC + KO TRN to identify “core” Th17 regulators de novo. In addition, we extend our analysis to a final TRN using the ATAC-only prior to simulate mammalian TRN inference in less well studied systems, in which KO and/or ChIP data might be unavailable. Overall, prior-supported edges make up 63% and 43% of the ∼53,000 TF–gene interactions in ChIP + KO + ATAC and ATAC-only TRNs, respectively (Supplemental Table S5). Of the 715 potential TF regulators considered for final models, nearly all (∼95%) have targets in the final TRNs, with positive interactions outnumbering negative nearly twofold (1.8:1 for ChIP + KO + ATAC or 1.9:1 for ATAC-only). TF degree varies dramatically (Supplemental Figs. S10, S11). Whereas the ATAC-only network democratizes TF degree distribution (no TF has more than 500 targets), the addition of ChIP and KO leads to very high degree for several TFs in the ChIP + KO + ATAC TRN (more than 500 targets for IRF4, BATF, MAF, SP4, FOSL2, and STAT3). Although there is a bias for TFs in the prior to have higher degree, several TFs without prior support have more than 100 targets in the final networks (four TFs for the ChIP + KO + ATAC TRN and 14 TFs for the ATAC-only TRN). Thus, prior information is not weighted so strongly as to preclude inclusion of TFs without known motifs. This aspect is important for discovery and holds for TFs with edges in the prior. Although only 16% or 7% of input prior edges remain in the TRN, 27% or 46% of learned regulatory interactions for TFs with prior information are new (not originally in the prior) in KO + ChIP + ATAC or ATAC-only TRNs, respectively. Thus, our method can reduce both false negatives and false positives found in prior networks. For example, motif analysis of the RORC ChIP data revealed that only about one-third of RORC peaks contained a RORC motif (at motif occurrence cutoff *P*_raw_ = 10^−4^). Although RORC can bind DNA directly, the RORC ChIP data suggest that RORC might also bind DNA indirectly (e.g., via TF complexes). Such indirect binding would be difficult to detect by ATAC-seq motif analysis alone, but their gene targets can be recovered via gene expression modeling.

**Figure 5.**
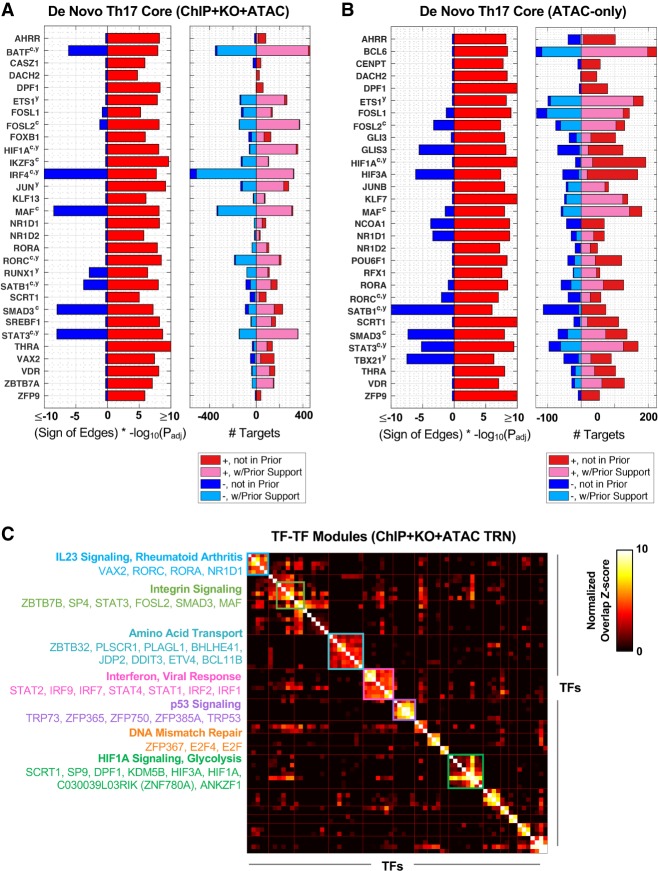
Analysis of the Th17 TRNs expands the “core” Th17 TRNs and predicts multivariate regulation of Th17 gene pathways. De novo Th17 core TFs in the ChIP + KO + ATAC TRN (*A*) and ATAC-only TRN (*B*). The Top 30 most significant “core” TFs are displayed. Significance was based on enrichment of a TF's (1) positive gene targets in up-regulated Th17 genes or (2) negative targets in down-regulated Th17 genes. (*Left*) Significance and direction of regulation; (*right*) number, sign, and prior support of TF target edges. Superscripts “c” and “y” indicate TF Th17 association from Ciofani et al. (2012) and Yosef et al. (2013), respectively. (*C*) Top 15 TF–TF modules for ChIP + KO + ATAC TRN. TFs were clustered into modules based on shared positive target genes between TFs (Methods). Gene-set enrichment was used to annotate clusters, and TF members are listed.

Many of the TFs with highest degree are shared between the ChIP + KO + ATAC and ATAC-only TRNs (e.g., IRF4, BATF, SP4, RXRA, STAT3) (Supplemental Figs. S10, S11). We developed an unbiased approach to identify key regulators of the Th17 program. TFs were included in the set of “core” Th17 regulators if they met one of two criteria: (1) The TF promotes Th17 gene expression through activation of Th17 genes, or (2) the TF promotes Th17 expression through repression of non-Th17 genes (Methods). Similar de novo Th17 core TFs were recovered from both ChIP + KO + ATAC and ATAC-only TRNs, with several recognizable Th17-specific TFs from the literature (RORA, RORC, STAT3, MAF) in both networks (Fig. 5A,B). We note that this “core” TF analysis is robust to model-size cutoffs, as analysis of TRNs with average model-size of five or 10 TFs/gene yields similar results (Supplemental Fig. S12). Similarly, top-degree TFs per TRN are robust across model sizes (Supplemental Fig. S13).

### TF–TF modules exhibit coordinated control of gene pathways in Th17

To aid in exploring the large Th17 TRNs (about 53,000 TF–gene interactions), we identified clusters of TFs with significant overlap in target genes (see Methods) (Fig. 5C; Supplemental Figs. S14, S15). We then applied a comprehensive gene-set enrichment analysis to predict functional roles for the “TF–TF clusters,” looking for consensus among pathway enrichments from five databases (Gene Ontology, Pathway Commons, KEGG, WikiPathways, and signatures from MSigDB) (Supplemental Figs. S16, S17; Kanehisa and Goto 2000; Gene Ontology Consortium 2004; Pico et al. 2008; Cerami et al. 2010; Liberzon et al. 2011). Most clusters were conserved between ChIP + KO + ATAC and ATAC-only networks, and within clusters, TFs shared features. For example, several clusters contained TFs defined in the de novo Th17 cores. RORC was a member of a Th17-promoting TF–TF module including RORA, NR1D1, and VAX2 (Fig. 5C, light-blue square; Supplemental Figs. S14–S17); functional annotations for this cluster include “IL23 signaling” and “rheumatoid arthritis,” which are consistent with prior knowledge. Th17-promoting TFs HIF1A, HIF3A, DPF1, SP9, and SCRT1 cluster with five to six other TFs (green square), and enrichments for this cluster include “hypoxia,” “HIF1A transcription factor network,” and “glycolysis.”

Other clusters contained TFs that promote the expression of genes repressed at 48 h in Th17 cells. One such cluster contained Th1 TFs (IRF1, STAT1, and STAT2) with additional interferon response factors and STATs (Fig. 5C, hot-pink box). As expected, this “interferon cluster” has enrichments for “response to interferon gamma,” “type 1 interferon pathway,” “response to virus.” Although TF gene expression for this cluster is highest in Th1 relative to other Th populations at 48 h, gene expression is at its highest at the 1-h Th17 time point, suggesting an interferon-like response for Th17 cells very early in the Th17 polarization time course (Supplemental Figs. S14, S15). This result is consistent with predictions from another Th17 TRN (Yosef et al. 2013), in which authors also predict roles for IRF1, IRF2, IRF9, STAT1, and STAT2 within the first 4 h of Th17 polarization. Both findings are consistent with potential plasticity, observed in vivo, in Th17 cell programs that are homeostatic or pathogenic, with expression of Th1-like features in the latter.

Gene-set enrichment provides functional predictions for other TF–TF modules, including “amino acid transport,” “integrin signaling,” “DNA mismatch repair,” “p53 signaling,” and others (Fig. 5C; Supplemental Figs. S14–S17). These predictions provide further confirmation of TRN quality, as many modules have predicted function in processes for which individual TFs are already implicated (e.g., HIF1A and HIF3A in the HIF1A/hypoxia module, TRP53 and TRP73 in the “p53 signaling” module). TF–TF modules and functional annotations are largely conserved between KO + ChIP + ATAC and ATAC-only TRNs, the latter prior network being much more economically feasible than the first. More fundamentally, these predictions suggest how altering sets of TFs might influence Th17 pathways and responses.

### New phenotypes are associated with TFs in the Th17 TRN

Th17 cells contribute to the pathogenesis of multiple autoimmune diseases (Stadhouders et al. 2018). We previously tested whether genes coregulated by the “Th17 core” (RORC, STAT3, BATF, IRF4, and MAF) were enriched for gene sets from GWAS of nine autoimmune diseases and three “negative controls” (Alzheimer's, schizophrenia, and type 2 diabetes) (Ciofani et al. 2012). Consistent with the known role for Th17 in autoimmune disease, genes from the autoimmune-disease sets were enriched (Ciofani et al. 2012). Since then, Th17 cells have also been implicated in obesity-related diseases (Harley et al. 2014; Endo et al. 2017) and psychiatric disorders (Debnath and Berk 2014; Choi et al. 2016). In parallel, the number of genome-wide association studies grew exponentially (MacArthur et al. 2016), and as demonstrated above, our network model improved in both comprehensiveness and accuracy.

**Figure 6.**
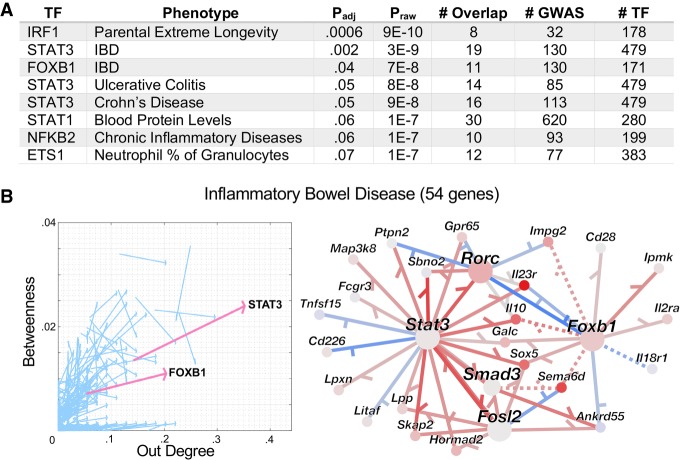
The Th17 TRNs implicate phenotypes and putative regulators in Th17 cells. (*A*) TFs whose target genes are enriched in GWAS phenotype genes (FDR = 10%). (IBD) Inflammatory bowel disease; (chronic inflammatory diseases) chronic inflammatory diseases (ankylosing spondylitis, Crohn's disease, psoriasis, primary sclerosing cholangitis, ulcerative colitis; pleiotropy). # GWAS, # TF, and # Overlap correspond to the number of genes associated with the phenotype, regulated by the TF in the Th17 TRN (KO + ChIP + ATAC), and the overlap between those two sets, respectively. Further details are contained in the Methods. (*B*) STAT3 and FOXB1 are central regulators of IBD genes. (*Left*) Each arrow corresponds to a single TF. Arrow source is TF's centrality (out degree, betweenness) in the full Th17 TRN, and arrowhead is TF centrality for the IBD subnetwork (in which target genes are limited to the 54 shared between the Th17 TRN and IBD GWAS set). STAT3 and FOXB1 (pink arrows) both show significant increase in degree centrality for IBD genes (FDR = 10%). (*Right*) The subnetwork connecting STAT3 and FOXB1 to their target genes in the IBD set. Node color indicates log_2_(fold-change) in Th17 48-h condition relative to other Th timepoints (red indicates increased; blue, decreased), whereas red/blue edges indicate positive/negative regulation. Solid edges have support in the ChIP + KO + ATAC prior, whereas dotted edges do not.

We performed an extensive, unbiased GWAS analysis of our “final” updated (KO + ChIP + ATAC) Th17 TRN, including any phenotype with five or more associated genes; 991 phenotypes met this criterion. Not only did we dramatically expand the phenotypes considered, we more broadly queried the Th17 TRN. For each of the 605 TFs individually, we tested for TF-target genes enrichment in each of the GWAS gene sets (Supplemental Table S6). Despite the large number of TF–phenotype associations tested, eight reached significance (FDR = 10%) (Fig. 6A). STAT3 targets were significantly enriched for genes associated with inflammatory bowel disease (IBD), as well as the two IBD-subtypes, Crohn's disease and ulcerative colitis. Both genetic (Cho 2008) and functional studies (Xavier and Podolsky 2007) support a role for STAT3 in IBD; indeed, STAT3 is a proposed IBD therapeutic target (Lee et al. 2015; Nguyen et al. 2015). Our analysis also newly implicates FOXB1 in regulation of IBD genes. We compared the centrality of STAT3 and FOXB1 in the Th17 TRN (Supplemental Fig. S18A) to their centrality in the subnetwork limited to the 54 IBD genes in the Th17 TRN (Fig. 6B, left). We examined both degree and betweenness centrality. For each TF, betweenness is the fraction of shortest paths connecting TFs to target genes in the network that contain the TF. Whereas degree is a local measure (TF's direct effect on gene expression), betweenness is a more global measure of TF importance, as it can also capture TFs that regulate a large number of genes through control of other TFs. Although STAT3 had the sixth-highest degree in the full Th17 TRN (Supplemental Fig. S18A), it has the highest-degree TF in the IBD subnetwork (Fig. 6B). Relative degree more than doubles for both STAT3 and FOXB1 in the IBD subnetwork, and betweenness centrality increased, too (Fig. 6B). The IBD genes regulated by STAT3 and FOXB1 include a number of Th17 genes: *Rorc*, *Il23r*, *Tnfsf15* (Fig. 6B, right).

NFKB2 and ETS1 are also associated with immune phenotypes (Fig. 6A). NFKB2's targets are enriched in the phenotype “chronic inflammatory diseases (ankylosing spondylitis, Crohn's disease, psoriasis, primary sclerosing cholangitis, ulcerative colitis) (pleiotropy)” (Supplemental Fig. S18B). Mutations in *NFKB2* have been previously associated with common variable immunodeficiency (CVID) (Chen et al. 2013; Lindsley et al. 2014; Liu et al. 2014), a heterogeneous disorder in which 25% of patients suffer autoimmune disorders, including thrombocytopenic purpura, autoimmune hemolytic anemia, rheumatoid arthritis, and autoimmune enteropathy (which can be classified as Crohn's disease) (Cunningham-Rundles 2008; Lopez-Herrera et al. 2012). Thus, *NFKB2* was previously genetically associated with pleiotropic autoimmune diseases, in the context of CVID. (We note that our set of GWAS phenotypes did not include CVID.) ETS1's targets are associated with “neutrophil percentage of granulocytes” (Supplemental Fig. S18C). ETS1 is known to repress the Th17 program (Moisan et al. 2007). *Ets1* expression decreases over the course of both Th0 and Th17 polarization and, of the 48-h Th polarization conditions, has highest expression in Treg. Mutations in *ETS1* have been associated with systemic lupus erythematosus (SLE) (Leng et al. 2011), an autoimmune disease in which the role of neutrophils has become increasingly appreciated (Smith and Kaplan 2015). Thus, a predicted role in neutrophil regulation could be consistent with the known role of ETS1 in SLE.

## Discussion

Th17 cells protect mucosa from bacteria and fungi but can also drive autoimmune and inflammatory disease (Khader et al. 2009; Littman and Rudensky 2010; Stadhouders et al. 2018). These diverse roles require coordination of thousands of genes. TF regulation of gene expression provides a map for immuno-engineering Th17 behavior in disease. Researchers in academia and industry have used our first genome-scale Th17 TRN (Ciofani et al. 2012) to develop hypotheses in the context of autoimmunity (Isono et al. 2014; Yang et al. 2014; Patel and Kuchroo 2015). Here, we provide an important update to our knowledge of Th17 transcriptional regulation, enabled by technical advances in genomic measurement and computational advances in TRN inference. KO data for 20 TFs and TF ChIP data for nine TFs were central to the original Th17 TRN, providing excellent coverage of TF–gene targets for TFs in that set. However, technical limitations and cost precluded application of these tools to the hundreds of TFs expressed over the course of Th17 differentiation, all of which could play important roles in Th17 gene expression regulation.

In combination with large-scale efforts to learn TF DNA-binding motifs (Badis et al. 2009; Jolma et al. 2013; Weirauch et al. 2014; Najafabadi et al. 2015), the advent of ATAC-seq represents an opportunity to overcome limitations of sequential TF ChIP experiments, expanding the number of TFs with chromatin binding profiles by over an order of magnitude. In addition, although TF KO and ChIP data were pragmatically limited to 48-h Th17 conditions, standard ATAC-seq protocols require two orders of magnitude fewer cells than TF ChIP. Here, we obtained (indirect) TF binding profiles from multiple differentiation time points. Yet TF binding profiles derived from motif analysis of ATAC-seq are noisy. Here, we provide a single, integrated method to infer regulatory roles for TFs genome-wide. At its core, gene expression is modeled as a function of TF activities, in which prior information (e.g., from ATAC-seq) can be used to (1) improve TF activity estimates for some TFs and (2) favor TF–gene interactions that also have prior support. We rigorously test the performance of our method in terms of precision recall and gene expression prediction. Our methods have two very desirable features (1) they prune initial noisy prior networks (by over an order of magnitude in this study) while (2) also learning new TF–gene interactions for TFs with and without prior information.

Our final Th17 TRN is built integrating our best knowledge (KO and ChIP-seq of key Th17 TFs with ATAC-seq and a rich gene expression data set). Our de novo Th17 core includes the original core (RORC, STAT3, BATF, IRF4, MAF) and dozens of additional TFs. The TF–TF module analyses predict gene pathway regulation by multiple TFs. We also exhaustively test for the association of TFs with nearly 1000 GWAS phenotypes, uncovering known associations between STAT3 and IBD, as well as several novel TF associations with immune phenotypes. Notably, these TFs were not themselves members of the gene sets for the phenotypes they are predicted to regulate. Thus, application of our TRN methods might provide new links between TF regulators and disease-associated genetic polymorphisms. The resulting Th17 TRN provides an important update to our knowledge of transcriptional regulation in Th17 cells and can be used to query key regulators of pathways and disease genes.

Of perhaps greater importance, the TRN experimental design and computational methods proposed are generalizable, designed for regimes in which prior knowledge of transcriptional regulators and/or sample material is scarce (e.g., cells directly from humans and animal models). Given the rigorous testing and case study presented here, we have high expectations for their successful application in other systems. Indeed, we have already applied our methods to a new physiological setting, constructing and experimentally validating TRNs for innate lymphoid cells of the intestine (Pokrovskii et al. 2018). Our methods are widely applicable. Prior information can be derived from diverse sources: chromatin state data, systems genetics, and literature-curated databases.

This work also highlights avenues for future improvement of TRN inference methods. We tested two methods for TF activity estimation: (1) based on TF mRNA levels and (2) based on prior knowledge of TF–gene interactions. Although prior-based TFA improved TRN inference in *Bacillus subtilis* and yeast (Arrieta-Ortiz et al. 2015; Tchourine et al. 2018), neither method consistently outperformed the other in this study. As a result, final TRNs were built using both TFA methods. There are multiple dimensions along which TFA estimation could be improved. The simplicity of the linear framework proposed for prior-based estimation has limitations in the context of complex mammalian transcriptional regulation, and a more sophisticated mathematical model for TFA estimation could be of value. TFA estimation would also improve from better prediction of TF binding events. Here, we limited our approach to a simple TF motif analysis of accessible chromatin, yet several more sophisticated methods exist and merit testing (Pique-Regi et al. 2011; Sherwood et al. 2014; Chen et al. 2017; Lamparter et al. 2017). Another limitation of our method is the mapping of putative TF binding events to gene loci. In our analysis, 3D distance between potential regulatory regions and gene loci is approximated by linear distance, a shortcoming that chromatin capture data (e.g., Hi-C [Lieberman-Aiden et al. 2009] and other 3D-chromatin techniques [Zhang et al. 2012; Beagrie et al. 2017]) would mitigate. Thus, the Th17 genomics data set (Ciofani et al. 2012), augmented by our new ATAC-seq and RNA-seq experiments, provides a fertile testing ground for the development of future TRN inference methods and innovation.

## Methods

### ATAC-seq

CD4^+^ T cells were sorted and polarized according the method previously described (Ciofani et al. 2012), and ATAC-seq samples were prepared as described previously (Buenrostro et al. 2013). Paired-end 50-bp sequences were generated from samples on an Illumina HiSeq 2500. Sequences were mapped to the murine genome (mm10) with Bowtie 2 (2.2.3) (Langmead and Salzberg 2012), filtered based on mapping score (MAPQ > 30, SAMtools [0.1.19] [Li et al. 2009]), and duplicates removed (Picard; http://broadinstitute.github.io/picard). The ATACseqQC package (Ou et al. 2018) was used to evaluate ATAC-seq fragment-length distributions and signal at TSS for each sample (Supplemental Fig. S19). For each sample individually, we ran PeaKDEck (parameters –bin 75, -STEP 25, -back 10000, -npBack100000) (McCarthy and O'Callaghan 2014) and filtered peaks with a *P*_raw_ < 10^−4^. To enable quantitative comparison of accessibility across samples, we generated a reference set of accessible regions, taking the union (BEDTools; Quinlan and Hall 2010) of peaks detected in individual samples. The reference set of ATAC-seq peaks contained 63,049 potential regulatory loci, ranging from 75 to 3725 bp (median, 275 bp). Reads per reference peak were counted with HTSeq-count (Anders et al. 2015). ATAC-seq data were robustly normalized using DESeq2 (Love et al. 2014) for PCA and clustering (Fig. 1A; Supplemental Fig. S2). The 33 ATAC-seq experiments are available from NCBI's Gene Expression Omnibus (GEO) Database (GSE113721).

### RNA-seq

The 18 new samples composing “late Th17” time points were generated as follows: Naive CD4^+^ T cells were primed on an anti-CD3 (Bio X Cell BE0001-1) and anti-CD28 (Bio X Cell BE0015-1) coated plate (without any additional cytokine) for 12–14 h (overnight). Cells were then polarized with one of two cytokine cocktails: (1) Th17N, TGF-b (0.3 ng/mL, PeproTech 100-21-10) + IL6 (20 ng/mL, eBioscience 34-8061-82); or (2) Th17P, IL6 (20 ng/mL) + IL1b (20 ng/mL, PeproTech 211-11b) + IL23 (20 ng/mL, R&D Systems 1887-ML-010). Cells were harvested for RNA-seq at 60 and 108 h after TCR stimulation. Cells were lysed and snap-frozen in TRIzol then thawed for chloroform extraction, using a 1:1 ratio of 70% ethanol to aqueous phase. Samples were loaded onto a Qiagen RNeasy column according to the manufacturer's instructions. rRNA was depleted with a Ribo-Zero gold kit; libraries were then prepared using the Illumina TruSeq stranded total RNA library prep and sequenced on an Illumina HiSeq 2500. The remaining 81 new CD4^+^ T cell samples were (1) naive or polarized and (2) processed as previously described (Ciofani et al. 2012). The 99 RNA-seq experiments are available from GEO (GSE113720). Publicly available RNA-seq data were downloaded from GEO: GSE40918 (156 samples), GSE70108 (four samples), and GSE92992 (eight samples). Sequences were mapped to mm10 (STAR aligner) (Dobin et al. 2013). Reads per gene were counted (using HTSeq-count [Anders et al. 2015] with parameters --stranded = no --mode = union) and robustly normalized (DESeq2) (Love et al. 2014). Supplemental Note 2 details treatment of batch effects.

### TRN inference

#### Selection of target genes

We built gene expression models for 3578 target genes, composed of the union of (1) genes differentially expressed between Th17 and Th0 at 48 h (FDR = 10%, log_2_|FC| > log_2_(1.5)) and (2) the 2100 genes in the original Th17 TRN (Supplemental Table S2; Ciofani et al. 2012).

#### Selection of potential regulators

We initially generated a custom list of potential mouse protein TFs, combining (1) mouse and human TFs from TFClass (Wingender et al. 2014) and genes with the GO annotation “transcription factor activity.” (Human TFs were mapped to mouse using the MGI database.) From our list of potential mouse protein TFs (2093 genes), we generated a list of 869 potential TF regulators, limited to TFs with differential gene expression in at least one pairwise comparison between Th17, Th0, Th1, Treg, or Th2 at 48 h (FDR = 10%, log_2_|FC| > log_2_(1.5)). This initial list was used for all analyses comparing mLASSO-StARS and BBSR-BIC (Figs. 2, 3B; Supplemental Figs. S3, S6). However, given recent efforts in TF annotation (Lambert et al. 2018), we generated a new list of mouse TFs for subsequent TRN analyses. Lambert et al. (2018) manually curated lists of (1) likely TFs and (2) “likely non-TFs.” We converted both lists to mouse. To gain mouse TFs without human orthologs, we integrated with mouse TFs from AnimalTFDB (Zhang et al. 2015) but removed any mouse TFs (70) that were “likely non-TFs.” Our final mouse TF list contained 1577 TFs, 715 of which served as potential regulators (differentially expressed as described above). Both candidate TF lists are available (Supplemental Table S3).

#### Generation of prior matrices

ATAC-seq peaks were associated with putative TF binding events and target genes to generate a “prior” network, *P* ∈ ℝ^|*genes*|×|*TFs*|^, of TF–gene interactions. We used a compendium of human and mouse TF motifs. Human and/or mouse TF binding motifs (PWMs) were downloaded from the Cis-BP motif collection version 1.02 (Weirauch et al. 2014; http://cisbp.ccbr.utoronto.ca) and the ENCODE motif collection (Kheradpour and Kellis 2014; http://compbio.mit.edu/encode-motifs). Transfac version 2014.2 motifs (Wingender 2008), referenced in the human Cis-BP collection, were reformatted with the MEME Suite tool transfac2meme version 4.10.1. Human ENCODE motifs were added to the Cis-BP motif collection if the TF PWM had *R*^2^ < 0.95 with a Cis-BP entry for that TF. The combined human ENCODE and Cis-BP set were mapped to mouse orthologs. We scanned peaks for individual motif occurrences with FIMO (parameters --thresh .00001, --max-stored-scores 500000, and a first-order-Markov background model) (Grant et al. 2011). We found inclusion of human TF orthologs from the ENCODE motif collection slightly increased precision recall relative to mouse Cis-BP alone (Supplemental Fig. S20). TF motif occurrences with raw *P*-value <10^−5^ were included in downstream analysis. Putative binding events were associated with a target gene, if the peak fell within ±10 kb of gene body. We tested several peak–gene association rules based on distance from gene body or TSS, and TRN inference was robust to that choice (Supplemental Fig. S21). We generated two ATAC-seq priors: (1) A(Th17), for which only peaks from Th17 48 h wild-type conditions were included, and (2) A(Th), for which all Th samples were included. For the resulting prior matrix of TF–gene interactions, entries were one if a TF motif was found proximal to the gene and zero otherwise. Similar methods were used to derive priors from ChIP-seq, TRRUST, ENCODE DHS, and combined sources (Supplemental Note 3).

#### Inference framework

We used the Inferelator model for TRN inference (Bonneau et al. 2006). At steady state (consideration of time-series is discussed in Supplemental Note 4, Supplemental Fig. S23), gene expression is modeled as a sparse, multivariate linear combination of TFAs:

(1) $$x_{ij}=\sum_{k\in TFs}b_{ik}a_{kj},$$

where *x*_*ij*_ corresponds to the expression level of gene *i* in condition *j*, *a*_*jk*_ is the activity of TF *k* in condition *j*, and *b*_*ik*_ describes the effect of TF *k* on gene *i*. A TF's mRNA expression can serve as a proxy of protein TFA. More recently (Arrieta-Ortiz et al. 2015), TFA has been estimated based on partial prior knowledge of a TF's gene targets:

(2) X=PA,

where *X* ∈ ℝ^|*genes*|×|*samples*|^ is the expression matrix for genes in the prior, *P* ∈ ℝ^|*genes*|×|*TFs*|^ is the prior matrix of known TF–gene interactions, and *A* ∈ ℝ^|*TFs*|×|*samples*|^ contains the unknown TFAs. Equation 2 has no unique solution, but the least-squares solution has worked well in simpler organisms (Arrieta-Ortiz et al. 2015; Tchourine et al. 2018). Given first-time application to a mammalian setting, we tested both methods of TFA estimation: (1) TF mRNA levels and (2) prior-based (Equation 2). Note that all expressed genes (24,007) with edges in the prior were used to solve Equation 2. As described in results, we solved for the interaction terms {*b*_*ik*_} in Equation 1 using the current Inferelator (BBSR-BIC) (Arrieta-Ortiz et al. 2015), as well as a new method (mLASSO-StARS; detailed below).

#### Model-building with LASSO and StARS

We constructed sparse models of gene expression using a modified LASSO framework:

(3) $$\hat{B}=argmin_{B}\;|X-BA{|}_{2}^{2}+|\Lambda \circ B{|}_{1},$$

where *X* and *A* are defined as above, B ∈ ℝ^|*genes*|×|*TFs*|^ is the matrix of inferred TF–gene interaction coefficients, Λ ∈ ℝ^|*genes*|×|*TFs*|^ is a matrix of nonnegative penalties, and ° represents an entry-wise matrix product (Studham et al. 2014; Gustafsson et al. 2015). Matrix representation of the LASSO penalty enables incorporation of prior information. Specifically, a smaller penalty, Λ_*ik*_, is used if there is evidence for the TF–gene interaction in the prior matrix. Similar to the G-prior in the current Inferelator BBSR and older Inferelator modified elastic net framework (Greenfield et al. 2013), this procedure encourages selection of interactions supported by the prior if there is also support in the gene expression data. For this study, the entries of the Λ matrices were limited to two values: the nonnegative value *λ*, for TF–gene interactions without evidence in the prior, and bias*λ, where bias ∈ [0,1], for TF–gene interactions with support in the prior.

We hypothesized that a data-driven approach to model selection might perform better in a complex, mammalian setting than a theoretical one (e.g., BIC used in Inferelator-BBSR-BIC). Specifically, we chose to test StARS (Liu et al. 2010), anticipating that the resulting networks would be larger than those built using our BBSR-BIC method. We hypothesized that a larger model might be needed to describe a mammalian TRN. StARS was designed to ensure that the inferred network of interactions includes the true set of network interactions with high probability. In contrast, another popular data-driven λ selection method, stability selection, seeks to limit false-positive rate (Meinshausen and Bühlmann 2010), which in a biological setting might be overly conservative (Liu et al. 2010). Thus, StARS seemed ideally suited to our objective.

In brief, StARS rests on the definition of edge instabilities. For a fixed value of *λ*, instabilities are estimated via subsampling and can be interpreted as twice the Bernoulli variance of a subsampled edge or the fraction of times subsample edge predictions disagree (Liu et al. 2010). This definition is used to select the smallest *λ* value corresponding to an acceptable average edge instability; authors heuristically recommend an average instability cutoff = 0.05.

Importantly, given our application of StARS in the new setting of TRN inference and a modified LASSO objective function, we used out-of-sample gene expression prediction and precision recall of GS interactions to guide selection of an appropriate instability cutoff rather than relying on the recommended heuristic (Supplemental Note 5; Supplemental Figs. S24–S29). Following our previous work in ecological network inference (Kurtz et al. 2015), TF–gene interactions were ranked according to nonzero subsamples per edge. To better and more efficiently prioritize high-confidence edges for TRN inference (see Supplemental Note 5; Supplemental Figs. S26–S28), we developed the following edge confidence score:

(4) Confidence(i,k)=NonzeroSubsamples+|pcorr(i,k)|,

where *i* and *k* correspond to gene *i* and TF *k*, and *pcorr*(*i*,*k*) is the partial correlation between gene *i* and TF *k*, for a set model size. For TF–gene interactions with the same number of nonzero subsamples, the TF–gene interaction with higher absolute partial correlation will be higher confidence.

mLASSO-StARS was implemented in MATLAB R2016b, and code relies on the Glmnet for MATLAB package (http://www.stanford.edu/~hastie/glmnet_matlab/) to solve Equation 3. Computational speed-ups using bStARS (Müller et al. 2016) are discussed in Supplemental Note 6 and Supplemental Fig. S30. Code is available in Supplemental Materials and from https://github.com/emiraldi/infTRN_lassoStARS.git.

#### Prior reinforcement

We tested several levels of prior reinforcement (none, moderate, and high) for BBSR-BIC and mLASSO-StARS. For BBSR-BIC, these corresponded to G-prior weights of 1, 1.1, and 1.5, for mLASSO-StARS, bias = 1, 0.5, 0.25, respectively. These prior-reinforcement parameters resulted in commensurate levels of TRN prior-edge incorporation between methods (Supplemental Fig. S22).

#### Gene expression prediction

We generated three leave-out (test) data sets (Fig. 3A; Supplemental Table S4). For each leave-out prediction challenge, the training set included all samples excluding test. For each training set, we performed model selection and parameter estimation independently of the test set. Both BBSR-BIC and the mLASSO-StARS methods provide confidence estimates for predicted TF–gene interactions, and we built TRN models of various sizes as a function of edge confidence cutoffs for each of the training sets. For parameter estimation, training TFA matrices were mean-centered and variance-normalized according to the training-set means ${\bar{a}}_{train}\in R^{\left|TFs\right|}$ and standard deviations ${\bar{\sigma }}_{train}^{\;a}\in R^{\left|TFs\right|}$. Target gene expression vectors were mean-centered according to the training-set mean ${\bar{x}}_{train}\in R^{\left|genes\right|}$. Then, for each confidence-level cutoff, we regressed the vector of normalized training gene expression data onto the reduced set of normalized training TFA estimates to arrive at a set of multivariate linear coefficients *B*_*train*_ ∈ ℝ^|*genes*|×|*TFs*|^. Sum of squared error of prediction was calculated as follows:

(5a) $$SSE_{\;pred}=\underset{\begin{array}{c} i\in |genes|\\ \;j\in \{test\} \end{array}}{\sum }{\left(x_{ij}-\underset{k\in |TFs|}{\sum }b_{ik,train}\left(\frac{a_{kj}-{\bar{a}}_{k,train}}{\sigma_{k,train}^{a}}\right)-{\bar{x}}_{i,train}\right)}^{2}.$$

The “null” model *SSE* was calculated relative to the mean of training data:

(5b) $$SSE_{null}=\underset{\begin{array}{c} i\in |genes|\\ \;j\in \{test\} \end{array}}{\sum }{\left(x_{ij}-{\bar{x}}_{i,train}\right)}^{2}.$$

We then calculated $R_{\mathrm{pred}}^{2}$, a normalized measure of predictive performance:

(5c) $$R_{pred}^{2}=1-\frac{SSE_{pred}}{SSE_{null}},\;R_{pred}^{2}\in (-\infty ,1].$$

For gene expression prediction with prior-based TFA, the mRNA of target genes with edges in the prior contribute to TFA estimation (Equation 2), and then their gene expression patterns are predicted as a function of TFA. This circularity did not lead to overfitting and inflated $R_{\mathrm{pred}}^{2}$ values (Supplemental Note 7; Supplemental Figs. S31, S32).

#### Final TRNs

To generate “final” TRNs, we used mLASSO-StARS with the following parameters: moderate prior reinforcement (bias = 0.05) and λ corresponding to average network instability = 0.05 to rank edges by confidence. To ensure that our final Th17 TRN was as complete and accurate as possible, our final network edge inclusion criteria was context-specific, guided by the most rigorous tools at hand, precision recall and out-of-sample gene expression prediction (see supporting results and discussion in Supplemental Note 5). We included the highest confidence edges until we reached a model size of average 15 TFs/gene (3578 genes × 15 TFs/gene = 53,670 TF–gene interactions). Given the complementary performance of TF-mRNA and prior-based TFA, we combined resulting TRNs by taking the maximum edge confidence to preserve the individual strengths of each (Kittler et al. 1996; Castro et al. 2019). See Supplemental Note 8 and Supplemental Figures S33 and S34 for performance comparison of max- to rank-combine (Marbach et al. 2012) relative to individual TRNs as well as performance combining TRNs from different priors.

#### De novo Th17 core

Th17 TFs were limited to TFs specifically promoting Th17 gene expression patterns. TFs were included in the core if they met one of two criteria: The TF promotes Th17 gene expression through (1) activation (the TF's positive edges are enriched in up-regulated Th17 genes at an FDR = 1%) or (2) repression of non-Th17 genes (TF's negative edges are enriched in down-regulated Th17 genes at an FDR = 1%).

#### Gold standards

For the GSs from our laboratory, we used recommended cutoffs of 0.75, 0.75, and 1.5 for KO, ChIP, and KO + ChIP networks, respectively (Ciofani et al. 2012). For the six additional TF KO experiments, we downloaded networks without filtering (Yosef et al. 2013). For both GSs, gene symbols were mapped from mm9 to mm10, and only genes mapping to both genome builds were considered in precision recall analysis. Random AUPR was calculated as the ratio of total GS edges to the number of possible edges between target genes and TFs in the GS.

### Network visualization and availability

Networks were visualized using jp_gene_viz, a newly designed interactive interface, based on iPython. Software is available at https://github.com/simonsfoundation/jp_gene_viz. All 36 LASSO-StARS Th17 TRNs (from Supplemental Fig. S8), GSs, and final, combined TRNs are available in a Jupyter-notebook binder: https://mybinder.org/v2/gh/simonsfoundation/Th17_TRN_Networks/master. Both jp_gene_viz codebase and TRN notebooks are also included in Supplemental Materials.

### TF–TF module analysis

We calculated the number of shared target genes between each pair of TFs, analyzing positive and negative target edges separately. (Edges with |partial correlation| < 0.01 were excluded from analysis, as were TFs with fewer than 20 gene targets.) TFs vary greatly by number of target genes (Supplemental Figs. S10, S11), so we devised an overlap normalization scheme that controlled for the variable number of targets per TF (Supplemental Note 9; Supplemental Fig. S35).

### GWAS analysis

The NHGRI-EBI GWAS Catalog v1.0.2 (MacArthur et al. 2016) was downloaded on August 4, 2018. SNPs were mapped to the nearest gene within ±1 Mbp using the catalog's “mapped gene(s).” Phenotype-associated gene sets were converted to mouse gene symbols. Sets containing five or more genes (991 sets) were retained. For each TF in the Th17 TRN (KO + ChIP + ATAC prior) with five or more targets (605 TFs), overlap with the GWAS gene sets was calculated and significance estimated using the hypergeometric CDF. Benjamini–Hochberg correction was applied to control for multiple hypothesis testing. Network statistics were calculated in MATLAB R2016b, normalizing degree by total target genes and fraction of shortest paths (betweenness) by total number of paths between TFs and target genes (some of which were also TFs).

## Data access

The data sets from this study have been submitted to the NCBI Gene Expression Omnibus (GEO; https://www.ncbi.nlm.nih.gov/geo/) under accession number GSE113723.

## Supplementary Material

Supplemental Material
