# Supplementary material for: Leveraging chromatin accessibility for transcriptional regulatory network inference in T Helper 17 Cells
Source: Genome Res. 2019 Mar;29(3):449–63. doi: 10.1101/gr.238253.118 (PMC6396413; doi:10.1101/gr.238253.118)
Supplement: Supplemental Material [file supp_29_3_449__index.html]

Leveraging chromatin accessibility for transcriptional regulatory network inference in T Helper 17 Cells — Supplemental Material 

# Leveraging chromatin accessibility for transcriptional regulatory network inference in T Helper 17 Cells

## Supplemental Material

- Supplemental\_Fig\_S1.pdf
- Supplemental\_Fig\_S2.pdf
- Supplemental\_Fig\_S3.pdf
- Supplemental\_Fig\_S4.pdf
- Supplemental\_Fig\_S5.pdf
- Supplemental\_Fig\_S6.pdf
- Supplemental\_Fig\_S7.pdf
- Supplemental\_Fig\_S8.pdf
- Supplemental\_Fig\_S9.pdf
- Supplemental\_Fig\_S10.pdf
- Supplemental\_Fig\_S11.pdf
- Supplemental\_Fig\_S12.pdf
- Supplemental\_Fig\_S13.pdf
- Supplemental\_Fig\_S14.pdf
- Supplemental\_Fig\_S15.pdf
- Supplemental\_Fig\_S16.pdf
- Supplemental\_Fig\_S17.pdf
- Supplemental\_Fig\_S18.pdf
- Supplemental\_Fig\_S19.pdf
- Supplemental\_Fig\_S20.pdf
- Supplemental\_Fig\_S21.pdf
- Supplemental\_Fig\_S22.pdf
- Supplemental\_Fig\_S23.pdf
- Supplemental\_Fig\_S24.pdf
- Supplemental\_Fig\_S25.pdf
- Supplemental\_Fig\_S26.pdf
- Supplemental\_Fig\_S27.pdf
- Supplemental\_Fig\_S28.pdf
- Supplemental\_Fig\_S29.pdf
- Supplemental\_Fig\_S30.pdf
- Supplemental\_Fig\_S31.pdf
- Supplemental\_Fig\_S32.pdf
- Supplemental\_Fig\_S33.pdf
- Supplemental\_Fig\_S34.pdf
- Supplemental\_Fig\_S35.pdf
- Supplemental\_jp\_gene\_viz.zip
- Supplemental\_Materials.docx
- Supplemental\_Table\_S1.pdf
- Supplemental\_Table\_S2.xlsx
- Supplemental\_Table\_S3.xlsx
- Supplemental\_Table\_S4.xlsx
- Supplemental\_Table\_S5.xlsx
- Supplemental\_Table\_S6.xlsx
- Supplemental\_infTRN\_lassoStARS.zip
- Supplemental\_Th17\_TRN\_Networks.zip
