# Supplementary material for: Leveraging chromatin accessibility for transcriptional regulatory network inference in T Helper 17 Cells
Source: Genome Res. 2019 Mar;29(3):449–63. doi: 10.1101/gr.238253.118 (PMC6396413; doi:10.1101/gr.238253.118)
Supplement: Supplemental Material [file supp_gr.238253.118_Supplemental_Fig_S1.pdf]

Figure S1

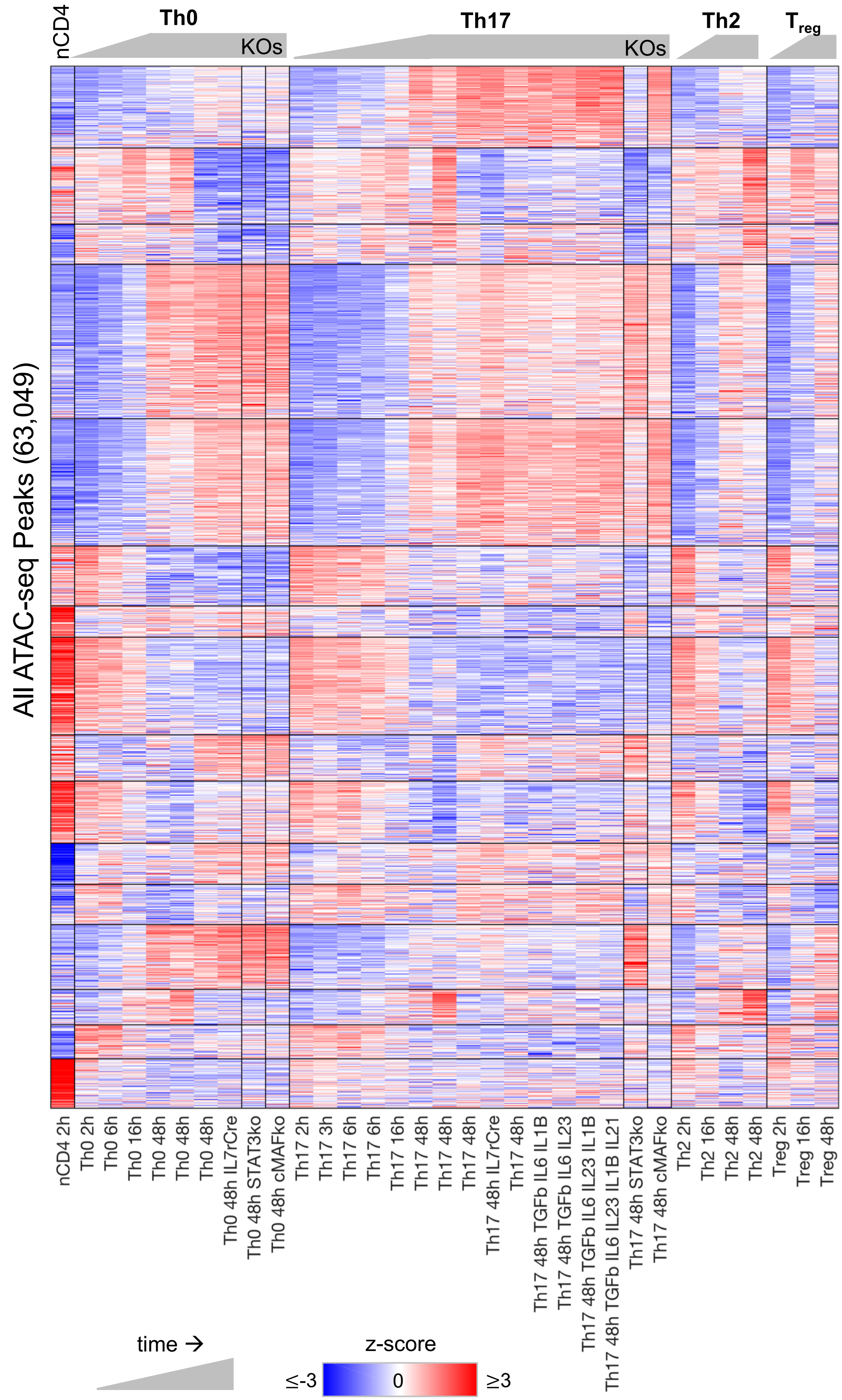

Figure S1. Clustering of all 63,049 peaks in the ATAC-seq dataset. ATAC-seq peak intensities were robustly normalized (DESeq2), z-scored and clustered using K-means clustering with Euclidean distance. Samples were ordered according to treatment condition and timepoint.
