# Supplementary material for: Leveraging chromatin accessibility for transcriptional regulatory network inference in T Helper 17 Cells
Source: Genome Res. 2019 Mar;29(3):449–63. doi: 10.1101/gr.238253.118 (PMC6396413; doi:10.1101/gr.238253.118)
Supplement: Supplemental Material [file supp_gr.238253.118_Supplemental_Fig_S2.pdf]

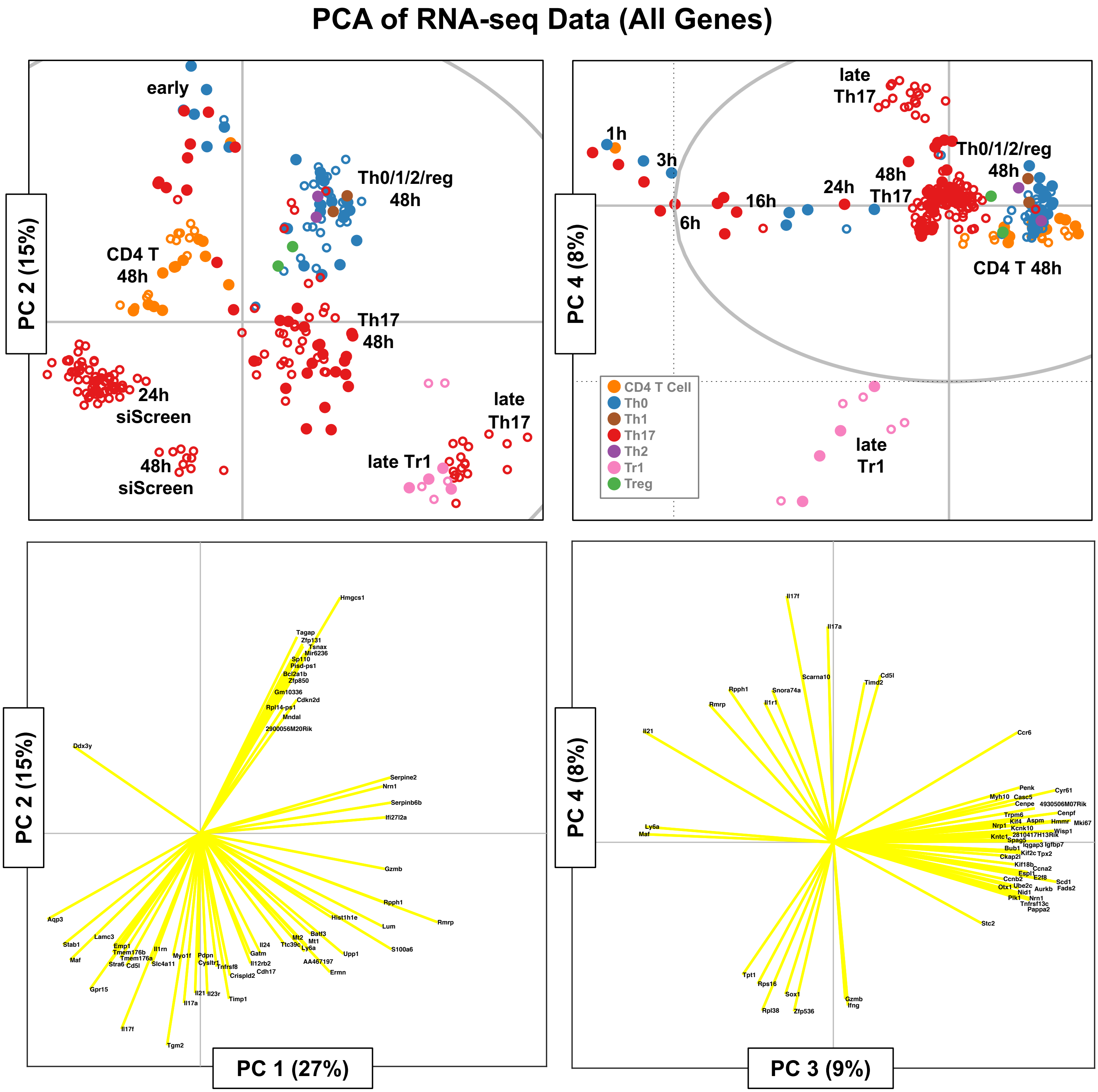

Figure S2. PCA of Gene Expression Profiles. Upper panels show scores plots, where the 254 RNA-seq samples are plotted as a function of all genes for principal components (PCs) 1-4. Lower panels display gene loadings for the top-75 gene contributors to PCs 1-4.
