# Supplementary material for: Leveraging chromatin accessibility for transcriptional regulatory network inference in T Helper 17 Cells
Source: Genome Res. 2019 Mar;29(3):449–63. doi: 10.1101/gr.238253.118 (PMC6396413; doi:10.1101/gr.238253.118)
Supplement: Supplemental Material [file supp_gr.238253.118_Supplemental_Fig_S4.pdf]

Figure S4

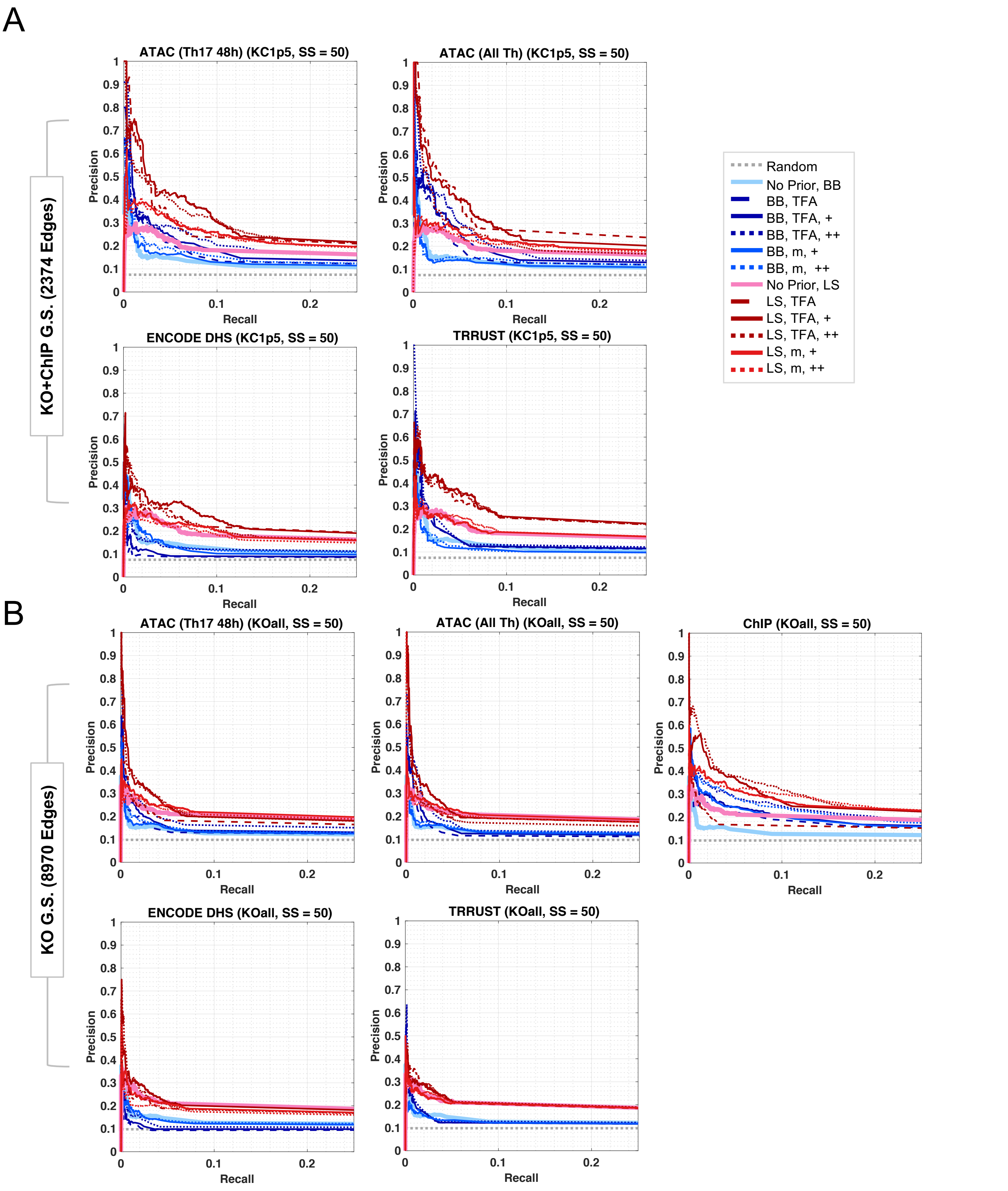

Figure S4. Precision-recall of multiple priors, G.S. = KO+ChIP (A) or KO (B), effect of RNA-seq dataset size. TRNs were built from 50 RNA-seq samples (rather than the full 254). For each prior, the performance is plotted for several TRNs, based on *Inferelator* method (LS = mLASSO-StARS (reds), BB = BBSR-BIC (blues)), TFA estimation method (m = TF mRNA, TFA = $P^{+}X$), and strength of prior reinforcement (none, moderate (+), and strong (++)). Random and "No Prior" control TRNs serve as references in all panels.
