# Supplementary material for: Leveraging chromatin accessibility for transcriptional regulatory network inference in T Helper 17 Cells
Source: Genome Res. 2019 Mar;29(3):449–63. doi: 10.1101/gr.238253.118 (PMC6396413; doi:10.1101/gr.238253.118)
Supplement: Supplemental Material [file supp_gr.238253.118_Supplemental_Fig_S5.pdf]

Figure S5

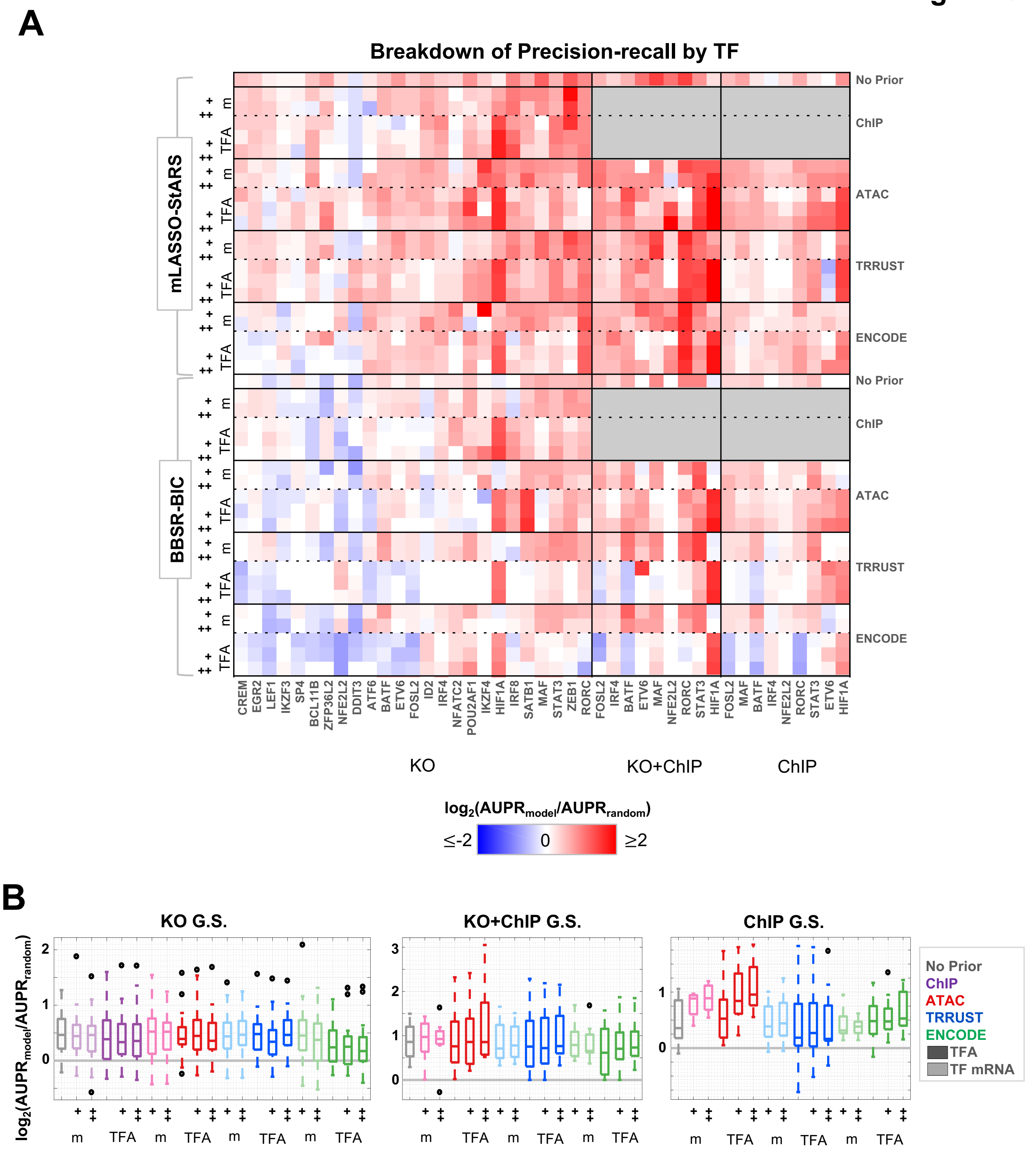

Figure S5. TF-specific TRN performance for mLASSO-StARS and BBSR-BIC. (A) For each G.S., AUPRs were calculated for each TF individually. TF-specific performance of TRNs is quantified as the $\log_2$-foldchange between AUPR of the TRN model relative to random. "+" indicates strength of prior reinforcement, and "m" and "TFA" denote TFA estimation from TF mRNA and prior-based TFA, respectively. **(B)** Boxplots of per-TF AUPRs for mLASSO-StARS models in **(A);** central mark indicates median, box edges indicate the 25th and 75th percentiles, and the whiskers extend to the most extreme data points not considered outliers (+/-2.7 standard deviations from the mean).
