# Supplementary material for: Leveraging chromatin accessibility for transcriptional regulatory network inference in T Helper 17 Cells
Source: Genome Res. 2019 Mar;29(3):449–63. doi: 10.1101/gr.238253.118 (PMC6396413; doi:10.1101/gr.238253.118)
Supplement: Supplemental Material [file supp_gr.238253.118_Supplemental_Fig_S6.pdf]

Figure S6

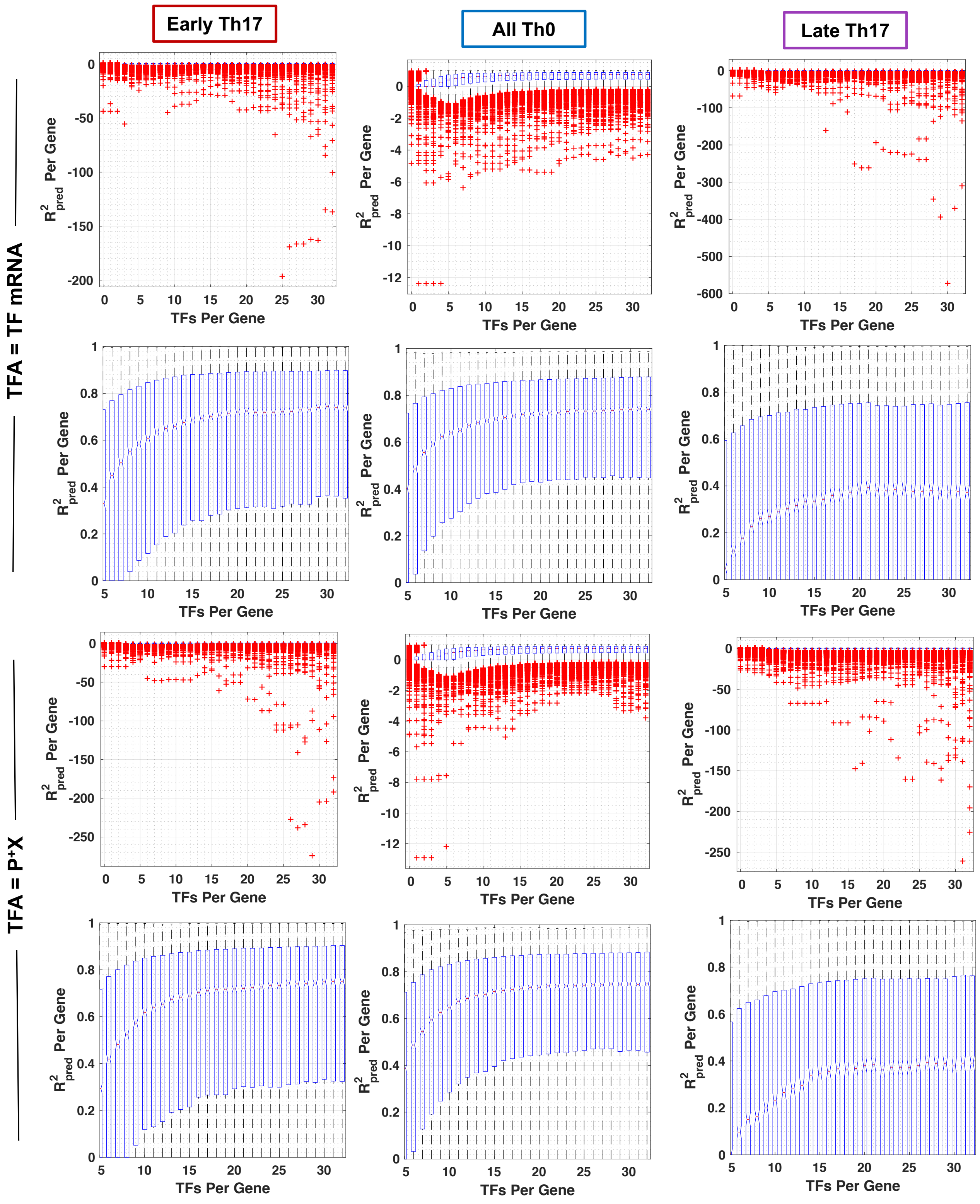

Figure S6. Distributions of per-gene R^2_{pred} values. Full and zoomed-in boxplots of per-gene R^2_{pred} values for the Th17 ATAC prior, bias = .5 and TFA = TF mRNA or prior-based TFA (TFA = P^+X). The central red mark indicates median, the bottom and top edges of the blue box indicate the 25th and 75th percentiles, and the whiskers extend to the most extreme data points not considered outliers (+/- 2.7 standard deviations from the mean). x-axis indicates the average number of TFs per gene at each model-size cutoff.
