# Supplementary material for: Leveraging chromatin accessibility for transcriptional regulatory network inference in T Helper 17 Cells
Source: Genome Res. 2019 Mar;29(3):449–63. doi: 10.1101/gr.238253.118 (PMC6396413; doi:10.1101/gr.238253.118)
Supplement: Supplemental Material [file supp_gr.238253.118_Supplemental_Fig_S7.pdf]

Figure S7

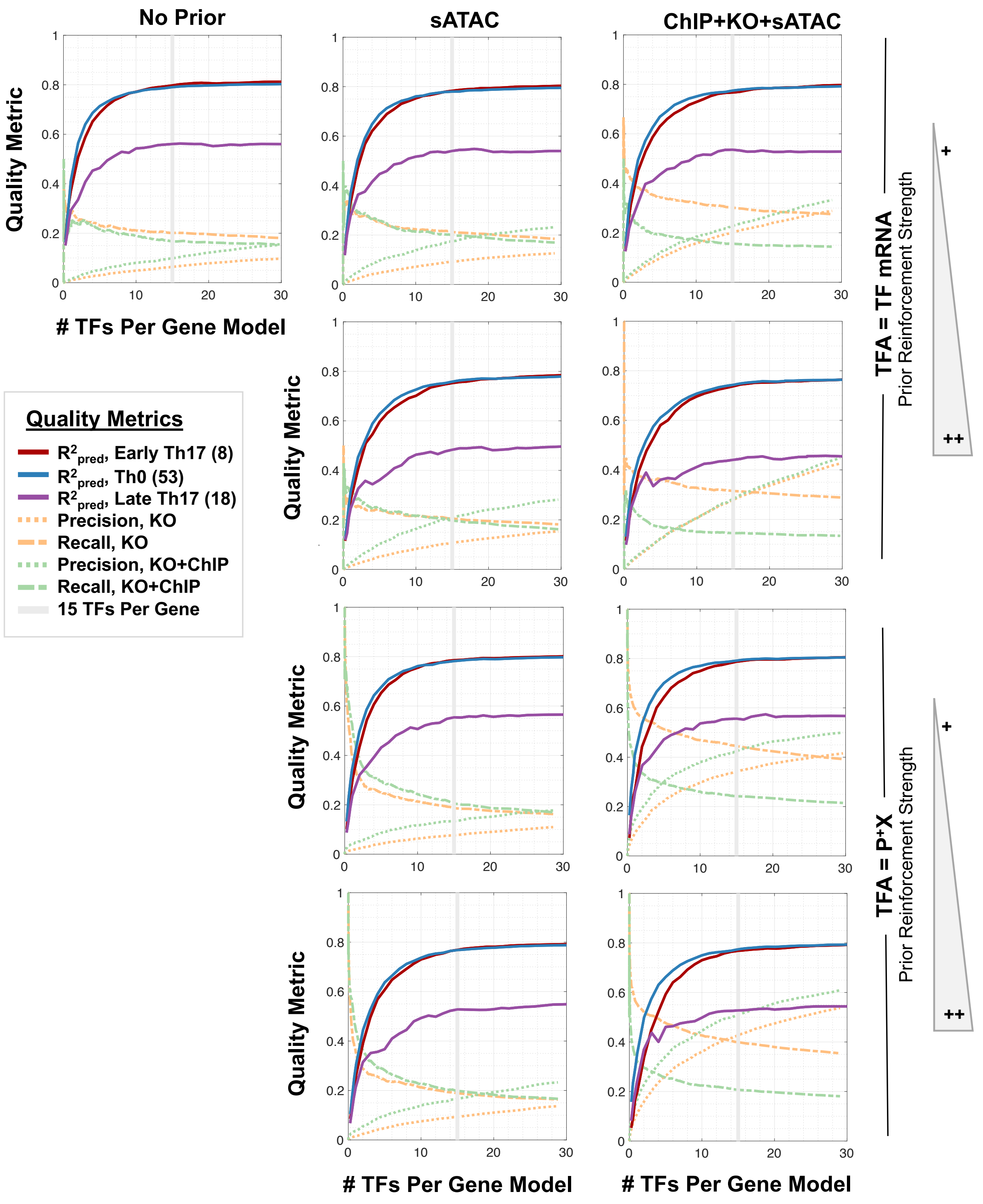

Figure S7. Out-of-sample prediction of gene expression patterns extended to include TFA = TF mRNA and No Prior TRNs. Leave-out sets are defined as in **Figure 4A**. Model selection and parameter estimation of TF-gene interactions were performed in the absence of specified leave-out sets (All Th0, Late Th17, and Early Th17). TRN models were built without prior (left panels), with an ATAC (central panels) or ChIP+KO+ATAC priors (right panels), using mLASSO-StARS, bias = .5, and TFA = TF mRNA (upper panels) TFA = $P^{+}X$ (lower panels). R^2_{pred} for each leave-out set is plotted as a function of average number of TFs per gene. In the key, the number of samples per leave-out set appears in parentheses. For reference, precision and recall of the full model are also plotted, providing intuition about the number of KO or KO+ChIP edges in the TRN at each cut off.
