# Supplementary material for: Leveraging chromatin accessibility for transcriptional regulatory network inference in T Helper 17 Cells
Source: Genome Res. 2019 Mar;29(3):449–63. doi: 10.1101/gr.238253.118 (PMC6396413; doi:10.1101/gr.238253.118)
Supplement: Supplemental Material [file supp_gr.238253.118_Supplemental_Fig_S8.pdf]

Figure S8

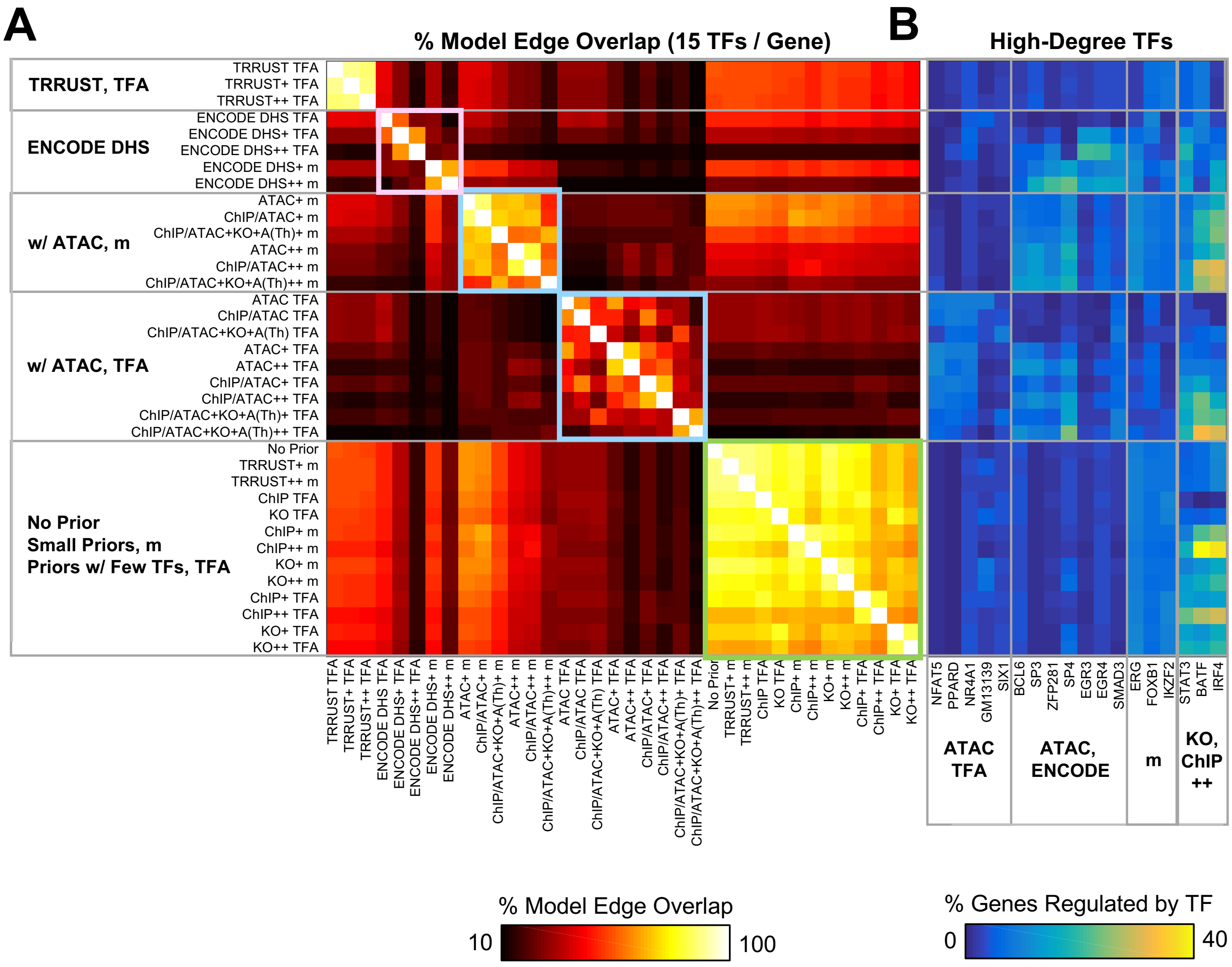

Figure S8. (A) Edge overlaps of TRNs. TRNs were built with the Th17 ATAC, ChIP, KO, ENCODE DHS, TRRUST, ChIP/ATAC or ChIP+ATAC+KO priors, with TFA based on target genes ("TFA") or TF mRNA ("m") and varying strengths of prior reinforcement (+ = moderate, ++ = strong, or none). The "No Prior" TRN was included as a control. Each TRN model size was limited to a mean of 15 TFs per gene and hierarchically clustered using 1-overlap distance and ward linkage. **(B) Network coverage by high-degree TFs.** A TF was included in the "high-degree" TF set, if it was within the top-two highest degree nodes in one or more of the TRNs. The percentage of genes regulated by TF is based on the 3578 target genes.
