# Supplementary material for: Leveraging chromatin accessibility for transcriptional regulatory network inference in T Helper 17 Cells
Source: Genome Res. 2019 Mar;29(3):449–63. doi: 10.1101/gr.238253.118 (PMC6396413; doi:10.1101/gr.238253.118)
Supplement: Supplemental Material [file supp_gr.238253.118_Supplemental_Fig_S9.pdf]

Figure S9

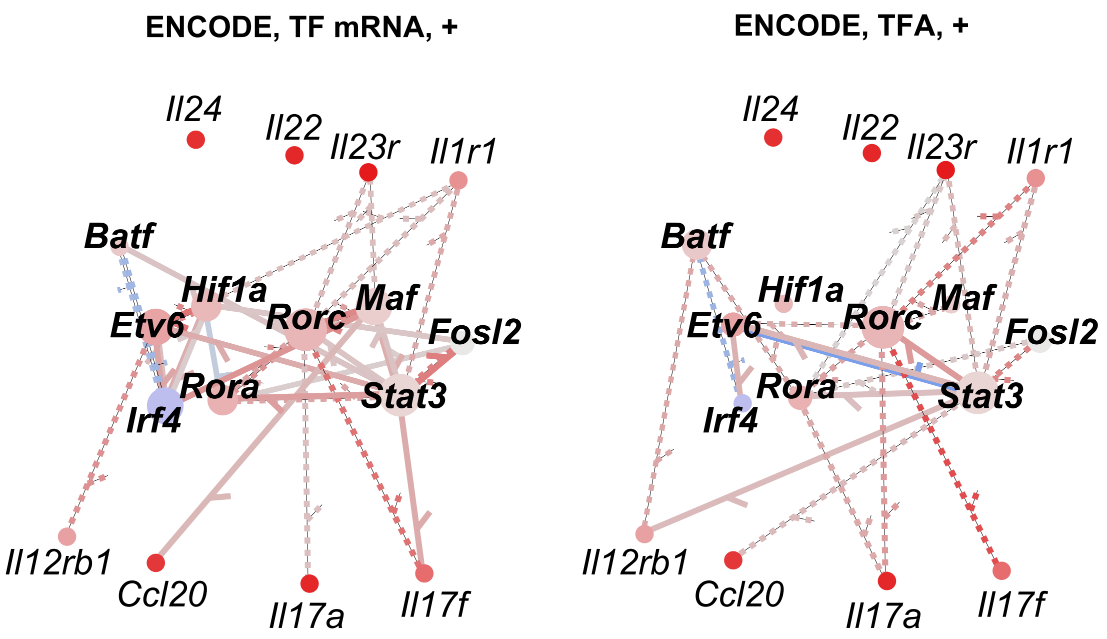

Figure S9. Additional Th17 core TRN models. "Core" Th17 genes and TFs were selected from the literature for visual comparison with jp_gene_viz software. Network size was limited to an average of 15 TFs per gene. Source of network and TFA method is denoted for each network. "+" denote moderate prior reinforcement with bias = .5. The edges in Inferelator TRNs are colored according to partial correlation (red positive, blue negative) and weighted relative to edge stability. Solid edges had support in the prior, while dotted edges were learned from gene expression modeling alone. Nodes are colored according to z-scored gene expression at 48h in Th17 relative to the other T Helper cell time points (red/blue = increased/decreased expression).
