# Supplementary material for: Leveraging chromatin accessibility for transcriptional regulatory network inference in T Helper 17 Cells
Source: Genome Res. 2019 Mar;29(3):449–63. doi: 10.1101/gr.238253.118 (PMC6396413; doi:10.1101/gr.238253.118)
Supplement: Supplemental Material [file supp_gr.238253.118_Supplemental_Fig_S10.pdf]

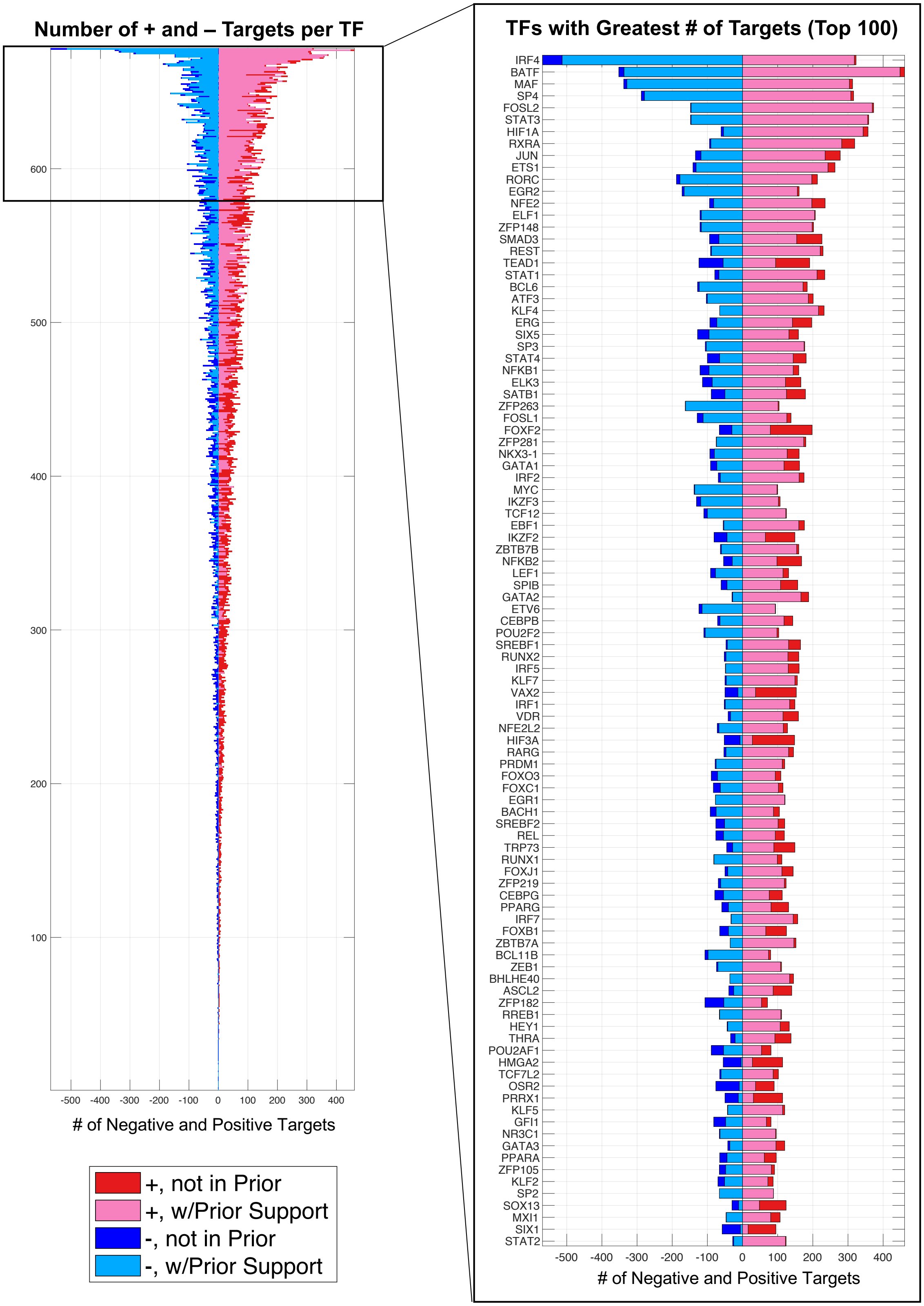

Figure S12. Distribution of targets per TF in final ChIP+KO+ATAC TRN. Network size was limited to mean 15 TFs per gene and network edges were further filter to remove any edge with absolute partial correlation < .01. TFs were ranked according to degree (total number of target genes) in the left panel, while the inset displays the Top 100 highest-degree TFs. TF target genes are colored according to interaction sign and whether the interaction was also in the prior (see key).
