# Supplementary material for: Leveraging chromatin accessibility for transcriptional regulatory network inference in T Helper 17 Cells
Source: Genome Res. 2019 Mar;29(3):449–63. doi: 10.1101/gr.238253.118 (PMC6396413; doi:10.1101/gr.238253.118)
Supplement: Supplemental Material [file supp_gr.238253.118_Supplemental_Fig_S11.pdf]

Figure S11

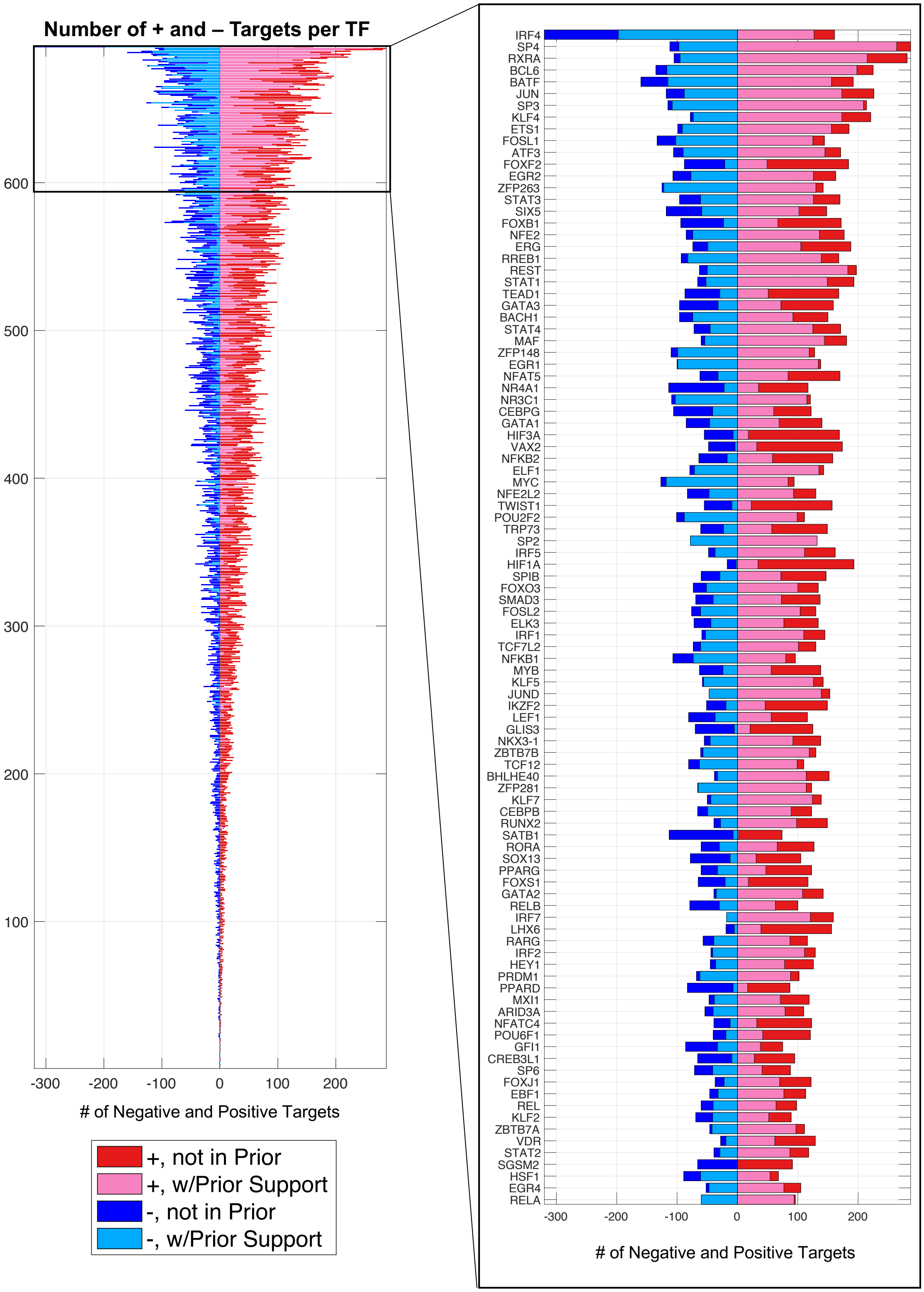

Figure S13. Distribution of targets per TF in final ATAC TRN. Network size was limited to mean 15 TFs per gene and network edges were further filter to remove any edge with absolute partial correlation < .01. TFs were ranked according to degree (total number of target genes) in the left panel, while the inset displays the Top 100 highest-degree TFs. TF target genes are colored according to interaction sign and whether the interaction was also in the prior (see key).
