# Supplementary material for: Leveraging chromatin accessibility for transcriptional regulatory network inference in T Helper 17 Cells
Source: Genome Res. 2019 Mar;29(3):449–63. doi: 10.1101/gr.238253.118 (PMC6396413; doi:10.1101/gr.238253.118)
Supplement: Supplemental Material [file supp_gr.238253.118_Supplemental_Fig_S12.pdf]

Figure S12

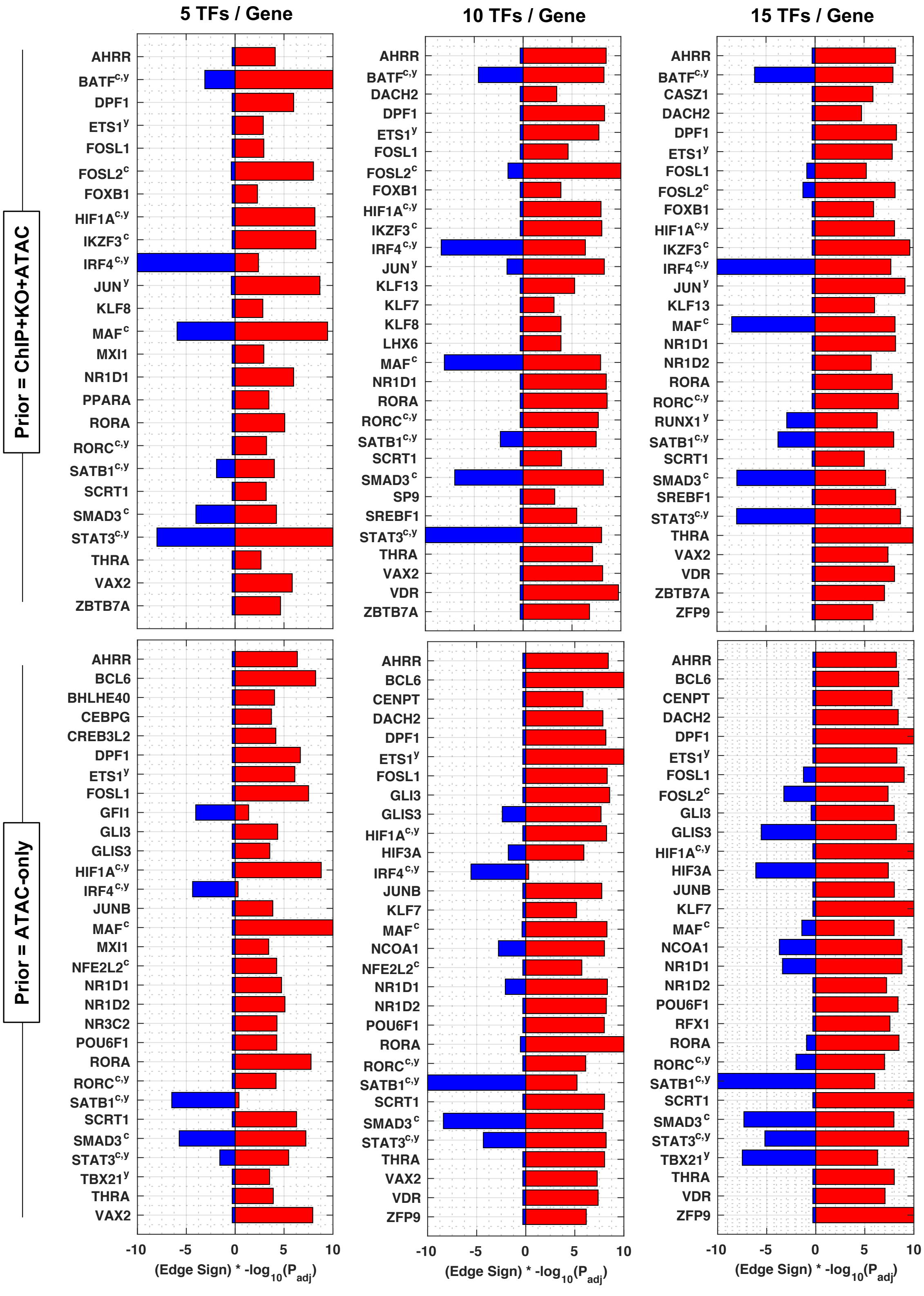

Figure S12. De Novo Th17 Core TFs of the ChIP+KO+ATAC and ATAC-only TRNs derived from different model-size cutoffs. TFs were included as De Novo Th17 Core TFs if, at FDR = 10%, (1) positive gene targets were enriched for up-regulated Th17 genes (red bars) or (2) negative gene targets were enriched for down-regulated Th17 genes (blue bars). The top-30 most significant "core" Th17 TFs are shown per TRN. Superscripts c and y indicate TF Th17 association from (Ciofani et al. 2012), (Yosef et al., 2013), respectively.
