# Supplementary material for: Leveraging chromatin accessibility for transcriptional regulatory network inference in T Helper 17 Cells
Source: Genome Res. 2019 Mar;29(3):449–63. doi: 10.1101/gr.238253.118 (PMC6396413; doi:10.1101/gr.238253.118)
Supplement: Supplemental Material [file supp_gr.238253.118_Supplemental_Fig_S13.pdf]

Figure S13

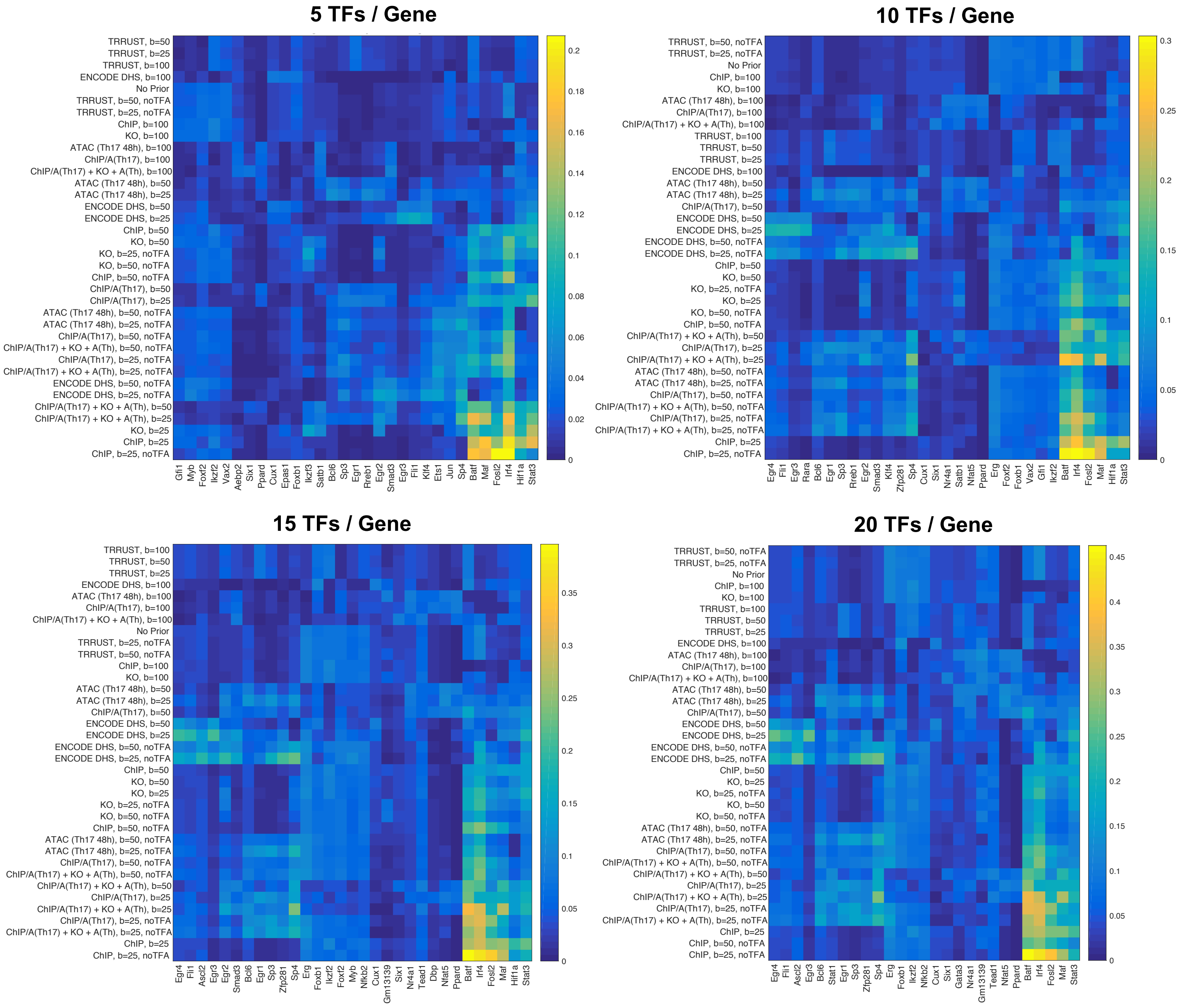

Figure S13. Effect of model-size cutoff on high-degree TFs. A TF was included in the "high-degree" TF set, if it was within the top-three highest-degree TFs in one or more of the TRNs. Color scale indicates the percentage of (the 3758) target genes regulated by the TF.
