# Supplementary material for: Leveraging chromatin accessibility for transcriptional regulatory network inference in T Helper 17 Cells
Source: Genome Res. 2019 Mar;29(3):449–63. doi: 10.1101/gr.238253.118 (PMC6396413; doi:10.1101/gr.238253.118)
Supplement: Supplemental Material [file supp_gr.238253.118_Supplemental_Fig_S14.pdf]

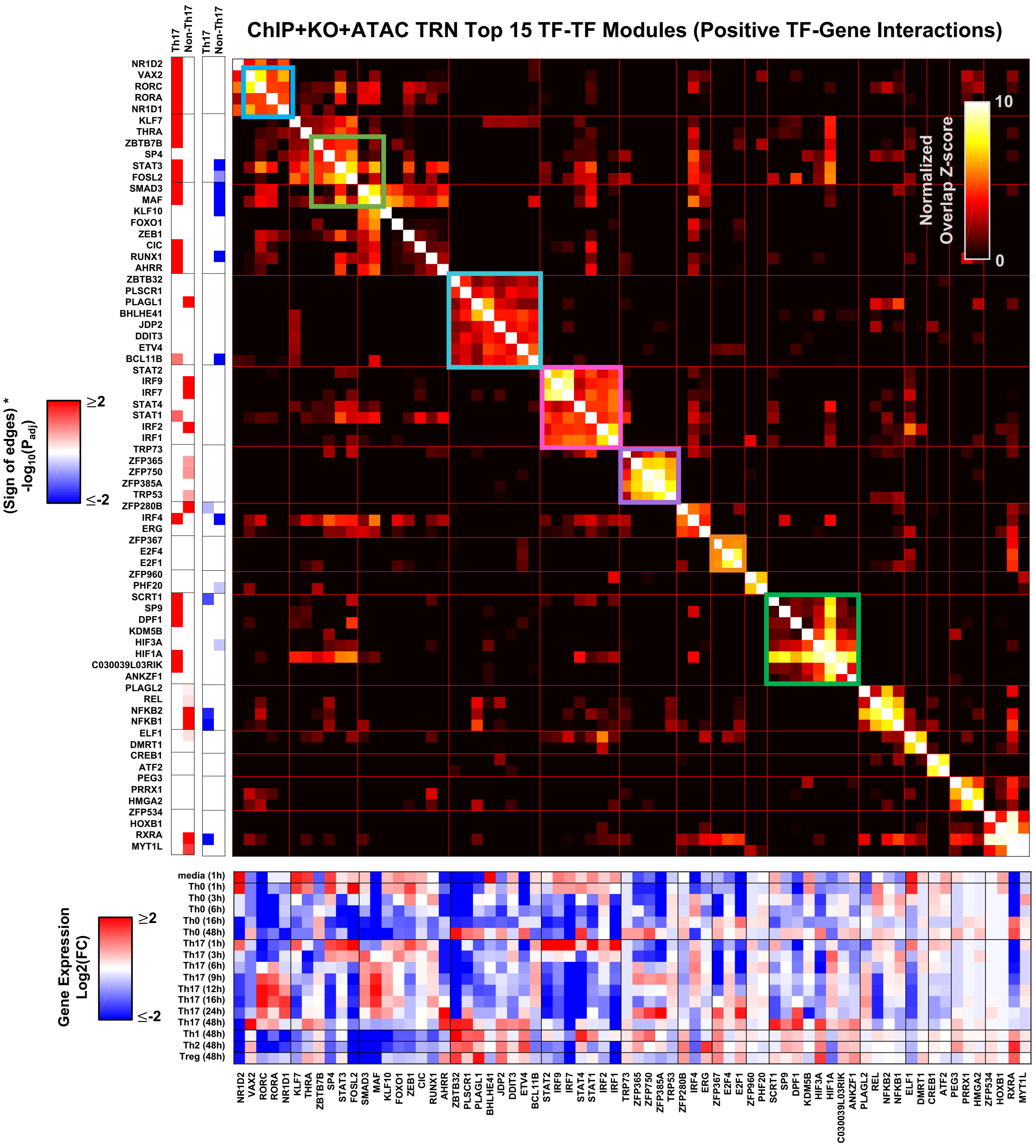

Figure S14. Top 15 TF modules for ChIP+KO+ATAC TRN. TFs were clustered into modules based on shared positive target genes between TFs (see **Methods**). The left panel shows enrichment of positive TF target interactions with up-regulated Th17 genes ("Th17" for potential Th17-promoting TFs) or down-regulated Th17 genes ("Non-Th17", for potential Th17-repressing TFs). The lower panel shows relative TF gene expression over the Th17 and other T Helper cell time points.
