# Supplementary material for: Leveraging chromatin accessibility for transcriptional regulatory network inference in T Helper 17 Cells
Source: Genome Res. 2019 Mar;29(3):449–63. doi: 10.1101/gr.238253.118 (PMC6396413; doi:10.1101/gr.238253.118)
Supplement: Supplemental Material [file supp_gr.238253.118_Supplemental_Fig_S16.pdf]

Figure S16

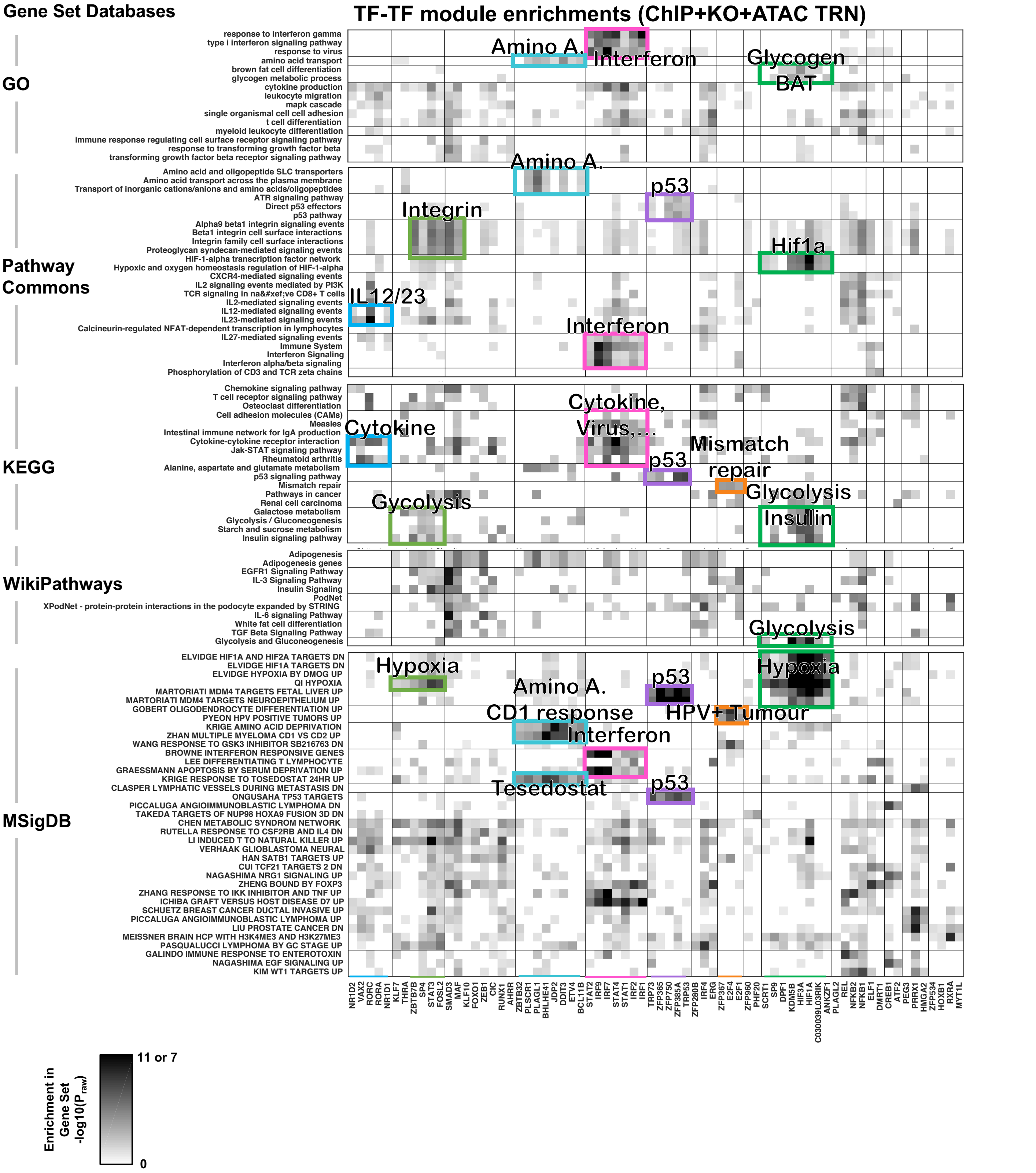

Figure S16. Gene-set enrichment of ChIP+KO+ATAC TRN TF modules. We compiled gene sets from five databases: GO, Pathway Commons, KEGG, MAPP and Signatures from MSigDB. For each TF, we calculated the significance of overlap between the TF's target genes and gene sets (hypergeometric CDF). For Kegg and Mapp databases, the color bar maximum is 7, and, for the other databases, the maximum is 11. TFs are clustered and color-coded according to the TF-TF modules in **Supplemental Fig. S14, S15, S17**. TF-TF module enrichments are consistent among gene sets and TRNs, providing robust predictions for modules in Th17 biology.
