# Supplementary material for: Leveraging chromatin accessibility for transcriptional regulatory network inference in T Helper 17 Cells
Source: Genome Res. 2019 Mar;29(3):449–63. doi: 10.1101/gr.238253.118 (PMC6396413; doi:10.1101/gr.238253.118)
Supplement: Supplemental Material [file supp_gr.238253.118_Supplemental_Fig_S18.pdf]

Figure S18

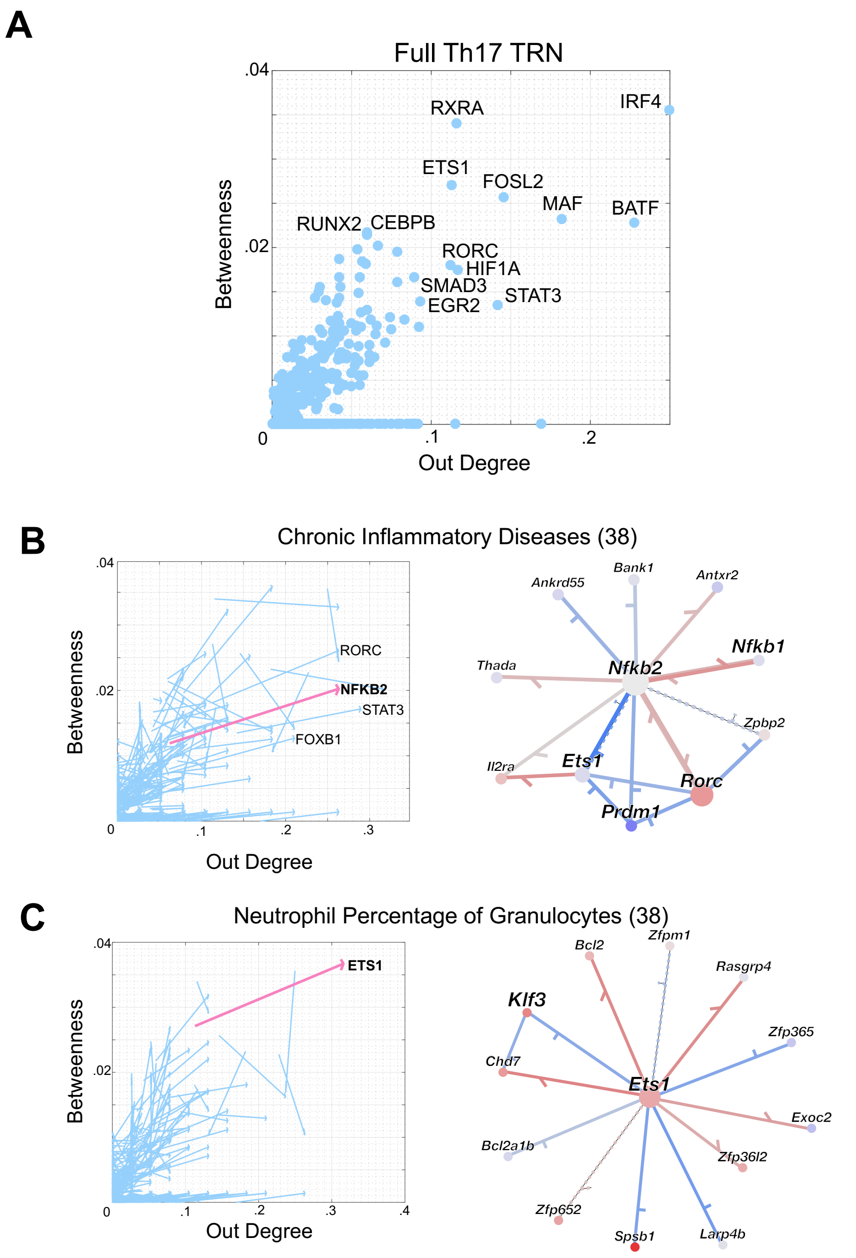

Figure S18. (A) Centrality analysis of the full Th17 TRN. TFs are plotted as a function of out degree (fraction of target genes regulated by the TF) and betweenness (fraction of shortest paths (from TFs to genes) containing the TF). **(B) NFKB2 targets are enriched in chronic inflammatory disease genes**. In the left panel, each arrow corresponds to a single TF. Arrow source is TF's centrality (out degree, betweenness) in the full Th17 TRN **(A)** and arrow points to the TF's centrality in the chronic inflammatory disease subnetwork (where target genes are limited to the 38 shared the Th17 TRN and GWAS set). NFKB2 (pink arrow) has significant increase in degree centrality (FDR=10%); other TFs (RORC, STAT3, FOXB1) also increase, but not significantly. The right panel features the subnetwork connecting NFKB2 to its target genes in the chronic inflammatory diseases. Node color indicates $\log_2$(fold-change) in Th17 48h condition relative to other Th timepoints (red = increased, blue = decreased), while red / blue indicate positive / negative regulation. Solid edges have support in the ChIP+KO+ATAC prior, while dotted edges do not. "Chronic inflammatory diseases" is an abbreviation of the trait "Chronic inflammatory diseases (ankylosing spondylitis, Crohn's disease, psoriasis, primary sclerosing cholangitis, ulcerative colitis) (pleiotropy)". **(C) ETS1 targets are enriched in the phenotype "neutrophil % of granulocytes" genes.** Analysis is displayed as in **(B)**, with the following exception: gene expression in the ETS1 subnetwork corresponds to the Th17 1h timepoint, as *Ets1* expression is highest at early timepoints (Th0 1h, Th17 1h and no media control timepoints).
