# Supplementary material for: Leveraging chromatin accessibility for transcriptional regulatory network inference in T Helper 17 Cells
Source: Genome Res. 2019 Mar;29(3):449–63. doi: 10.1101/gr.238253.118 (PMC6396413; doi:10.1101/gr.238253.118)
Supplement: Supplemental Material [file supp_gr.238253.118_Supplemental_Fig_S19.pdf]

Figure S19

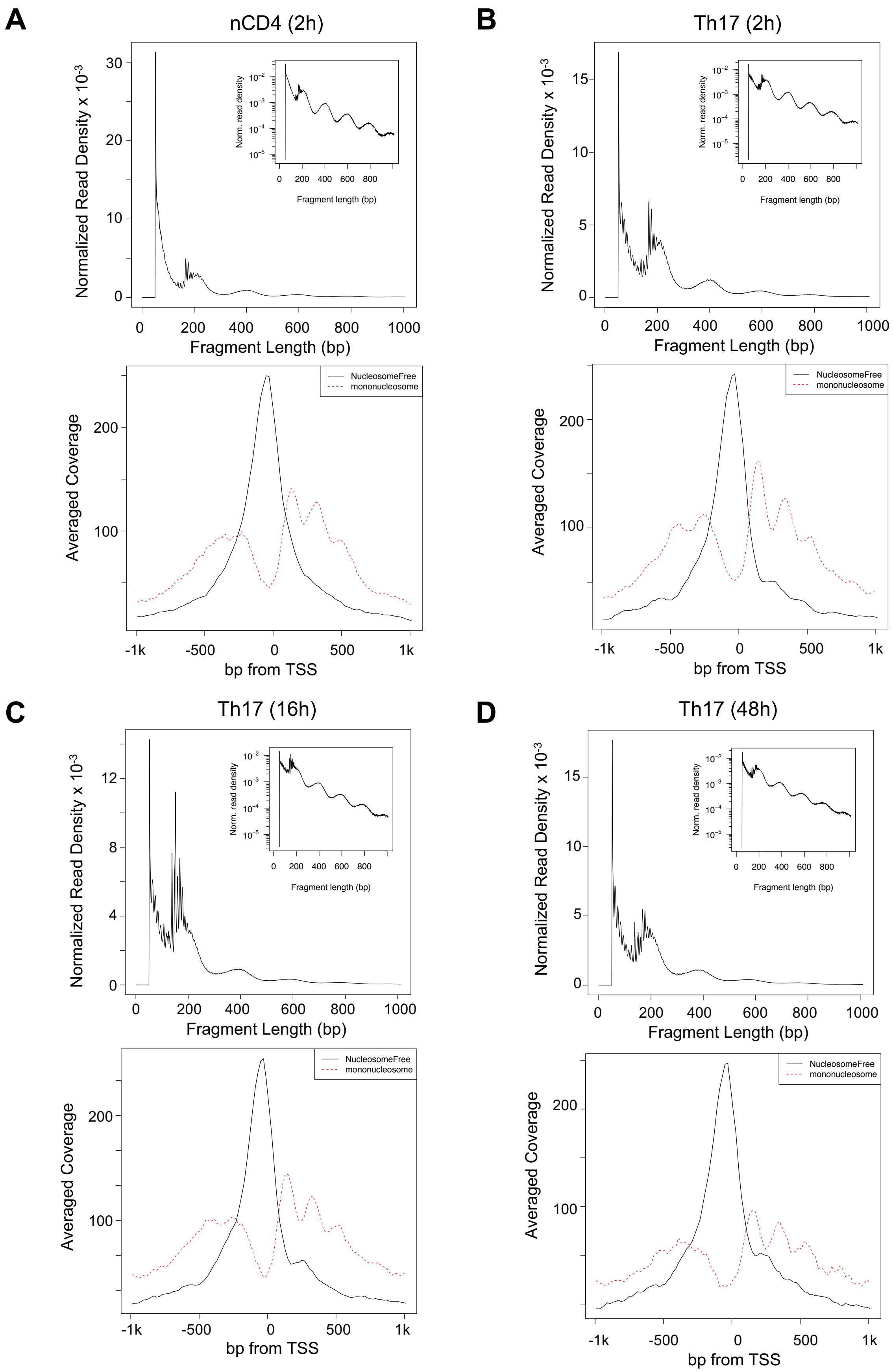

Figure S19. Representative outputs from ATACseqQC, displaying nucleosome-length periodicity in fragment lengths and signal distribution at the TSS for **(A)** naive CD4 T cells (2h), **(B)** Th17 (2h), **(C)** Th17 (16h), and **(D)** Th17 (48h).
