# Supplementary material for: Leveraging chromatin accessibility for transcriptional regulatory network inference in T Helper 17 Cells
Source: Genome Res. 2019 Mar;29(3):449–63. doi: 10.1101/gr.238253.118 (PMC6396413; doi:10.1101/gr.238253.118)
Supplement: Supplemental Material [file supp_gr.238253.118_Supplemental_Fig_S20.pdf]

Figure S20

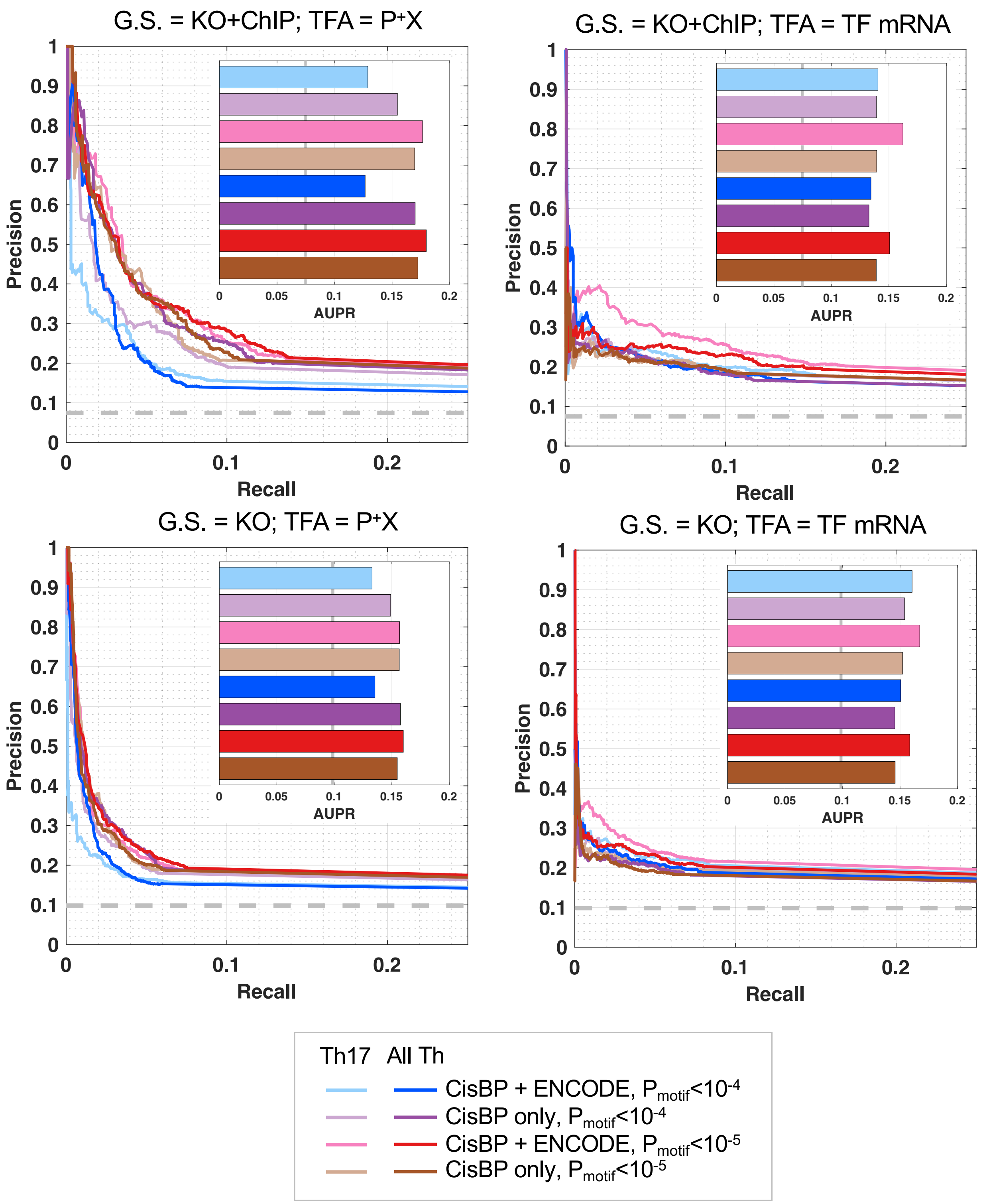

Figure S20. Comparison of CisBP with and without ENCODE-derived motifs. Priors were derived from the Th17 48h (Th17) or all T Helper (All Th) ATAC-seq samples using CIS-BP + human ENCODE motifs or CisBP. Two raw p-value cutoffs were tested. mLASSO-StARS was run with TF mRNA or prior-based TFA at bias = .5.
