# Supplementary material for: Leveraging chromatin accessibility for transcriptional regulatory network inference in T Helper 17 Cells
Source: Genome Res. 2019 Mar;29(3):449–63. doi: 10.1101/gr.238253.118 (PMC6396413; doi:10.1101/gr.238253.118)
Supplement: Supplemental Material [file supp_gr.238253.118_Supplemental_Fig_S21.pdf]

Figure S21

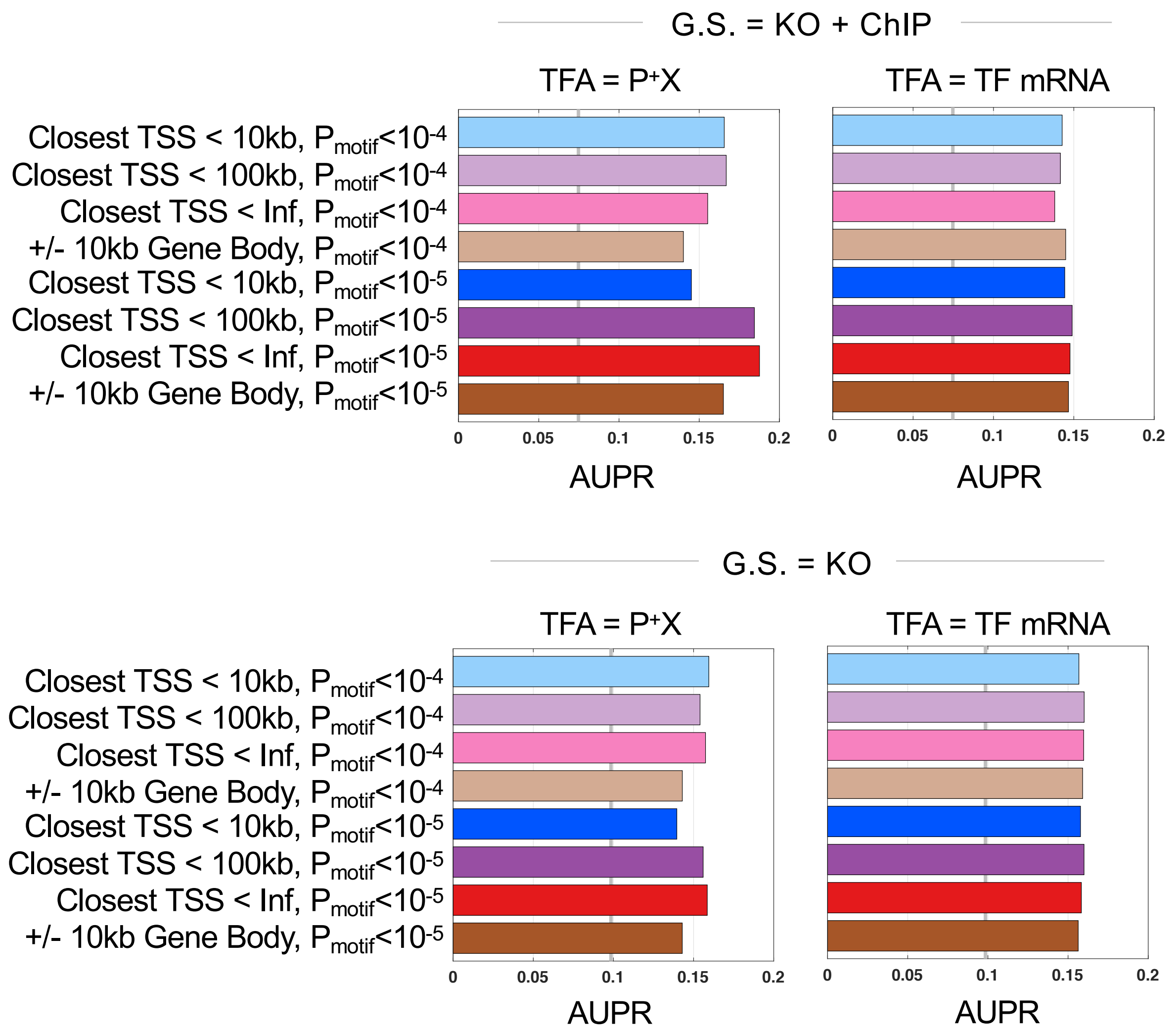

Figure S21. **Comparison of peak-gene association rules.** Th17 ATAC-seq priors were constructed using different peak-gene association rules and two raw p-value cutoffs for motifs from CisBP (note that ENCODE motifs were not included in this analysis). mLASSO-StARS was run with TF mRNA or prior-based TFA at bias = .5. (Inf = infinity.)
