# Supplementary material for: Leveraging chromatin accessibility for transcriptional regulatory network inference in T Helper 17 Cells
Source: Genome Res. 2019 Mar;29(3):449–63. doi: 10.1101/gr.238253.118 (PMC6396413; doi:10.1101/gr.238253.118)
Supplement: Supplemental Material [file supp_gr.238253.118_Supplemental_Fig_S22.pdf]

Figure S22

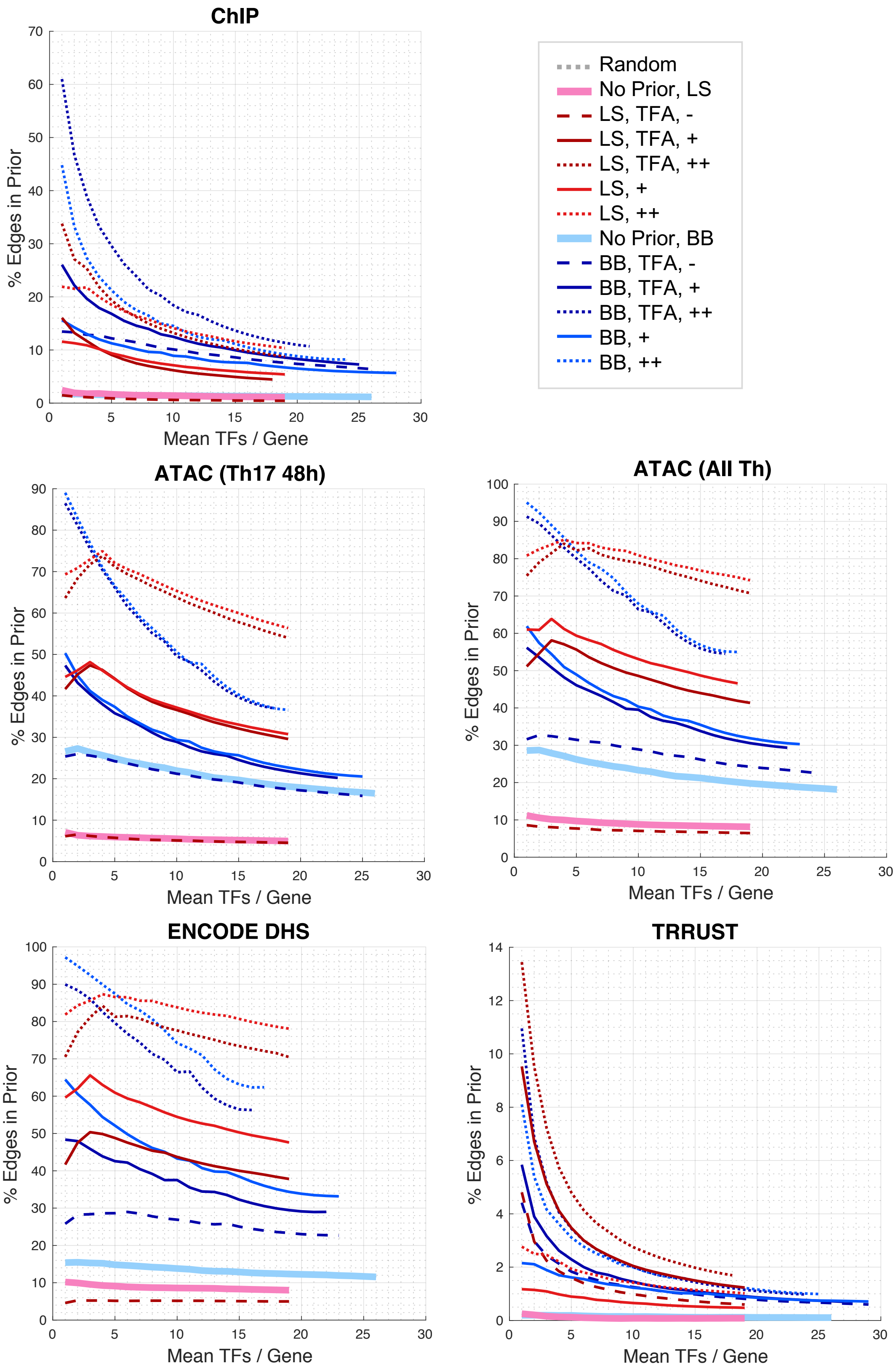

Figure S22. The effect of TFA and prior reinforcement on recovery of prior edges in TRN models. % prior edges in the TRNs are plotted as a function of TRN size (mean TF predictors per gene) for the two Inferelator methods (BBSR-BIC and mLASSO-StARS), the two methods for estimating TF activities (TFA = P+X and TF mRNA), and varying levels of prior reinforcement (-, +, ++ correspond to no, moderate, and high prior reinforcement, respectively).
