# Supplementary material for: Leveraging chromatin accessibility for transcriptional regulatory network inference in T Helper 17 Cells
Source: Genome Res. 2019 Mar;29(3):449–63. doi: 10.1101/gr.238253.118 (PMC6396413; doi:10.1101/gr.238253.118)
Supplement: Supplemental Material [file supp_gr.238253.118_Supplemental_Fig_S23.pdf]

Figure S23

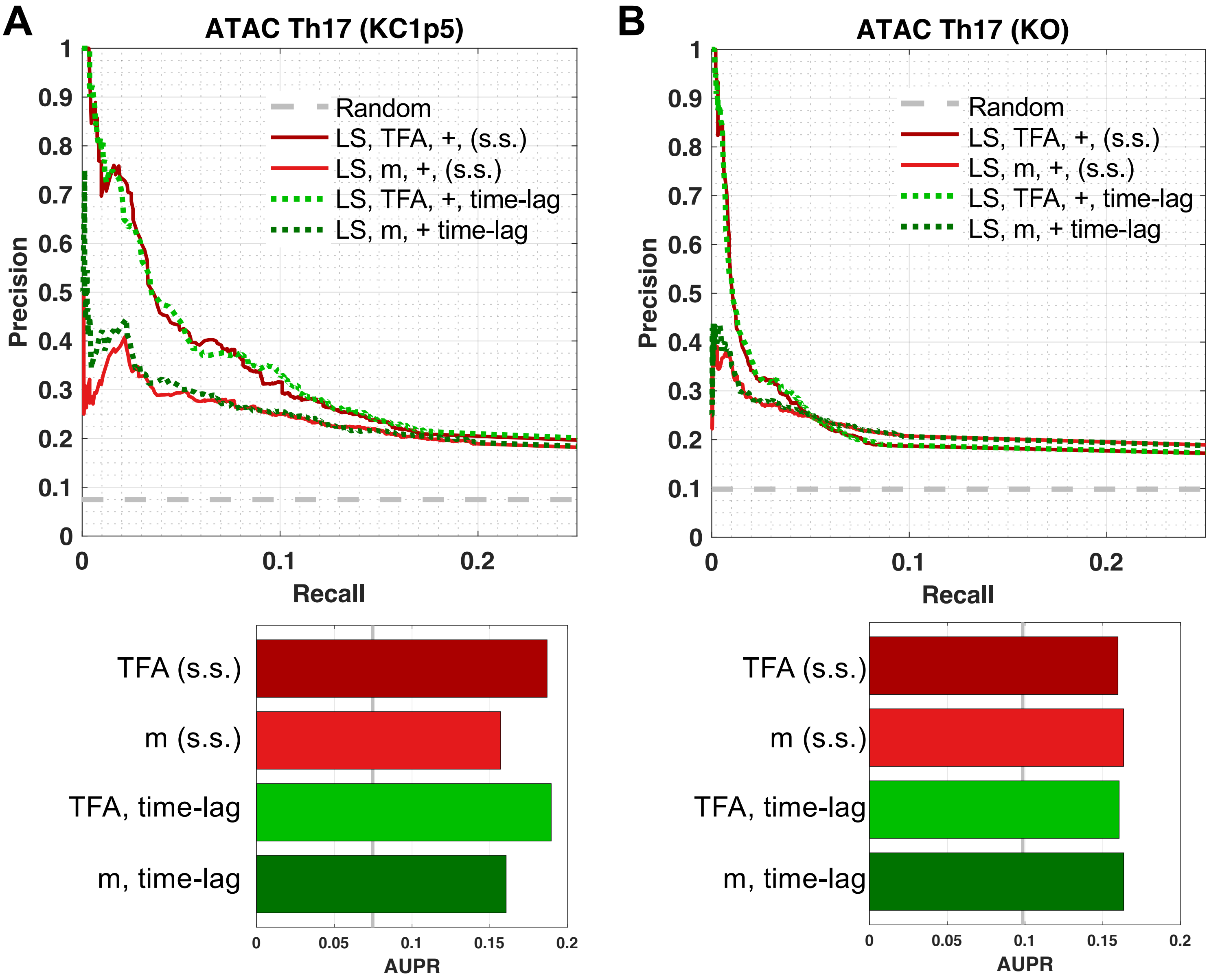

Figure S23. **Comparison of steady-state versus time-lagged treatment of time-series data.** The gene expression matrix includes a small fraction of time-series samples (15 out of 254). Using the time-lag parameter tau (30 minutes) from (Ciofani et al. 2012), we compared performance between TRNs built with and without time-lag (mLASSO-StARS with the Th17 ATAC prior, moderate prior reinforcement (bias = .5), and either TFA = TF mRNA or prior-based TFA. Precision-recall of the **(A)** KO+ChIP and **(B)** KO G.S.'s did not change between time-lagged and steady-state models.
