# Supplementary material for: Leveraging chromatin accessibility for transcriptional regulatory network inference in T Helper 17 Cells
Source: Genome Res. 2019 Mar;29(3):449–63. doi: 10.1101/gr.238253.118 (PMC6396413; doi:10.1101/gr.238253.118)
Supplement: Supplemental Material [file supp_gr.238253.118_Supplemental_Fig_S24.pdf]

Figure S24

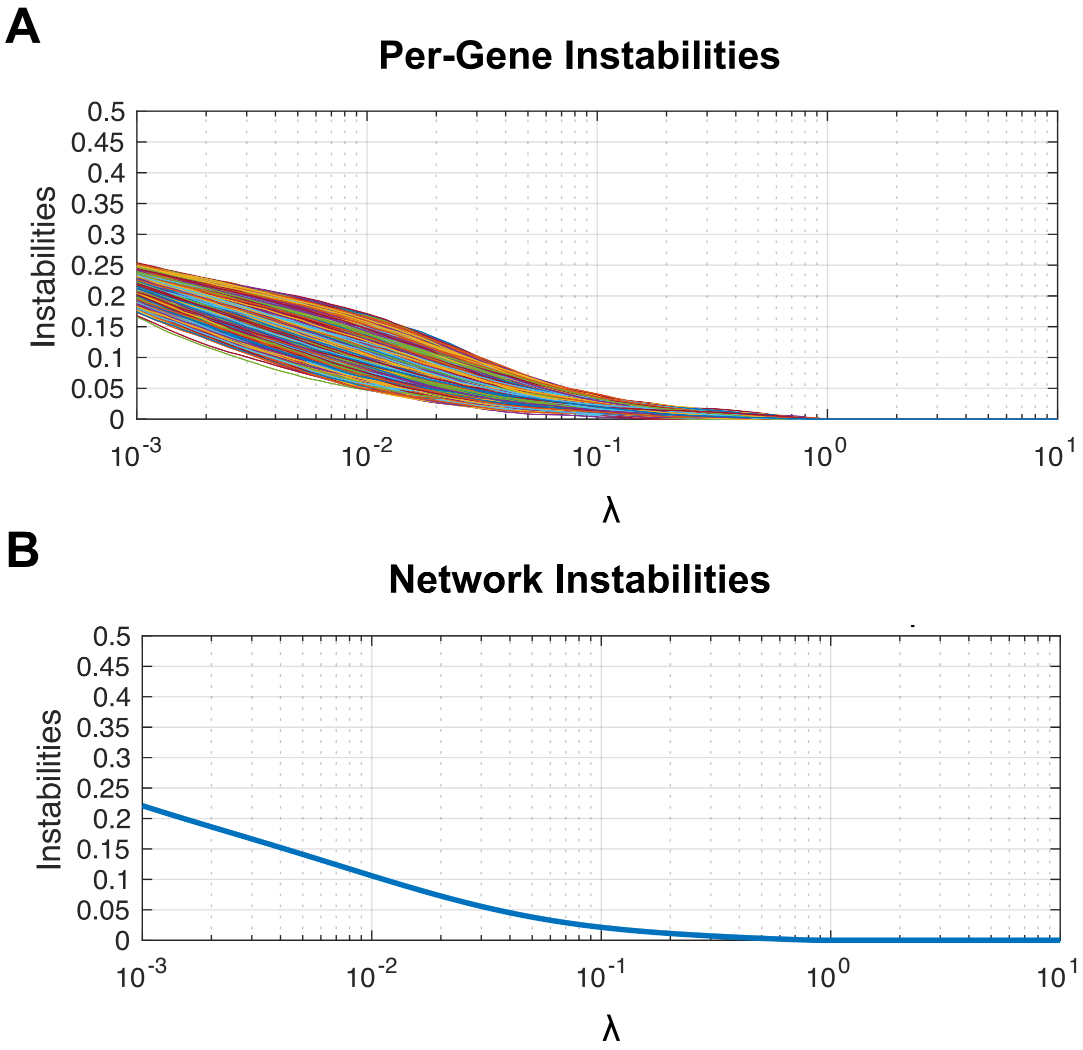

Figure S24. **(A) Per-gene and (B) network-level average instability paths.** Average instabilities were calculated on per-gene and network level over a range of LASSO penalties, λ. Results are shown for the No Prior TRN using 50 subsamples.
