# Supplementary material for: Leveraging chromatin accessibility for transcriptional regulatory network inference in T Helper 17 Cells
Source: Genome Res. 2019 Mar;29(3):449–63. doi: 10.1101/gr.238253.118 (PMC6396413; doi:10.1101/gr.238253.118)
Supplement: Supplemental Material [file supp_gr.238253.118_Supplemental_Fig_S25.pdf]

Figure S25

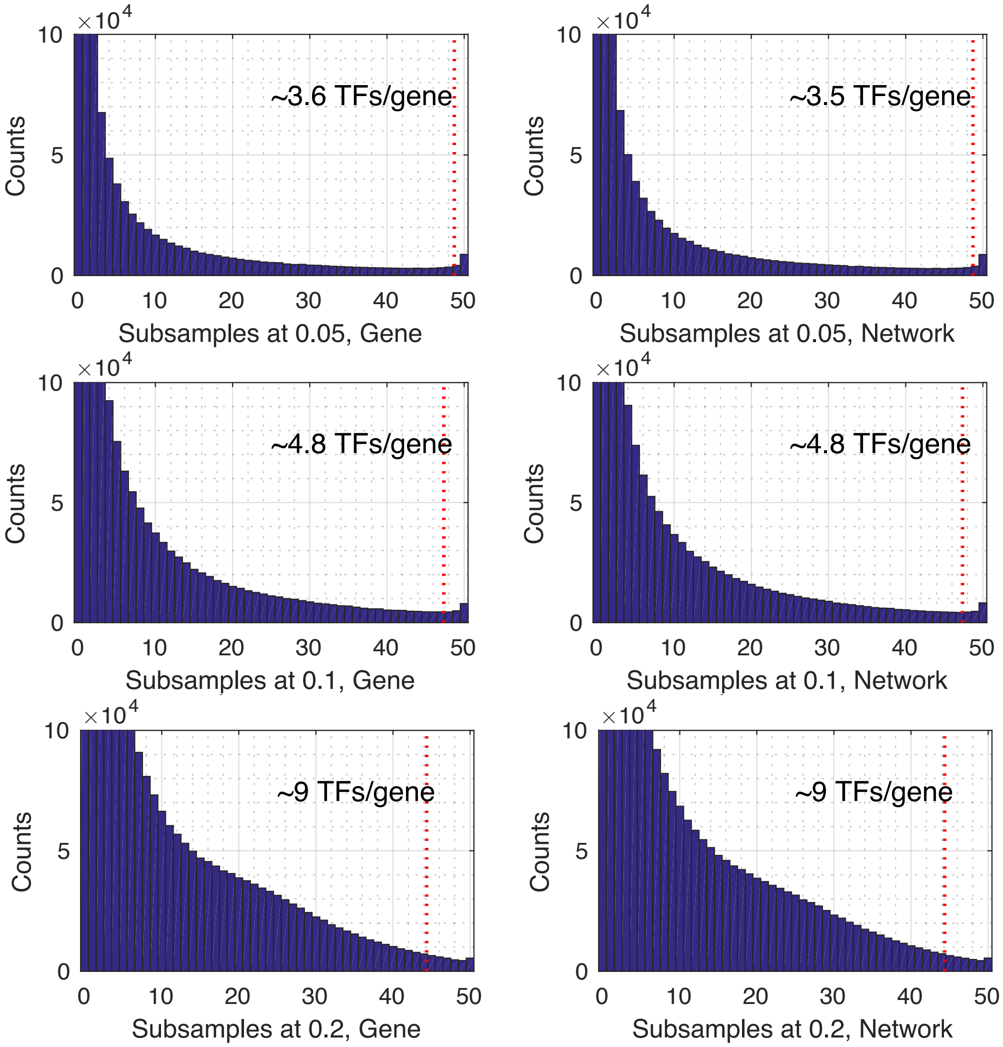

Figure S25. **Distribution of nonzero subsamples per TF-gene edge.** Distribution of nonzero subsamples per TF-gene interaction, using per-gene and network average instability cutoffs of .05, .1, and .2. Results are shown for the No Prior TRN using 50 subsamples. The dotted-red line marks the instability cutoff in terms of nonzero subsamples, and the average model size at that cutoff appears as text in the upper right hand.
