# Supplementary material for: Leveraging chromatin accessibility for transcriptional regulatory network inference in T Helper 17 Cells
Source: Genome Res. 2019 Mar;29(3):449–63. doi: 10.1101/gr.238253.118 (PMC6396413; doi:10.1101/gr.238253.118)
Supplement: Supplemental Material [file supp_gr.238253.118_Supplemental_Fig_S26.pdf]

Figure S26

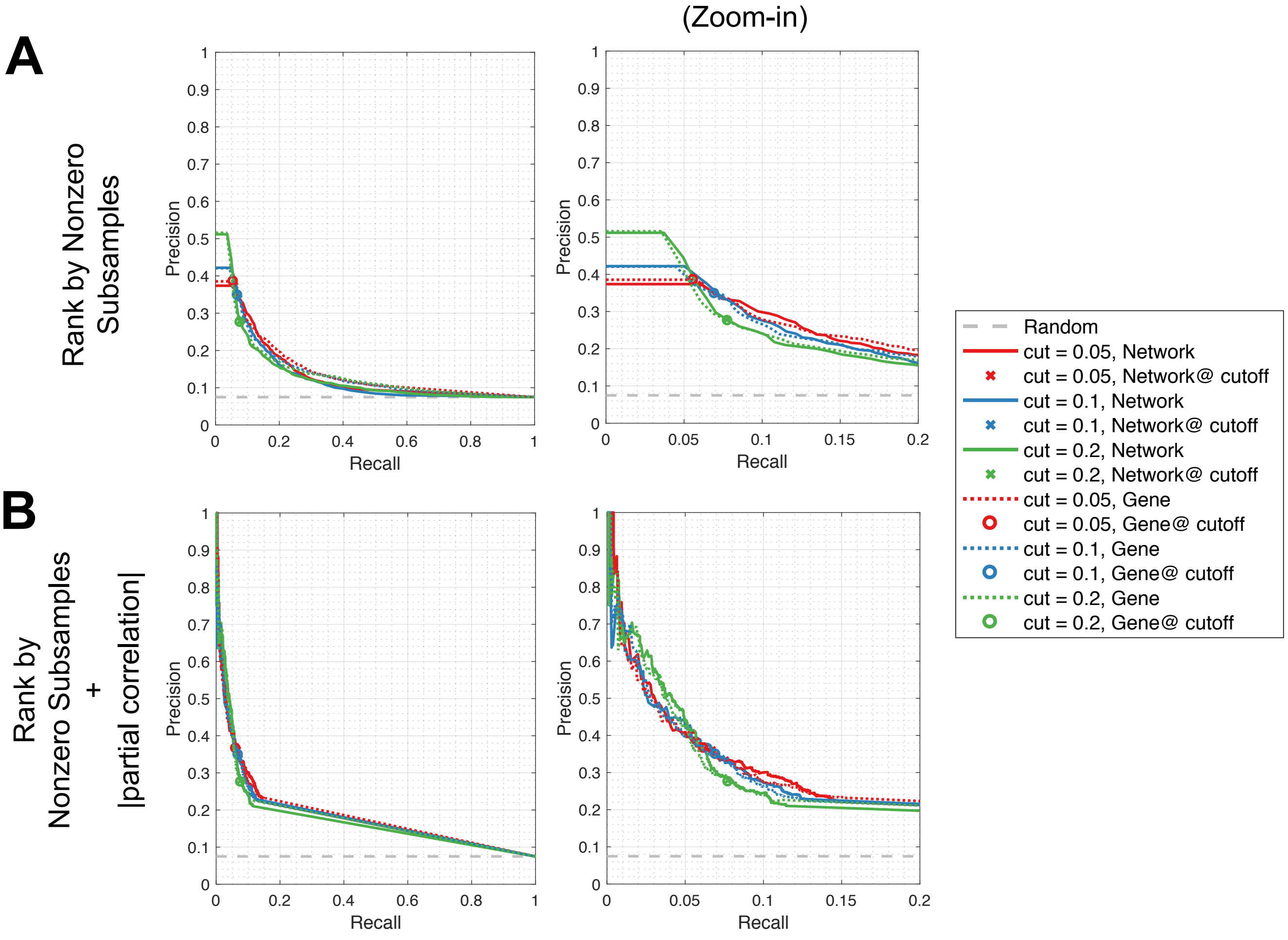

Figure S26. **Comparison of edge-ranking methods**. Edges are ranked according to **(A)** nonzero subsamples or **(B)** nonzero subsamples plus the absolute value of edge partial correlation (**Equation 4**) for network- and gene-level average instabilities at cutoffs .05, .1 and .2. Right and left panels denote full and zoomed-in curves, respectively. Results are shown for the Th17 ATAC prior, bias = .5, and prior-based TFA, using the KO + ChIP G.S.
