# Supplementary material for: Leveraging chromatin accessibility for transcriptional regulatory network inference in T Helper 17 Cells
Source: Genome Res. 2019 Mar;29(3):449–63. doi: 10.1101/gr.238253.118 (PMC6396413; doi:10.1101/gr.238253.118)
Supplement: Supplemental Material [file supp_gr.238253.118_Supplemental_Fig_S28.pdf]

Figure S28

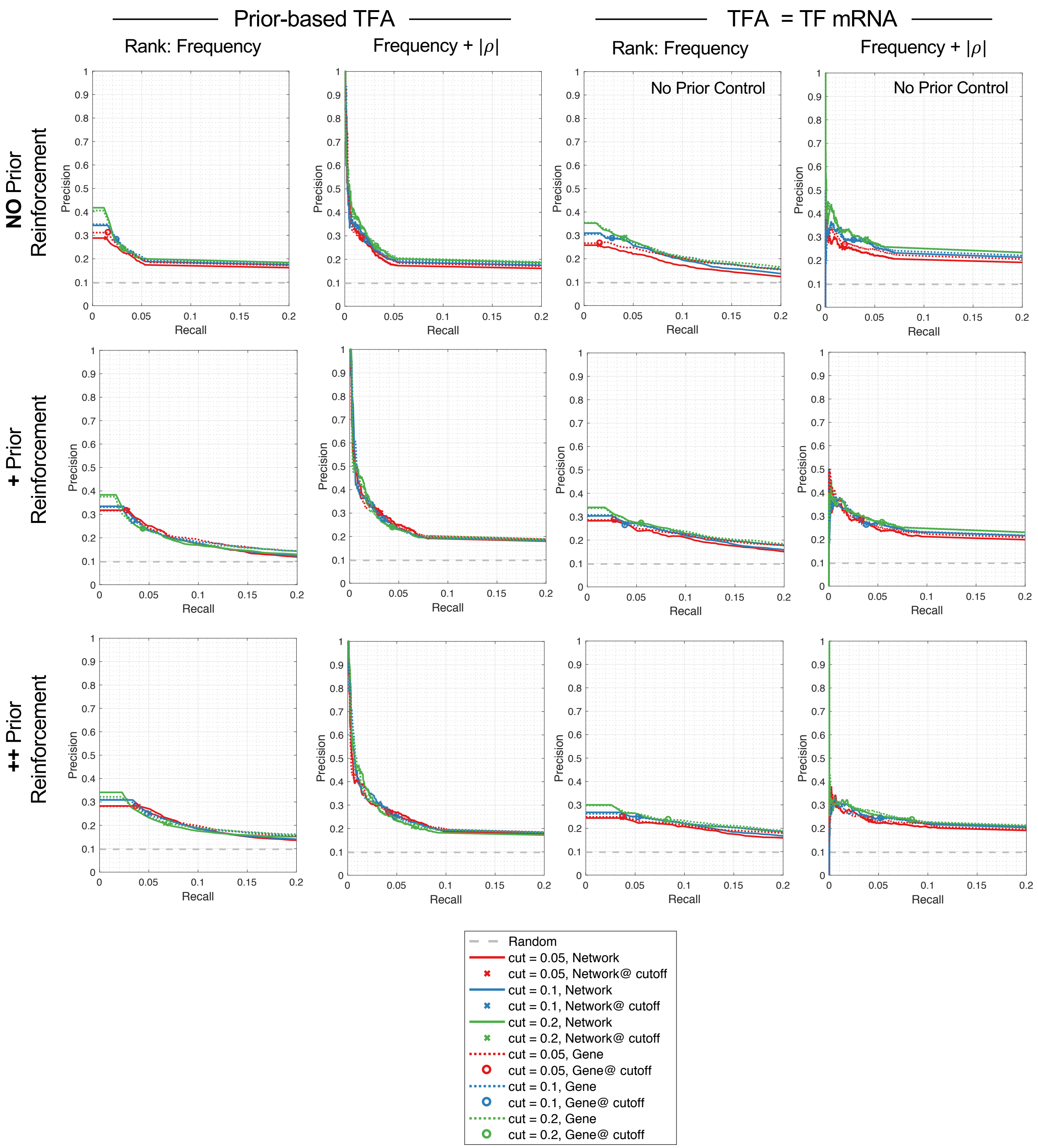

Figure S28. **StARS parameterization: precision-recall of the KO G.S.** TRNs were built using the Th17 ATAC prior with prior-based or TF mRNA TFA, no, moderate or strong prior reinforcement, network- and gene-level average instabilities at cutoffs .05, .1 and .2. Edges are ranked according to nonzero subsamples or **Equation 4**.
