# Supplementary material for: Leveraging chromatin accessibility for transcriptional regulatory network inference in T Helper 17 Cells
Source: Genome Res. 2019 Mar;29(3):449–63. doi: 10.1101/gr.238253.118 (PMC6396413; doi:10.1101/gr.238253.118)
Supplement: Supplemental Material [file supp_gr.238253.118_Supplemental_Fig_S29.pdf]

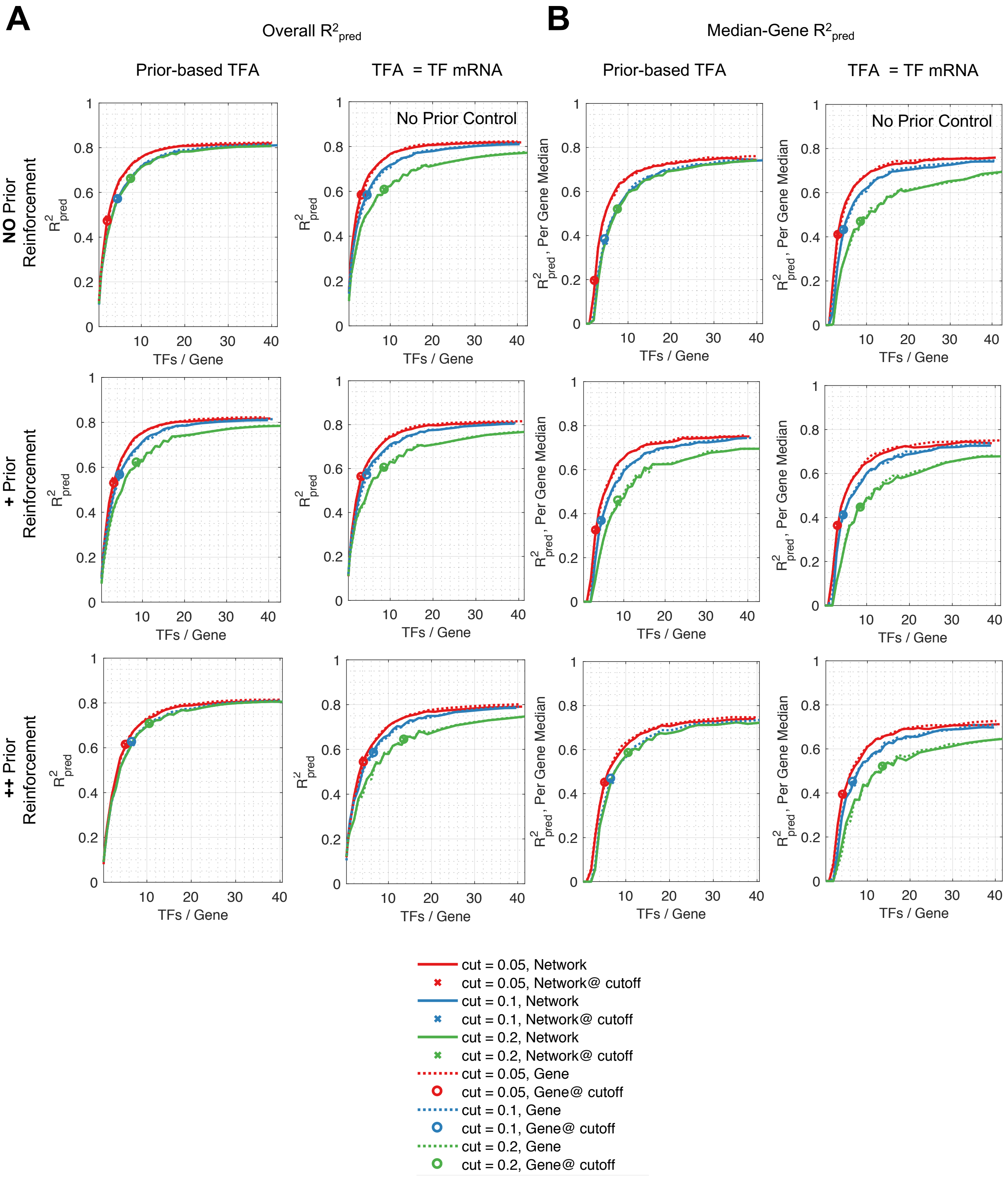

Figure S29. **StARS parameterization: out-of-sample gene expression prediction.** TRNs were built using the Th17 ATAC prior with prior-based or TF mRNA TFA, no, moderate or strong prior reinforcement, network- and gene-level average instabilities at cutoffs .05, .1 and .2. Out-of-sample gene expression prediction was tested on the leave-out "Early Th17" set (8 samples). **(A)** and **(B)** show overall R^2_{pred} or the median-gene's R^2_{pred} as a function of average model size, using **Equation 4** to rank TF-gene interactions.
