# Supplementary material for: Leveraging chromatin accessibility for transcriptional regulatory network inference in T Helper 17 Cells
Source: Genome Res. 2019 Mar;29(3):449–63. doi: 10.1101/gr.238253.118 (PMC6396413; doi:10.1101/gr.238253.118)
Supplement: Supplemental Material [file supp_gr.238253.118_Supplemental_Fig_S30.pdf]

Figure S30

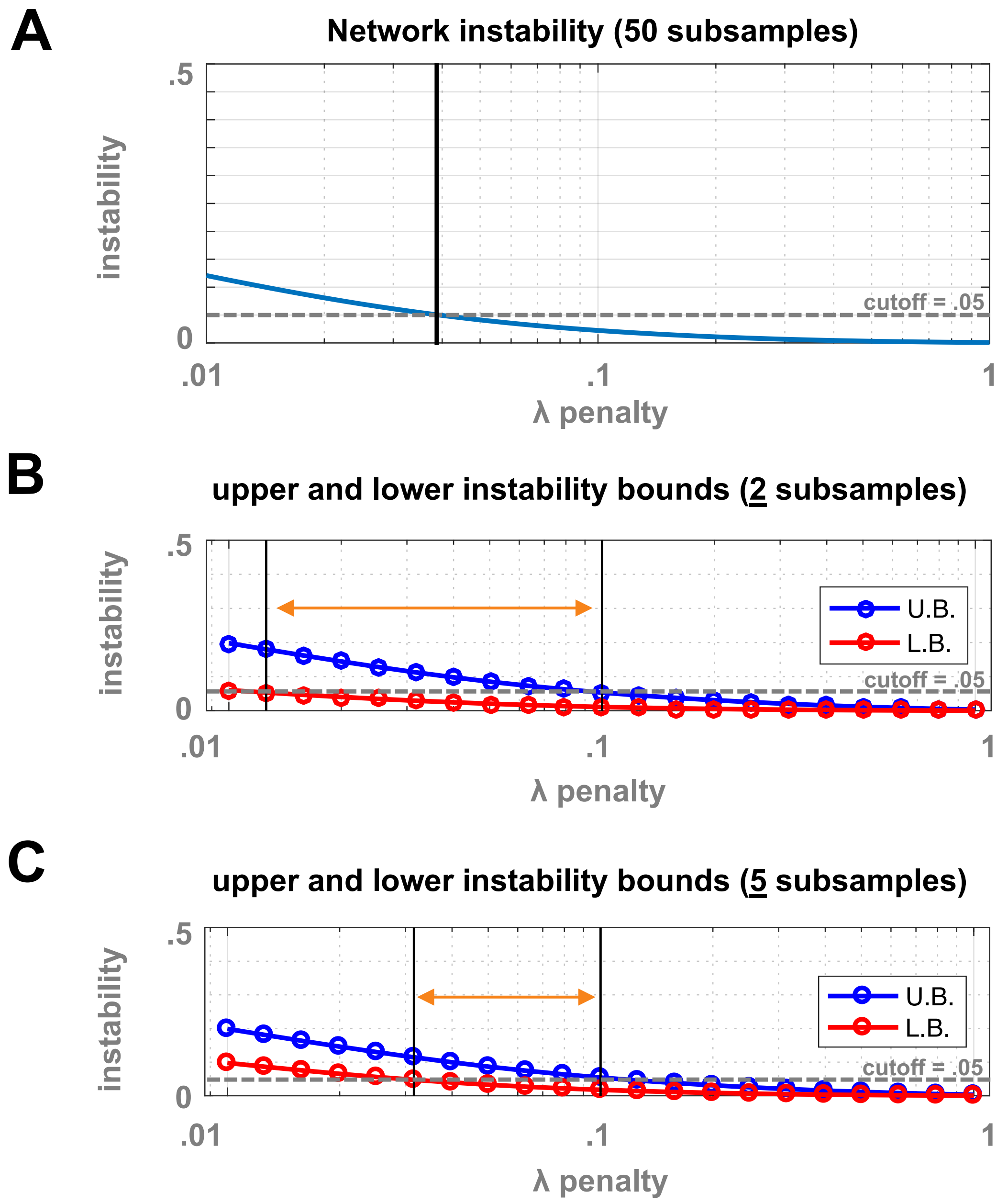

Figure S30. **Computational speed-up with bStARS lambda bounds. (A)** Network instabilities were estimated using 50 subsamples for Th17 ATAC prior with prior-based TFA and moderate reinforcement over a range of lambda penalties containing the lambda corresponding to the target instability cutoff .05 (vertical black line). bStARS upper and lower instability bounds, based on **(B)** two or **(C)** five subsamples shorten the lambda search space (orange arrows, and black lines indicate lower and upper bounds for lambda corresponding to the target instability cutoff .05). Solving the LASSO is very slow for smaller lambda penalties; thus, increasing the lower bound (as in **(C)**) results in significant speed-up (~2-fold), while looser bounds (as in **(B)**) do not.
