# Supplementary material for: Leveraging chromatin accessibility for transcriptional regulatory network inference in T Helper 17 Cells
Source: Genome Res. 2019 Mar;29(3):449–63. doi: 10.1101/gr.238253.118 (PMC6396413; doi:10.1101/gr.238253.118)
Supplement: Supplemental Material [file supp_gr.238253.118_Supplemental_Fig_S31.pdf]

Figure S31

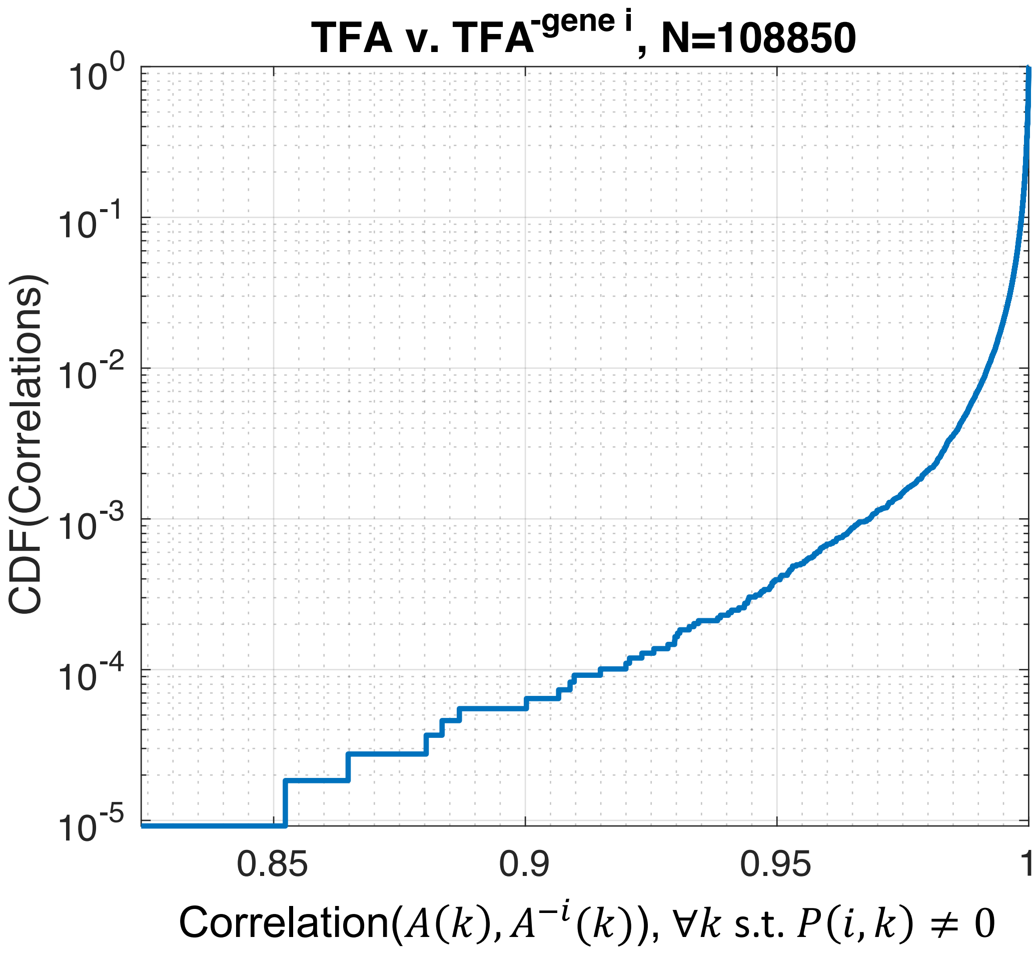

Figure S31. **Effect of individual target genes on prior-based TFA estimation.** The empirical cumulative distribution function (CDF) of Pearson correlation coefficients between TFA estimates from **Equation 2** versus **Equation 6**, using the Th17 ATAC prior. For each of the 2093 target genes with edges in the prior P, **Equation 6** is solved for A^{-i}. For each gene i, correlations are limited to those TFs k for which $\mathrm{P(i,k)} \neq 0$, yielding 108,850 correlation coefficients.
