# Supplementary material for: Leveraging chromatin accessibility for transcriptional regulatory network inference in T Helper 17 Cells
Source: Genome Res. 2019 Mar;29(3):449–63. doi: 10.1101/gr.238253.118 (PMC6396413; doi:10.1101/gr.238253.118)
Supplement: Supplemental Material [file supp_gr.238253.118_Supplemental_Fig_S32.pdf]

# Figure S32

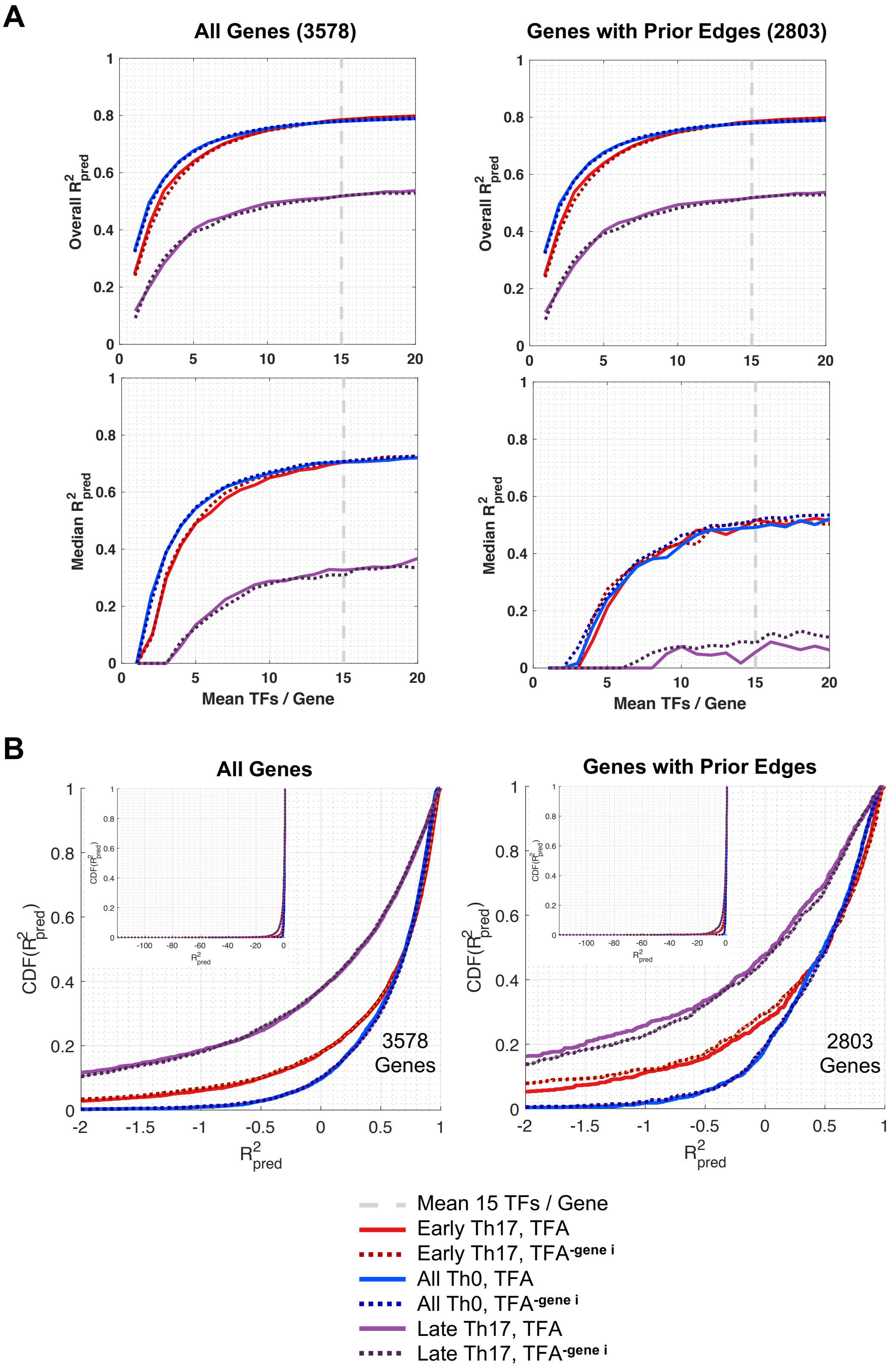


**Figure S32**. **Effect of individual target genes on prediction using prior-based TFA.** For the three out-of-sample prediction challenges, gene models were built using **Equation 2** ("TFA") or **Equation 6** ("TFA$^{-gene\ i}$"), using mLASSO-StARS, Th17 ATAC prior, and moderate prior reinforcement. **(A)** Overall $R^2_{pred}$ and median per-gene $R^2_{pred}$ for all genes or limited to those genes with edges in the prior. **(B)** The CDFs of per-gene $R^2_{pred}$ are plotted for average model size of 15 TFs/gene. Thus, in this context, evaluation of out-of-sample gene expression prediction can be accomplished using **Equation 2** (versus the more computationally intensive **Equation 6**).
