# Supplementary material for: Leveraging chromatin accessibility for transcriptional regulatory network inference in T Helper 17 Cells
Source: Genome Res. 2019 Mar;29(3):449–63. doi: 10.1101/gr.238253.118 (PMC6396413; doi:10.1101/gr.238253.118)
Supplement: Supplemental Material [file supp_gr.238253.118_Supplemental_Fig_S33.pdf]

Figure S33

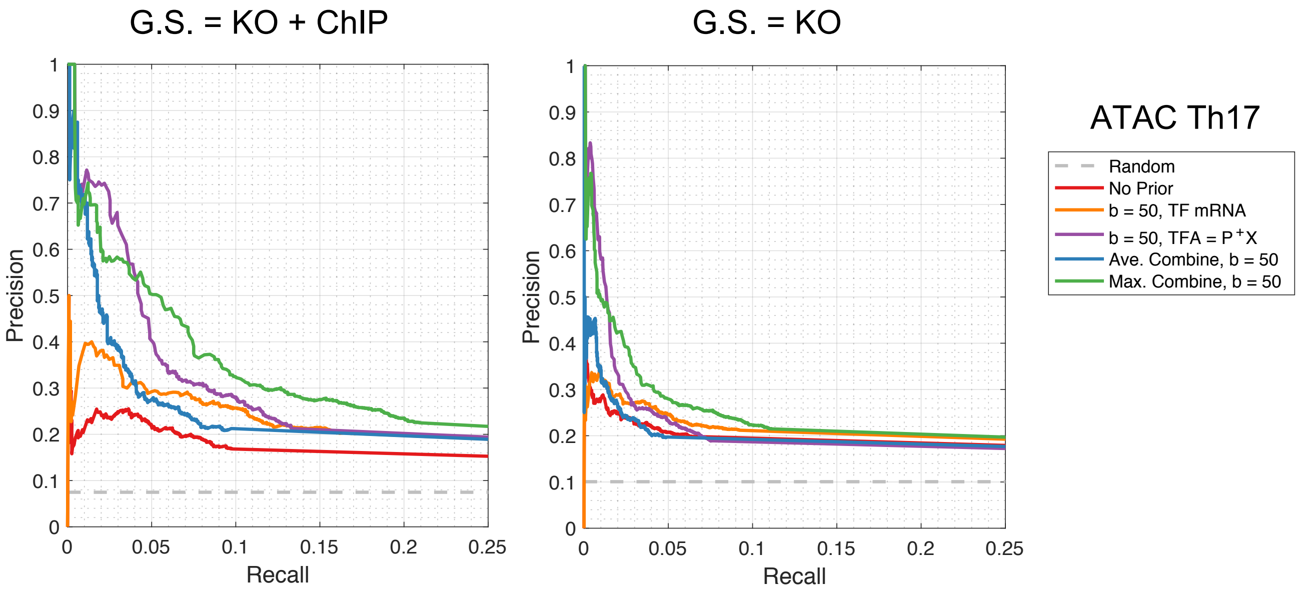

Figure S33. **Rank-combination of ATAC TRNs.** The precision-recall of individual ATAC TRNs (at moderate prior reinforcement "b=50" for prior-based and TF mRNA TFA) are compared to performance average- or maximum-combination of TRNs.
