# Supplementary material for: Leveraging chromatin accessibility for transcriptional regulatory network inference in T Helper 17 Cells
Source: Genome Res. 2019 Mar;29(3):449–63. doi: 10.1101/gr.238253.118 (PMC6396413; doi:10.1101/gr.238253.118)
Supplement: Supplemental Material [file supp_gr.238253.118_Supplemental_Fig_S35.pdf]

A

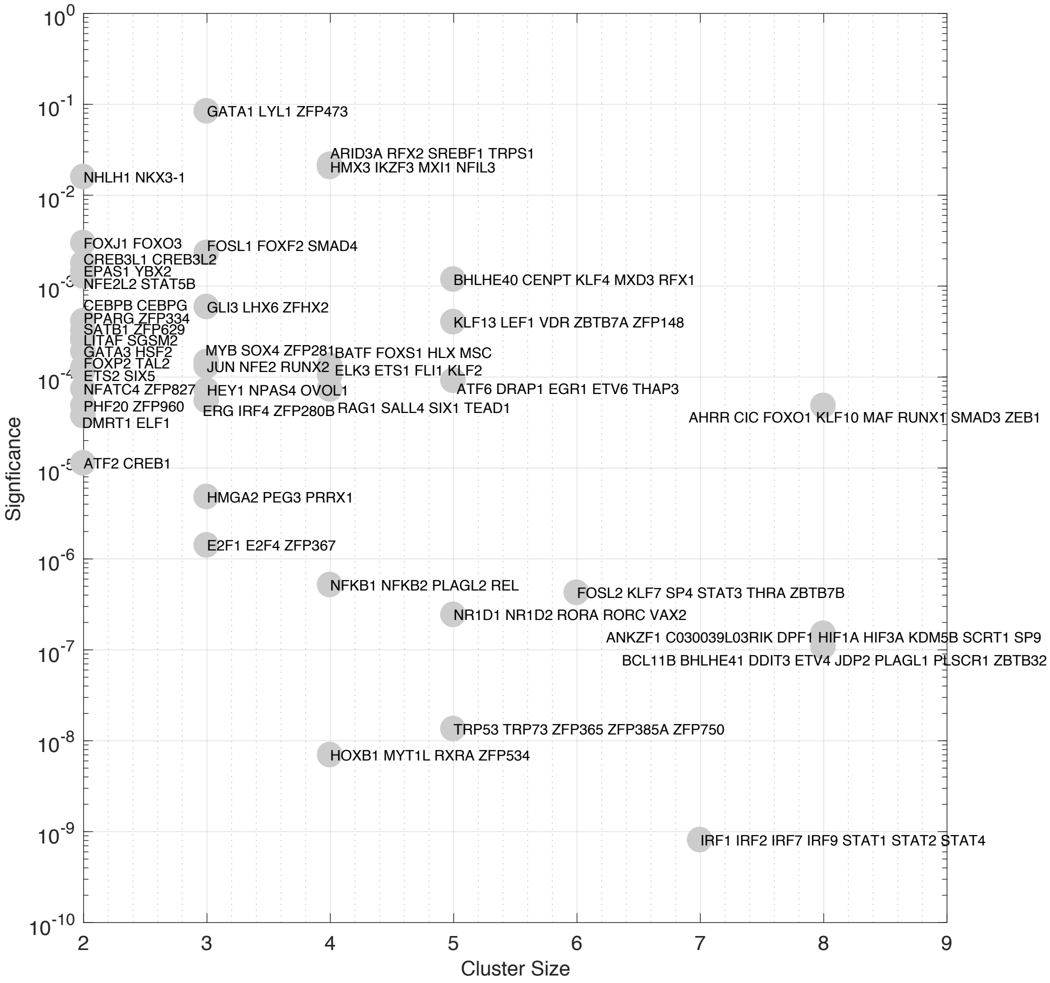

B

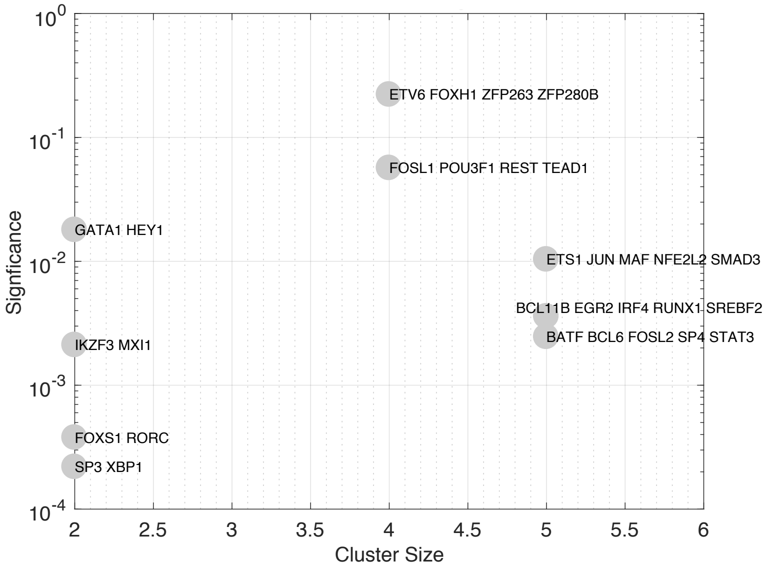

Figure S35. **Ranking of TF-TF Clusters.** Heuristic significance estimates versus cluster size for TF-TF modules built from **(A)** positive and **(B)** negative TF-TF edge overlaps. Results are shown for the "final" ChIP+KO+ATAC TRN.
