# Supplementary material for: Leveraging chromatin accessibility for transcriptional regulatory network inference in T Helper 17 Cells
Source: Genome Res. 2019 Mar;29(3):449–63. doi: 10.1101/gr.238253.118 (PMC6396413; doi:10.1101/gr.238253.118)
Supplement: Supplemental Material [file supp_gr.238253.118_Supplemental_jp_gene_viz.zip › jp_gene_viz-master/bioRxiv/jp_gene_viz.pdf]

Network visualizations in reproducible workflows using Jupyter

Author 1[1], Author 2[2], Author 3[1], Author 4[1], Author 5[2], Author 6[2], Author 7[1,*]

1 Affiliation A
2 Affiliation B

*** correseponding@author.mail**

Network visualizations in reproducible workflows using Jupyter

April 4, 2017

Abstract

Transcriptional regulatory networks (TRNs) describe how gene expression is regulated in diverse cell types, tissues, and organisms in response to environmental cues. We describe a methodology for sharing executable workflows for deriving, comparing, and analysing TRNs and related gene expression data. The methodology uses archives orchestrated by Jupyter notebook narrative documents and allows for seamless integration of visualizations with R and Python programs used to derive TRNs and process genomics data. These documents include key interactive visualization tools designed to help researchers explore and understand TRNs and the relationship of TRNs to gene expression matrices and other data. The workflows run on any platform in standard web browsers.

1 Introduction

Many experimental designs today aim to derive network models of regulation of gene transcription [1, 3, 13, 23] from high throughput transcriptomic sequencing data [20] and other genomics technologies or data sources. These studies frequently involve several researchers working in different locations who have different expertise, from experimental methods to computational inference of TRNs [4, 21].

This paper describes a methodology for documenting the derivation, analysis, and visualization of TRNs and for comparing TRNs to gene expression data and other evidence using repositories containing Jupyter notebook documents [19] and other source files. These workflows include widgets that implement interactive visualizations designed to present and explore TRNs, implemented in the *jp_gene_viz* project [22]. All notebook interactions including interactive visualizations can be completely automated by the notebook author using simple script fragments allowing other researchers such as collaborators, reviewers, or auditors to reproduce all research results. The notebooks of the archive can be executed by researchers remotely using cloud web services such as *binder* [16] without the need to install any special purpose software on the local machine.

2 Related Work

There are many tools for visualizing TRNs [8] including stand alone applications [2, 5, 17] and special purpose web applications [24]. Other general network visualization tools use sophisticated query models to help users explore network models in many domains [12].

This work presents an alternative visualization methodology which is designed to be used in Jupyter based workflows. This paper focusses on the visualization widgets in

jp_gene_viz specifically designed to present and explore TRNs and we limit our attention to Jupyter notebooks driven by the R and Python Jupyter kernels. The interactions provided by the widgets we describe include dozens of operations requested by researchers interested in deriving and analysing TRNs and related data. Notebook narratives may include preprocessing for the upstream inputs to the visualization widgets and may extract the results derived from visualization transformations and use those outputs in downstream processing. These network visualizations inputs and outputs may be freely combined with other components of the Jupyter ecosystem such as statistical classification tools provided by scikit-learn [18] and other mathematical tools provided by SciPy [11] for Python based notebooks, or the Bioconductor packages for R based notebooks [9]. The outputs may also be fed to subprocesses controlled from the Jupyter notebook document.

Other visualizations can be used in combination with the jp_gene_viz widgets to present the data in alternate ways in the Jupyter environment such as the matplotlib plotting library [10] and any Javascript based HTML5 library such as the Cytoscape.js [7] network display library or the Highcharts chart libraries [14].

3 Building reproducible workflow archives with interactive visualizations

Workflow archives include source data and computations for generating output data as well as visualizations or other derivative products. The archives should explain the operation and justifications for each step of the workflow. These requirements are easily implemented using folders containing Jupyter notebooks and required data files.

Jupyter notebooks consist of a sequence of documentation cells and executable cells followed by static text or visualization results or by interactive widget visualizations. Executable notebook cells support general purpose computation using Python or R script fragments, subprocesses, and shell command lines. A workflow should organize all high level operations as a sequence of notebooks which perform all operations of the workflow and include discussion text explaining each step of the workflow formatted using documentation cells. An "index" notebook should provide an entry point with introductory information and links to the other notebooks of the workflow. The actions of each notebooks should be idempotent in the sense the notebook produces the same output artifacts whenever it is executed with the same inputs unless the notebook is modified by the user in some manner.

To construct a workflow archive a researcher assembles required input files into an archive folder and then constructs notebooks for generating outputs from the inputs as well as any visualizations.

Visualizations are typically constructed initially in an interactive manner by direct manipulation of interactive controls which are later emulated using script fragments to make the visualization reproducible. For example a slider adjusted to level 10 followed by a button press can be emulated using the script fragment `network.threshold_slider.value=10; network.expand_button.click())`.

Once the researcher has captured the workflow process as a sequence of executable notebook narratives, with visualization configuration hardened into code cell script fragments, the archive must be packaged for distribution. We capture all software dependencies for a workflow by building a Dockerfile designed to build a Docker container [15] that will run the workflow archive in the Binder cloud web service [16] to allow collaborators to recreate the software infrastructure for the workflows. The Dockerfile usually uses other configuration files in the build process.

To publish the workflow archive we create a public Github project including all data

and executable notebooks and any other dependencies. In some cases we add encryption/decryption to the workflow to prevent releasing data which is not appropriate for release to unauthorized parties. At this point we usually build the Github repository in the the Binder service [16] to make the archive available easily to other researchers without the need to install software and also to test that the Dockerfile captures all dependencies. We then share the location of the Github repository for the workflow and a hyperlink for running the archive in Binder with collaborators and other researchers.

4 The network visualization components

The jp_gene_viz package includes several interactive Jupyter widgets for visualizing and manipulating TRN data and relate gene expression matrices. It is not possible to discuss all of the features of these widgets in this short paper – please see the package documentation for further details. All of these widgets accept and generate standard data formats as inputs and outputs. The programming interface to the widgets may be used to implement interfaces to other formats and data sources using Jupyter cell script fragments.

The TRN Network interactive visualization is illustrated in the top part of Figure 1. The network consists of a directed weighted graph between nodes labeled with gene names and make contain thousands of nodes and edges. An edge of the graph connects a transcription factor gene (TF) with a gene influenced by that factor where a positive weight (shown in shades of red) indicates that the TF promotes gene transcription of the target gene or a negative weight (shown in shades of blue) indicates the TF inhibits transcription of the gene. Nodes and edges of the graph may also be associated with other information such as node weights and edge binding motifs. Usually the visualization displays a subset of the nodes and edges of the network and many of the interactive controls allow the researcher to select the subset of interest and adjust the way the subset is presented. This widget includes dozens of interactive features not described here including a hidden panel which allows network display configurations to be exported to various formats for publication and archived. For example, a network can be annotated with sequence motifs [6] associated with transcriptional relationships as illustrated in Figure 2. The network widget also supports interactive features for dragging/dropping subsets of the network to improve layout as well as customization of colors and label sizes. These features were requested by genomics researchers using the widget in workflows.

A TRN network visualization may be linked with a heatmap showing gene expression levels under different experimental conditions as shown in Figure 1. The heatmap shows values for each gene as rows and values for each experimental condition as columns. There are a number of controls for selecting genes and conditions in the heatmap, for transforming expression level values and colorations, and for clustering values by similar expression patterns. Buttons connect the network visualization to the heatmap. For example the "Cluster" button uses the expression levels shown in the heatmap to group genes of the network with similar expression levels close together.

Any number of TRN networks and linked TRN networks can also be arranged into coordinated "multiple network" rectangular array to allow easy comparison of network models. Controls allow the researcher to coordinate the network layout of the networks, show differences between the networks, and show commonality among the networks. Researchers use the multiple network visualization to compare models derived using different inference methods, or derived from different experimental conditions. Figure 3 shows a multiple network view with two coordinated networks.

Researchers use these visualizations in Jupyter notebooks to interactively explore

data sets and later often add script fragments to the notebooks which reproduce specific configurations for the visualizations. The configuration script fragments may consist of simple assignments or they may be quite sophisticated. For example some script fragments read gene lists from external data sources and use those lists to configure networks and heatmaps. The Jupyter notebook environment supports novice level programming interactions and expert level interactions equally well, with a smooth learning curve between the two extremes.

5 Conclusion

Genomic technologies used in systems biology evolve rapidly and transcriptomic research efforts often involve many researchers at different locations with different areas of expertise, from specialists in laboratory methods, to specialists in statistical analysis, to specialists in software engineering. Repositories organized using collections of Jupyter notebooks allow research teams to share reproducible workflows specifying research methods and results. Programmable interactive visualization widgets integrated into the workflows allow researchers to explore data and data relationships and communicate observations to collaborators.

References

1. M. L. Arrieta-Ortiz, C. Hafemeister, A. R. Bate, T. Chu, A. Greenfield, B. Shuster, S. N. Barry, M. Gallitto, B. Liu, T. Kacmarczyk, et al. An experimentally supported model of the bacillus subtilis global transcriptional regulatory network. *Molecular systems biology*, 11(11):839, 2015.

2. M. Bastian, S. Heymann, and M. Jacomy. Gephi: An open source software for exploring and manipulating networks, 2009.

3. C. Blatti, M. Kazemian, S. Wolfe, M. Brodsky, and S. Sinha. Integrating motif, dna accessibility and gene expression data to build regulatory maps in an organism. *Nucleic Acids Research*, 43(8):3998, 2015.

4. R. Bonneau, D. J. Reiss, P. Shannon, M. Facciotti, L. Hood, N. S. Baliga, and V. Thorsson. The inferelator: an algorithm for learning parsimonious regulatory networks from systems-biology data sets de novo. *Genome biology*, 7(5):R36, 2006.

5. M. S. Cline, M. Smoot, E. Cerami, A. Kuchinsky, N. Landys, C. Workman, R. Christmas, I. Avila-Campilo, M. Creech, B. Gross, K. Hanspers, R. Isserlin, R. Kelley, S. Killcoyne, S. Lotia, S. Maere, J. Morris, K. Ono, V. Pavlovic, A. R. Pico, A. Vailaya, P.-L. L. Wang, A. Adler, B. R. Conklin, L. Hood, M. Kuiper, C. Sander, I. Schmulevich, B. Schwikowski, G. J. Warner, T. Ideker, and G. D. Bader. Integration of biological networks and gene expression data using Cytoscape. *Nature protocols*, 2(10):2366–2382, Sept. 2007.

6. P. D'haeseleer. What are dna sequence motifs? *Nature biotechnology*, 24(4):423–425, 2006.

7. M. Franz, C. T. Lopes, G. Huck, Y. Dong, O. Sumer, and G. D. Bader. Cytoscape. js: a graph theory library for visualisation and analysis. *Bioinformatics*, page btv557, 2015.

8. N. Gehlenborg, S. I. O'Donoghue, N. S. Baliga, A. Goesmann, M. A. Hibbs, H. Kitano, O. Kohlbacher, H. Neuweger, R. Schneider, D. Tenenbaum, and A.-C. Gavin. Visualization of omics data for systems biology. *Nat. Methods*, 7:S56–68, 2010.

9. R. C. Gentleman, V. J. Carey, D. M. Bates, B. Bolstad, M. Dettling, S. Dudoit, B. Ellis, L. Gautier, Y. Ge, J. Gentry, K. Hornik, T. Hothorn, W. Huber, S. Iacus, R. Irizarry, F. Leisch, C. Li, M. Maechler, A. J. Rossini, G. Sawitzki, C. Smith, G. Smyth, L. Tierney, J. Y. Yang, and J. Zhang. Bioconductor: open software development for computational biology and bioinformatics. *Genome biology*, 5(10):R80–16, 2004.

10. J. D. Hunter. Matplotlib: A 2d graphics environment. *Computing In Science & Engineering*, 9(3):90–95, 2007.

11. E. Jones, T. Oliphant, P. Peterson, et al. SciPy: Open source scientific tools for Python, 2001–. [Online; accessed 2017-02-22].

12. S. Kairam, N. H. Riche, S. M. Drucker, R. Fernandez, and J. Heer. Refinery: Visual exploration of large, heterogeneous networks through associative browsing. *Comput. Graph. Forum*, 34(3):301–310, 2015.

13. K. Karwacz, E. R. Miraldi, M. Pokrovskii, A. Madi, N. Yosef, I. Wortman, X. Chen, A. Watters, N. Carriero, A. Awasthi, et al. Critical role of irf1 and batf in forming chromatin landscape during type 1 regulatory cell differentiation. *Nature Immunology*, 2017.

14. J. Kuan. *Learning Highcharts*. Community experience distilled. Packt Pub., 2012.

15. D. Merkel. Docker: Lightweight linux containers for consistent development and deployment. *Linux J.*, 2014(239), Mar. 2014.

16. A. Osheroff. binder. `https://github.com/binder-project/binder`, 2017.

17. C. Partl, A. Lex, M. Streit, D. Kalkofen, K. Kashofer, and D. Schmalstieg. enroute: dynamic path extraction from biological pathway maps for exploring heterogeneous experimental datasets. *BMC Bioinformatics*, 14(19):S3, 2013.

18. F. Pedregosa, G. Varoquaux, A. Gramfort, V. Michel, B. Thirion, O. Grisel, M. Blondel, P. Prettenhofer, R. Weiss, V. Dubourg, J. Vanderplas, A. Passos, D. Cournapeau, M. Brucher, M. Perrot, and E. Duchesnay. Scikit-learn: Machine learning in Python. *Journal of Machine Learning Research*, 12:2825–2830, 2011.

19. F. Pérez and B. E. Granger. Ipython: a system for interactive scientific computing. *Computing in Science & Engineering*, 9(3), 2007.

20. J. A. Reuter, D. V. Spacek, and M. P. Snyder. High-throughput sequencing technologies. *Molecular cell*, 58(4):586–597, 2015.

21. Y. Wang, T. Joshi, X.-S. Zhang, D. Xu, and L. Chen. Inferring gene regulatory networks from multiple microarray datasets. *Bioinformatics*, 22(19):2413–2420, 2006.

22. A. Watters, , and E. Miraldi. https://github.com/simonsfoundation/jp_gene_viz. `https://github.com/simonsfoundation/jp_gene_viz`, 2017.

23. N. Yosef, A. K. Shalek, J. T. Gaublomme, H. Jin, Y. Lee, A. Awasthi, C. Wu, K. Karwacz, S. Xiao, M. Jorgolli, et al. Dynamic regulatory network controlling th17 cell differentiation. *Nature*, 496(7446):461–468, 2013.

24. B. Yu, H. Doraiswamy, X. Chen, E. Miraldi, M. L. Arrieta-Ortiz, C. Hafemeister, A. Madar, R. Bonneau, and C. T. Silva. Genotet: An interactive web-based visual exploration framework to support validation of gene regulatory networks. *IEEE transactions on visualization and computer graphics*, 20(12):1903–1912, 2014.

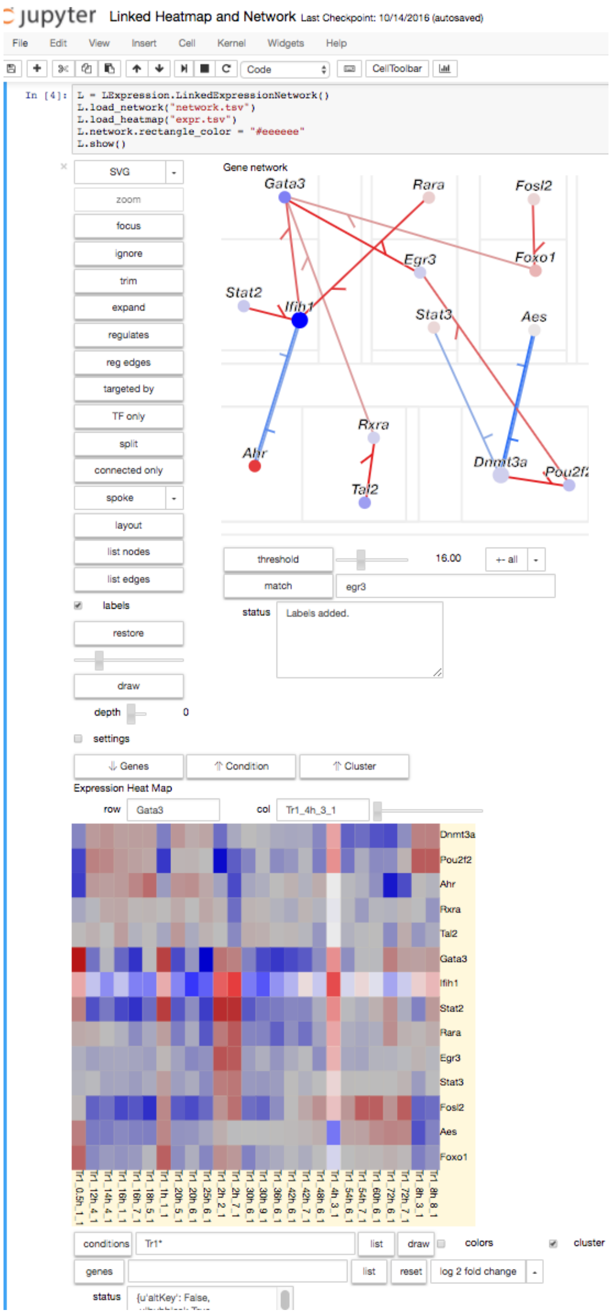

Figure 1. An interactive heatmap of gene expression data (below) linked to a TRN network model visualization (above). Genes of the network have been clustered by similar expression pattern in the heatmap and are restricted to transcription factors related to the EGR3 gene. Expression levels in the heatmap are normalized by log 2 fold change and are restricted to the genes of the network under the experimental conditions matching "Tr1*". Nodes of the network are colorized by expression levels in the experimental condition "Tr1_4h_3_1".

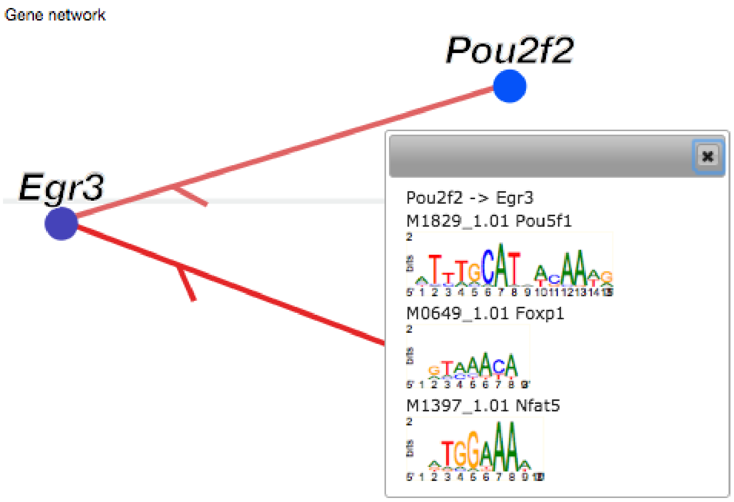

Figure 2. A network annotated with sequence motifs displays the motifs associated with a transcriptional relationship on mousing over the edge for the association.

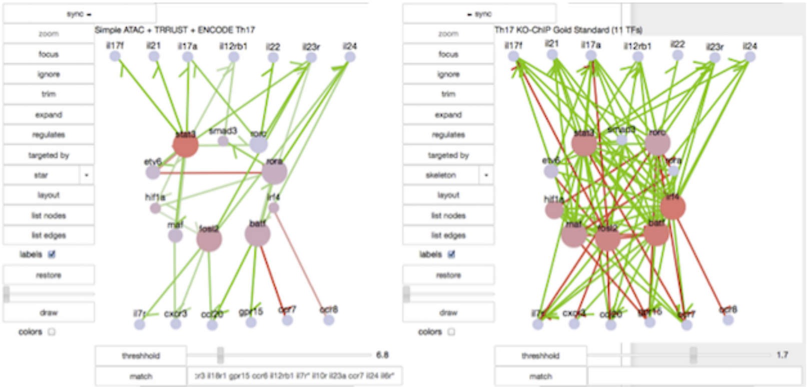

Figure 3. A multiple network display comparing a "core" TRN (right) with an inferred TRN (left). The multiple network view coordinates any number of networks arranged in a rectangular array.
