# Supplementary figures and images for: Leveraging chromatin accessibility for transcriptional regulatory network inference in T Helper 17 Cells
Source: Genome Res. 2019 Mar;29(3):449–63. doi: 10.1101/gr.238253.118 (PMC6396413; doi:10.1101/gr.238253.118)

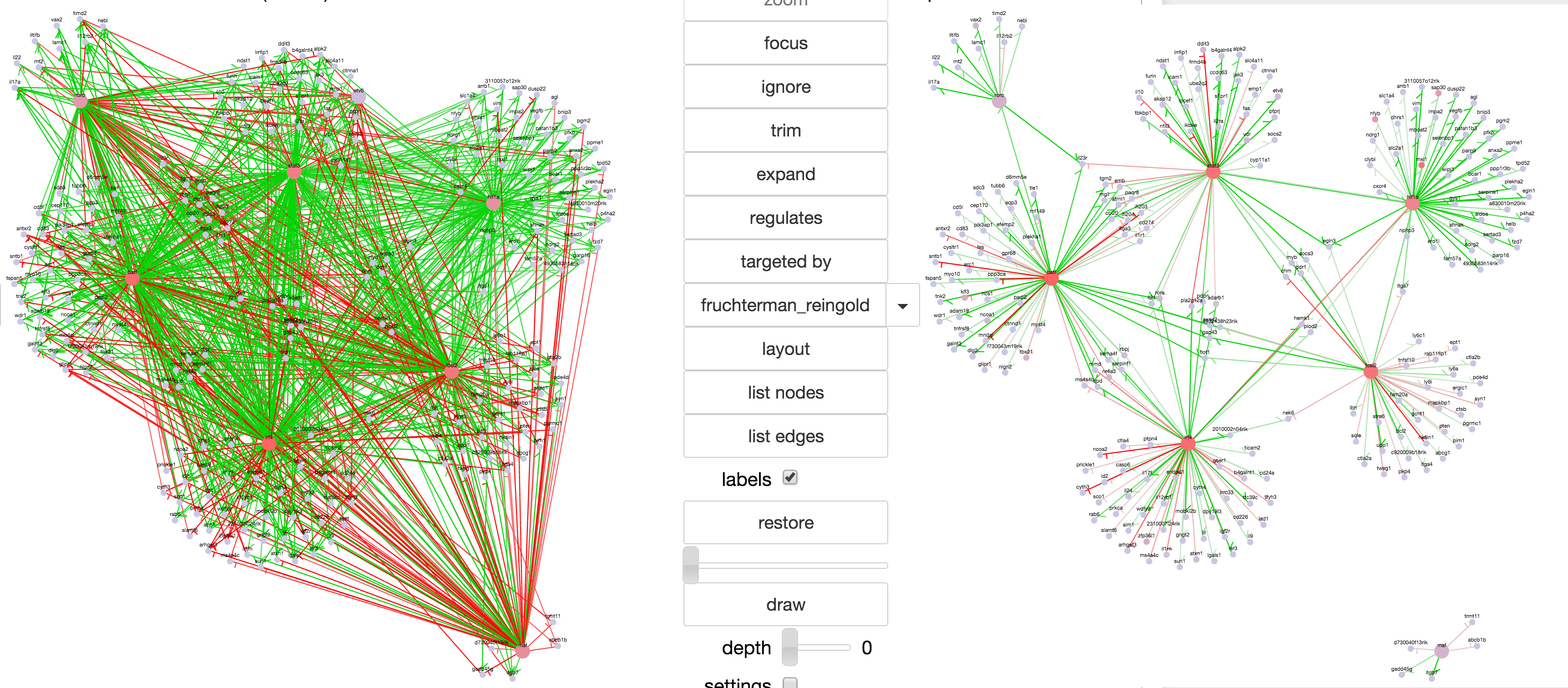

Supplement: Supplemental Material [file supp_gr.238253.118_Supplemental_jp_gene_viz.zip › jp_gene_viz-master/bioRxiv/gold.png]

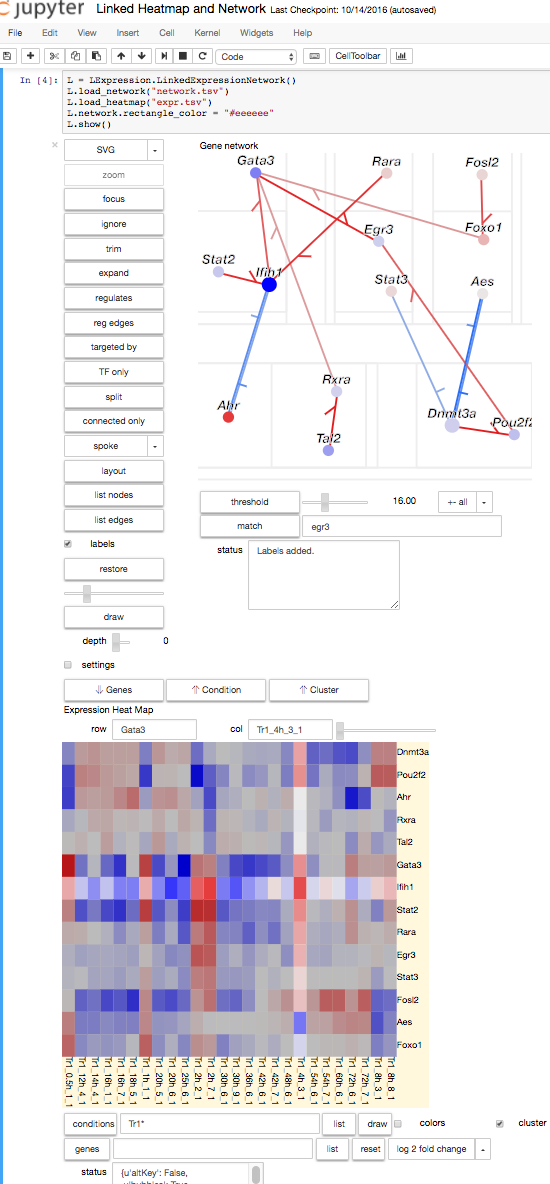

Supplement: Supplemental Material [file supp_gr.238253.118_Supplemental_jp_gene_viz.zip › jp_gene_viz-master/bioRxiv/Linked.png]

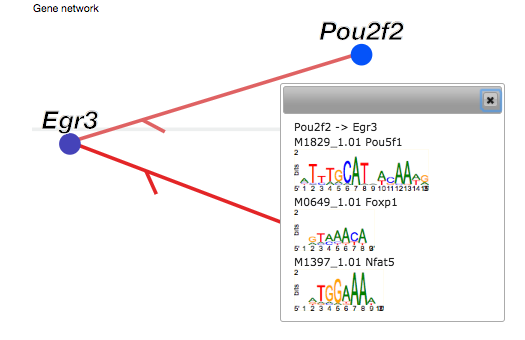

Supplement: Supplemental Material [file supp_gr.238253.118_Supplemental_jp_gene_viz.zip › jp_gene_viz-master/bioRxiv/motif.png]

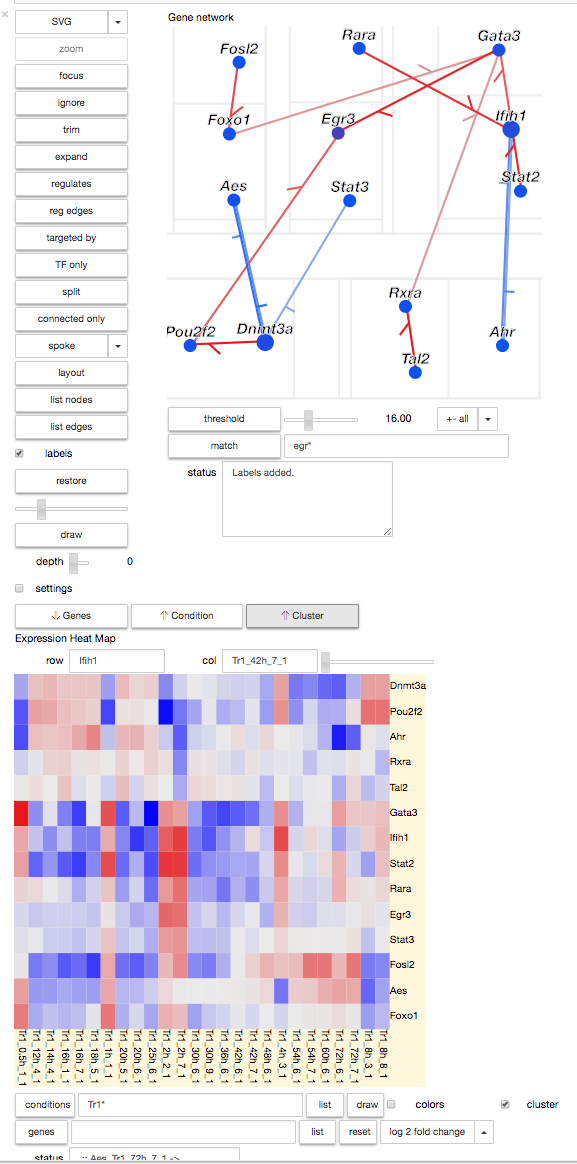

Supplement: Supplemental Material [file supp_gr.238253.118_Supplemental_jp_gene_viz.zip › jp_gene_viz-master/bioRxiv/old_linked.png]

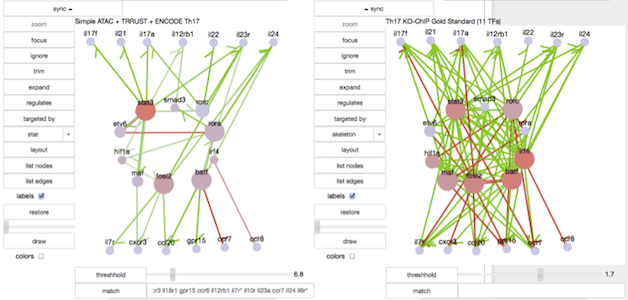

Supplement: Supplemental Material [file supp_gr.238253.118_Supplemental_jp_gene_viz.zip › jp_gene_viz-master/bioRxiv/pair.png]

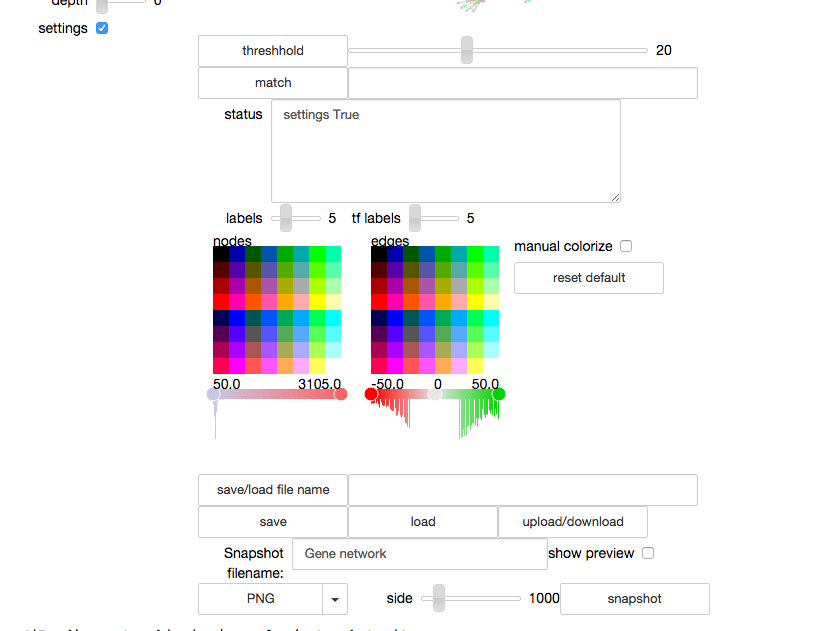

Supplement: Supplemental Material [file supp_gr.238253.118_Supplemental_jp_gene_viz.zip › jp_gene_viz-master/doc/dNetwork_settings.png]

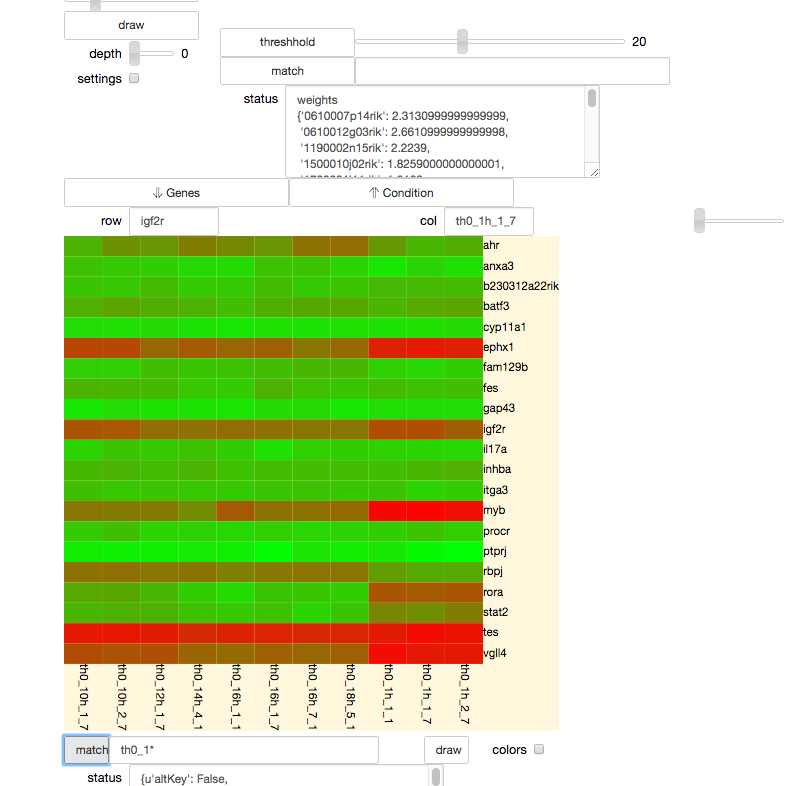

Supplement: Supplemental Material [file supp_gr.238253.118_Supplemental_jp_gene_viz.zip › jp_gene_viz-master/doc/heat_map_widget.png]

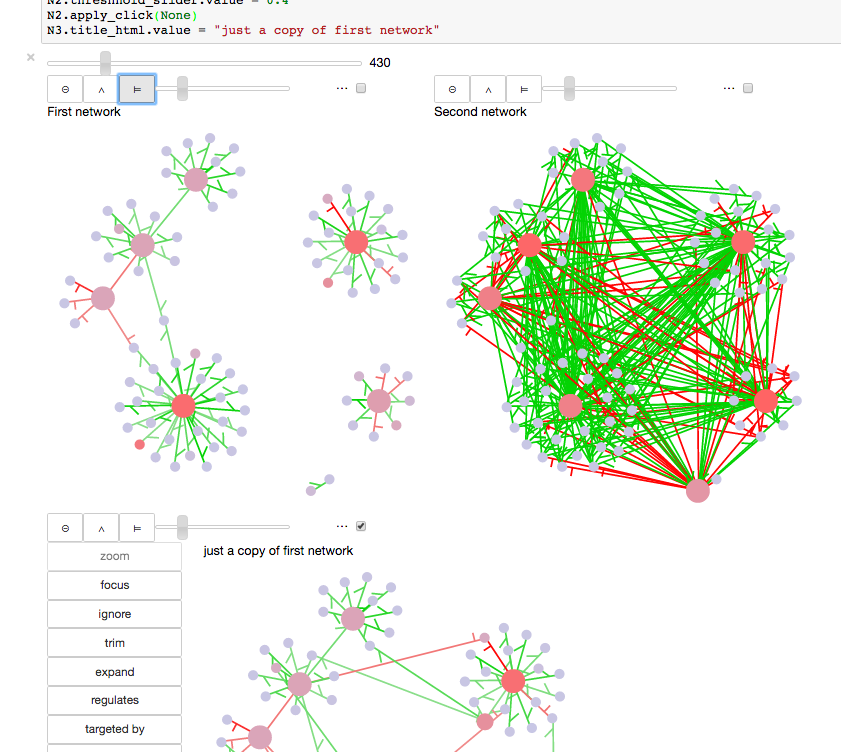

Supplement: Supplemental Material [file supp_gr.238253.118_Supplemental_jp_gene_viz.zip › jp_gene_viz-master/doc/multiple_network.png]

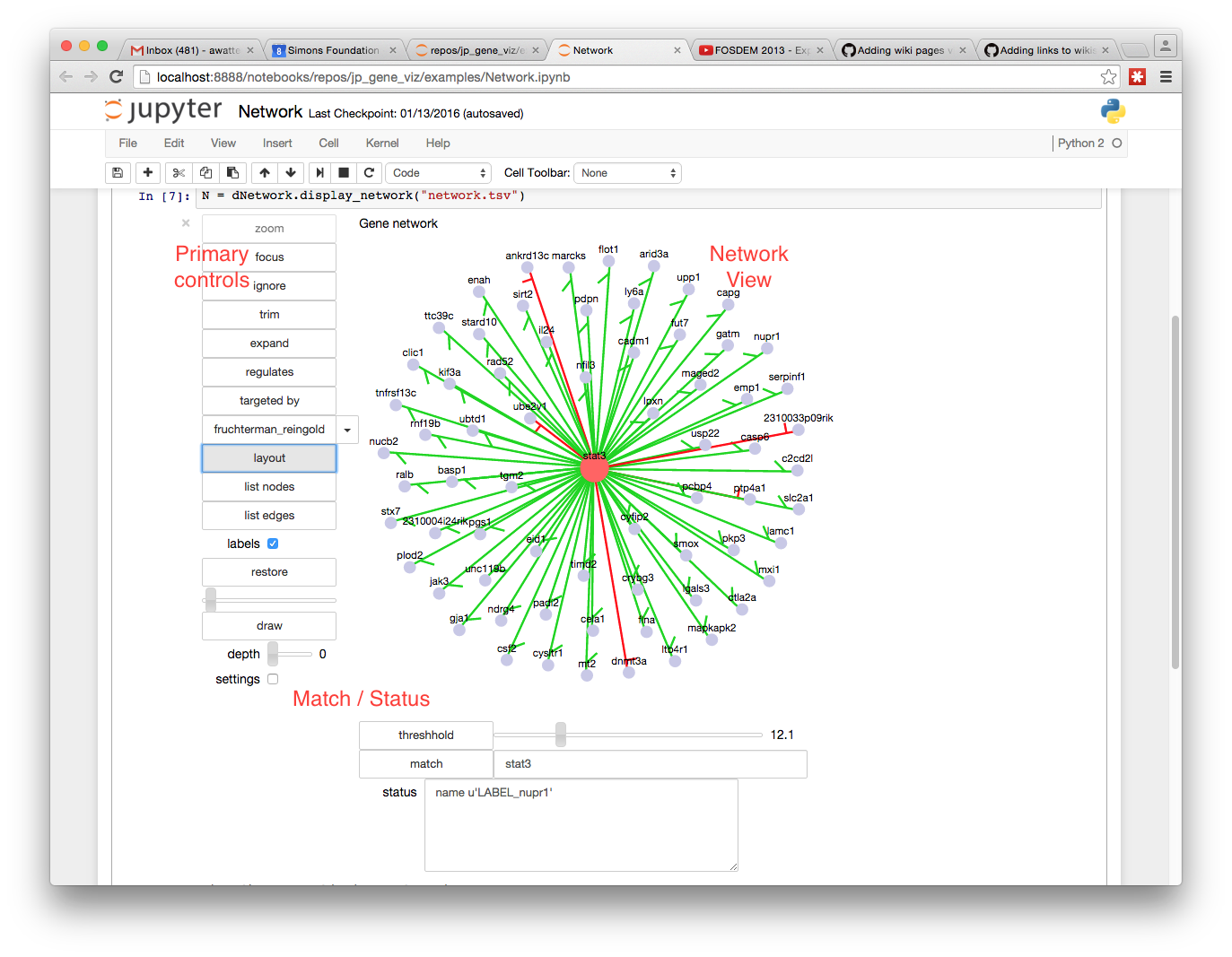

Supplement: Supplemental Material [file supp_gr.238253.118_Supplemental_jp_gene_viz.zip › jp_gene_viz-master/doc/network.png]
