# Supplementary material for: Leveraging chromatin accessibility for transcriptional regulatory network inference in T Helper 17 Cells
Source: Genome Res. 2019 Mar;29(3):449–63. doi: 10.1101/gr.238253.118 (PMC6396413; doi:10.1101/gr.238253.118)
Supplement: Supplemental Material [file supp_gr.238253.118_Supplemental_Materials.docx]

**Supplemental Materials**

**Table of Contents**

| Supplemental Notes | Page 1 |
| --- | --- |
| Supplemental Figure Legends | Page 6 |
| Supplemental Table Legends | Page 11 |
| Supplemental Codebases | Page 11 |
| Supplemental References | Page 11 |
|  |  |

**Supplemental Notes**

**Supplemental Note 1. Source of prior, TFA method and prior reinforcement dramatically alter Th17 TRNs.** mLASSO-StARS outperformed BBSR-BIC in terms of precision-recall. Yet the effect of other modeling decisions (choice of prior, TFA method, etc.) is less dramatic. Precision-recall analysis, limited to the 9-25 TFs in the gold standards, revealed some differences among the mLASSO-StARS models, while all models had roughly similar out-of-sample gene-expression prediction. Having performed these initial quality assessments, we explore the Th17 TRNs globally.

Standardizing network sizes to 53k TF-gene interactions (~15 TFs/gene), we calculated the percent edge overlap among TRNs built from ATAC, ChIP, KO, ENCODE DHS or TRRUST priors as well as two combined priors, expected to be improvements relative to their individual constituents. The first combined prior “ChIP/A(Th17)” uses TF ChIP data when available and TF motif analysis of 48h Th17 ATAC-seq data otherwise. The second prior “ChIP/A(Th17)+KO+A(Th)” combines the first with KO and ATAC-seq data from the other time points and conditions (**Methods**). For each prior, we considered five modeling modes: prior-based TFA estimates (“TFA”) with no, moderate or strong prior reinforcement and TF mRNA TFA estimates (“m”) with moderate and strong prior reinforcement. We also included the No Prior TRN

as a control. The results described below, for network sizes ~15 TFs/gene, hold for other network-size cutoffs (5, 10, and 20 TFs/gene, **Supplemental Fig. S13**). The percentage of shared edges between TRNs ranged from 83% to 10%. Clustering revealed several trends (**Supplemental Fig. S8A**). Over a third of the TRNs cluster together with high overlaps (60-88%), highlighted by a green-outlined box in **Supplemental Fig. S8A**. We refer to this cluster as “gene-expression-driven”, because it contains the No Prior TRN and other TRNs where TF-gene interactions are mainly based on TF-mRNA TFA estimates. The cluster contains all TF-mRNA models for the smaller priors (ChIP, KO, TRRUST) as well as some models built with prior-based TFA, but only for priors with few TFs (ChIP and KO) and for which TF mRNA must be used for the remaining TFs. Consistent with this explanation, the TRRUST TRNs built with prior-based TFA cluster separately. Although the TRRUST prior has fewer edges (~1500) than either the ChIP (~13k) or KO (~8k) prior, its edges are distributed among 187 of the 715 potential TF regulators considered (**Table S1B**), and, thus, TRRUST-based TFA greatly alters the resulting TRN.

Three priors contain ATAC-seq data (ATAC, ChIP/A(Th17), and ChIP+KO+ATAC); these priors are large (ranging from ~120-200k interactions for 346 TFs), so large that not even moderately reinforced TF-mRNA TRNs cluster with the “gene-expression driven” TRNs. The ATAC-containing TRNs form two clusters (marked by light-blue boxes in **Supplemental Fig. S8A**) that separate based on TFA estimation method. As expected, the “ATAC TF mRNA” cluster is more coherent (overlaps ranging from 47-87%), while overlaps in the “ATAC prior-based TFA” cluster range from 17-70%. The ATAC TRNs built on TF mRNA are most similar to the “gene-expression driven cluster”, but with only ~50-65% edge overlap for moderate-strength prior reinforcement. At high prior reinforcement, overlap drops to ~35-45%. The “ATAC prior-based TFA” cluster is as dissimilar to the “ATAC TF mRNA” cluster as it is to the “gene-expression driven cluster” (overlaps with both clusters ranging from 10-30%). Thus, as also suggested by the TRRUST cluster, prior-based TFA involving a large number of TFs, dramatically changes the resulting TRN. The ENCODE prior produced the most dissimilar TRNs, both when considering ENCODE TRNs relative (1) to each other and (2) to TRNs based on other priors. This result makes sense, given the unique origin and large size of the ENCODE prior (~200k interactions for 238 TFs).

To further our intuition about these distinct TRNs, we visualized the percentages of genes regulated by TFs with high degree across TRNs (**Supplemental Fig. S8B**). The high-degree TFs can be roughly divided into four clusters. Some top-degree TFs are mainly driven by gene expression (ERG, FOXB1, IKZF2, denoted by an “m” in **Supplemental Fig. S8B**) and regulate ~7-9% of the 3578 network genes not only in the “gene-expression TRNs” cluster but also TF mRNA-based ENCODE and ATAC-containing networks. Moderate to strong reinforcement of ChIP and KO priors lead to high degree for key Th17 TFs: BATF, IRF4, and STAT3. In the No Prior TRN, the percent of genes regulated by these TFs hovers between 4-10%, while, for strongly enforced ChIP TRNs, the percent genes regulated climbs to 17-40%. Strongly reinforced priors composed of ChIP data in combination with ATAC and/or KO data also leads to high degree for this TF cluster, ranging from control of 15-32% of genes. Priors with few TFs (e.g., KO and ChIP) lead to models that contain >75% edge overlap with the No Prior TRN across models, but this high overlap can be deceptive. Even for small priors, the modeling decisions can significantly alter the TF degree distribution.

The differences in network overlap and high-degree TFs between methods might reflect different strengths of each method and prior information source. Although overall-network performance in terms of precision-recall is roughly similar between methods and priors, the TF-specific AUPRs were heterogeneous by method (**Figure 2C**). Thus, each method enriched for unique sets of TF-specific gold-standard interactions among its high-confidence edges, and the ensemble of predictions from each model and method might provide a fuller picture of the underlying TRN. This could be an important avenue for future work.

**Supplemental Note 2. Treatment of RNA-seq batch effects.** The final gene expression matrix contained samples generated over an eight-year period, from several genomics cores, and with a combination of 36-bp and 50-bp single-end reads as well as 50-bp paired-end reads. There was no common set of control samples across batches. We first attempted to control for differences in sequencing, mapping only a single-end from paired-end sequencing samples with STAR aligner (Dobin et al. 2013). In addition, we controlled for differences in mappability between 36-bp and 50-bp reads by calculating effective gene lengths. Specifically, we calculated the number of possible 36-mers or 50-mers that HTSeq-count would map per gene (using HTSeq-count parameters: --stranded=no --mode=union and 36-mer and 50-mer mappability tracks (Derrien et al. 2012)). We then computed a factor (effective 36-mer gene length to effective 50-mer gene length) to convert 50-mer gene quantification by HTSeq-count to 36-mer gene quantification scale. From there, we used DESeq2 (Love et al. 2014) to normalize data. We used PCA to ensure that major trends did not correlate with (1) sequencing, (2) date of experiment, or (3) library size. Instead, they correlated well with the T Cell time course and polarization conditions (**Fig. 1A, S3**).

**Supplemental Note 3. Prior Construction details.** (Note that **Methods** detail ATAC-seq prior generation.)

*ChIP-seq*: TF ChIP-seq and control sequencing data were downloaded from GEO (GSE40918), mapped to the murine genome (mm10) with bowtie2 (2.2.3), filtered based on mapping score (MAPQ > 30, Samtools (0.1.19)), and duplicates removed (Picard). Peaks were called with macS12 version 1.4.2 (parameters: -m 10,30 -g 1865500000 --bw=200) and retained for raw p-value < 10^-10^. TFs were associated with a target gene, if the ChIP peak fell within +/-10kb of gene body.

*ENCODE DHS*: TF-gene interactions, based on TF-footprinting analysis in ENCODE DNase1-hypersensitivity (DHS) in 25 mouse tissues (Stergachis et al. 2014) were downloaded from <http://www.regulatorynetworks.org>.

*TRRUST*: Signed, human TF-gene interactions were downloaded from the Transcriptional Regulatory Relationships Unraveled by Sentence-base Text-mining (TRRUST) database (Han et al. 2015), version 1, and mapped to mouse orthologs using the MGI database (one-to-many mappings included).

*ChIP/A(Th17)*: This prior uses TF ChIP data when available and TF motif analysis of 48h Th17 ATAC-seq data otherwise.

*ChIP/A(Th17)+KO+A(Th)*: This prior represents a combination of ChIP/A(Th17) prior, the 25 TF KO experiments in the G.S., and A(Th) prior. Each prior matrix was Frobenius-norm normalized and multiplied by the number of TFs, so that the predictions of each TF per prior had comparable weight. The interactions in the normalized priors were then summed, and the sign of prior interaction was determined from KO data, when available.

Given the degeneracy of TF motifs, some TFs had identical target gene sets within ATAC-seq and ENCODE DHS priors. Degenerate TFs were merged for TRN inference. For precision-recall analysis, resulting target edges for merged TFs were included for all degenerate TFs in the set.

**Supplemental Note 4. Consideration of time-series samples***.* Our gene expression matrix includes a small fraction of time-series samples (15 out of 254), and so we tested whether linear differential equations, as proposed in the original *Inferelator* algorithm (Bonneau et al. 2006), would improve inference over the steady-state assumption (**Equation 1**) for our particular experimental design. For prior-based TFA (in contrast to TF mRNA), there is no obvious biological motivation for time-lag. However, given that TF mRNA is used to estimate the activities of TFs without prior information (~60% of TFs in our study), the steady-state assumption could also affect prior-based TFA. Using the time-lag parameter tau (30 minutes) from (Ciofani et al. 2012), we compared performance between TRNs built with and without time-lag. Precision-recall of the KO+ChIP and KO G.S.’s did not change between time-lagged and steady-state models (**Supplemental Fig. S23**). However, we do recommend linear modeling of differential gene expression for study designs involving more time-series data, and we provide workflows with this option in our codebase (https://github.com/emiraldi/infTRN_lassoStARS.git).

**Supplemental Note 5. Evaluating StARS for TRN inference.** We tested two ways of calculating average instability: for each gene model individually (“per-gene”) and for all gene models at a given $\lambda$ (“network”). We reasoned that “per-gene” estimates might lead to better models for each gene, but the “network” average instability estimates would be more stable, requiring less search time for the $\lambda$ corresponding to the desired instability cutoff (**Supplemental Fig. S24**). We visualized the distribution of subsampled TF-gene edge frequencies for per-gene and network average instability estimates at cutoffs = .05, .1 and .2 (**Supplemental Fig. S25**). Typically, the edges selected for the final network are those that are stably nonzero (i.e., present), with an instability less than or equal to the cutoff. For both per-gene and network average instability estimates, we noted that the average network size ranged from ~3.5 TFs/gene to ~9 TFs/gene for the default cutoff .05 to .2. Thus, conventional filtering of edges with StARS would lead to very sparse models, especially using the recommended .05 cutoff.

As we had done previously with application of StARS to ecological network inference (Kurtz et al. 2015), interactions were ranked according to nonzero subsamples per edge. However, this led to a plateau at the beginning of the precision-recall curve (at high precision, low recall) for 50 subsamples (**Supplemental Fig. S26A**), and the plateau was not much improved by more time-consuming calculations involving 100 or 200 subsamples. Thus, we proposed:

Confidence(i,k) = Nonzero Subsamples + |*pcorr*(i,k)|, [**Equation 4**]

where i and k, again correspond to gene i and TF k, and *pcorr*(i,k) is the partial correlation between gene i and TF k, for a set model size. For TF-gene interactions with the same number of nonzero subsamples, the TF-gene interaction with high absolute partial correlation will be higher confidence.

**Equation 4** had the desired effect, improving precision-recall for both KO+ChIP (**Supplemental Fig. S26, S27**) and KO (**Supplemental Fig. S28**) gold standards (for both network- and gene-level average instabilities over a range of cutoffs: .05, .1 and .2). We also tested the same sets of conditions for all three out-of-sample prediction leave-out sets; representative results for the Early Th17 conditions are shown in **Supplemental Fig. S29**. Overall, precision-recall and out-of-sample prediction were robust to choice of network- versus per-gene average instability cutoff (**Supplemental Fig. S26-S28**). Out-of-sample prediction was consistently better using the instability cutoff .05 versus .1 and .2 (**Supplemental Fig. S29**). Thus, we chose the cutoff of .05, additionally noting reduced computation time that (.05 corresponds to a larger $\lambda$). Given that network-level average instability performed on-par with per-gene and was computationally less expensive, we used network-level estimates for all subsequence analyses.

Although we relied on the instability cutoff of .05 to select $\lambda$ and calculate edge confidences, we did not use the instability cutoff to select which edges to include in the final network, as is typically done using StARS. Instead, we recommend using quality metrics (precision, recall and out-of-sample prediction) to guide in selecting the final network size. Small differences in the cutoff (.05 to .2) led to large differences in network size (**Supplemental Fig. S25**), while network quality-metric curves (especially precision-recall (**Supplemental Fig. S27, S28**)), based only on ranking of TF-gene interactions (**Equation 4**), did not change much. Applying the StARS cutoff to select the network size (represented by circles and x’s on curves in **Supplemental Fig. S27-S29**) is likely overly conservative. For example, applying cutoff = .05 yields a model size averaging 3 TFs/gene and corresponds to a median per-gene R^2^_pred_ value that is significantly lower than that of larger models (e.g., ~2-fold lower than median per-gene R^2^_pred_ at 15 TFs/gene, **Supplemental Fig. S29B**), suggesting that using cutoff = .05 to select model size would omit TF-gene interactions with predictive value from the model. Based on these analyses, we recommend using the above quality metrics to guide selection of model-size cutoff.

**Supplemental Note 6. mLASSO-StARS speed-ups using bStARS.** To expedite instability calculations, we use average instability upper and lower bounds from bStARS (Müller et al. 2016). Using two subsamples to calculate upper and lower bounds and 50 subsamples for final instability estimates, the speed up from bStARS was negligible, while lower bounds estimated from five subsamples were tighter (**Supplemental Fig. S30**) and computation time was decreased > two-fold (from 75 to 35 minutes using a single core on a 2017 MacBook Pro with 2.9 GHz Intel Core i7 processor).

**Supplemental Note 7. Potential overfitting in gene-expression prediction using prior-based TFA.** For gene-expression prediction with prior-based TFA, the mRNA of target genes with edges in the prior contribute to TFA estimation (**Equation 2**), and then their gene expression patterns are predicted as a function of TFA. This circularity could lead to over-fitting and inflated $R_{pred}^{2}$ values. To test this possibility, we constructed a prior-based TFA, A^-i^*^i^*, for each gene *i*, by solving:

X^-i^=P^-i^A^-i^, [**Equation 6**]

where matrices X^-i^, and P^-i^ omit the rows corresponding to target gene i. Given the large gene-dimension of the prior (e.g., 17,138 genes for the ATAC (Th17) prior), we anticipated that A^-i^ ~ A. To test this assumption, we regenerated results for prior-based TFA (Th17 ATAC prior, bias = .5) according to [**Equation 6**]. There were 2803 target genes (out of 3578) with edges in the ATAC prior. For those TFs k that lost an edge to gene i when calculating A^-i^, we calculated the Pearson correlation between A^-i^(k) and A(k) and plotted the cumulative distribution function (CDF) for the 108,850 resulting correlations (**Supplemental Fig. S31**). 99% of the TF activities were correlated with rho > .99, while a handful of correlations dipped below rho .95 (15 correlations (1.4%)). Thus, A^-i^ ~ A. We repeated leave-out gene expression prediction used gene-specific A^-i^, and, for all three expression prediction challenges, results were similar, even when limiting to the set of target genes with edges in the prior (**Supplemental Fig. S32**). This analysis confirms that our $R_{pred}^{2}$ estimates were robust to our decision to approximate A^-i^ ~ A.

**Supplemental Note 8. Performance of max- and rank-combine to individual TRNs and combination of TRNs derived from different priors.** Given the complementary performance of TF-mRNA- and prior-based TFA, we sought to combine the TRNs using both TFA methods (at moderate prior reinforcement). Given, their unique TF-specific AUPR profile (**Fig. 2C**), we combined methods by taking the maximum to preserve the individual strengths of each (Kittler et al. 1996; Castro et al. 2018). We compare and compared to rank-sum (or average) combination (Marbach et al. 2012), which would emphasize commonalities but potentially mask individual strengths. As anticipated, max-rank combination had better precision-recall than rank-sum combination (**Supplemental Fig. S33**). For combination of edges with different sign, partial correlations were averaged to yield sign in the combined TRN. Given the orthogonal nature of the TRRUST prior (publicly available to those considering experimental designs for TRN inference), we also tested rank-combination of ATAC and TRRUST TRNs (combining a total of four TRNs, TF mRNA and prior-based TFA for each prior), but observed no benefit, in terms of improved precision-recall (**Supplemental Fig. S34**). More sophisticated methods for integrating TRNs would likely show benefit over the simple techniques tested here (Parisi et al. 2014; Castro et al. 2018), but are beyond the scope of this work.

**Supplemental Note 9. TF-TF Module Analysis.** We calculated the number of shared target genes between each pair of TFs, analyzing positive and negative target edges separately. (Edges with |partial correlation| < .01 were excluded from analysis, as were TFs with fewer than 20 gene targets.) TFs vary greatly by number of target genes (**Supplemental Fig. S10, S11**), so we devised an overlap normalization scheme that controlled for the variable number of targets per TF. This approach is detailed in **Supplemental Note 9**. Specifically, we took inspiration from context likelihood of relatedness (CLR), a background-normalized mutual information score (Faith et al. 2007). We define the background-normalized overlap score $z_{ij}$ between TF *i* and TF *j* as:

$z_{ij}=\left\{ \begin{matrix} \sqrt{{z_{i|j}}^{2}+{z_{j|i}}^{2}}, & if z_{i|j}{, z}_{j|i}>0 \\ 0, & otherwise \end{matrix} \right.$, [**Equation 7**]

where $z_{i|j}$ is the z-score of the overlap between TF *i* and TF *j*, using the mean and standard deviation associated with the overlaps of TF *j* to calculate the z-score. Note that the score is only nonzero if the overlap is above average for both TFs. The normalized overlap score has good agreement with overlap significances estimated using the hypergeometric CDF.

We filtered the normalized overlap matrix so that it contained only TFs with at least one significant overlap (FDR = 10%, hypergeometric CDF). We then converted the similarity matrix of normalized overlaps to a distance matrix for hierarchical clustering using Ward distance. To arrive at a final number of clusters, we calculated the mean silhouette score for solutions over a range of total clusters and selected the solution that maximized mean silhouette score. For positive interactions (149 or 167 TFs), this analysis lead to 42 clusters, and 9 or 11 clusters for negative interactions (31 or 30 TFs), for final ChIP+KO+ATAC or ATAC-only TRNs, respectively.

To prioritize and rank TF-TF clusters, we developed a method based on combination of p-values generated from the empirical distribution of normalized overlap scores. For each cluster, we combined p-values for the pairwise normalized overlap scores between cluster TFs (e.g., for cluster of size $s$, there were $s(s-1)/2$ pairwise scores). We combined p-values with the weighted z-method (Whitlock 2005). P-values are converted via the inverse normal CDF to z-score space and combined with the following equation:

$Z_{w}= \frac{\sum_{i=1}^{n} {w_{i}Z}_{i}}{\sqrt{\sum_{i=1}^{n} w_{i}}}$, [**Equation 8**]

where $n$ is the number of p-values to be combined and $w\in\mathbb{R}^{n}$ is a vector of weights. $Z_{w}$ is then converted to a p-value using the normal CDF. Here we set weights to $2/(s-1)$. The resulting p-value score had the desired properties, ranking clusters on the basis of size and strength of overlap (**Supplemental Fig. S35A**) and was used to prioritize a limited set of 15 positive-edge TF-TF clusters for **Fig. 5C, S14, S15**. The significance of negative TF-TF clusters were orders of magnitude smaller than the top-15 positive TF-TF clusters (**Supplemental Fig. S35B**), so were not analyzed further. The lack of significant negative TF-TF clusters might have to do with the observed positive to negative edge bias in our method (~1.8-1.9:1).

**Supplemental Figure Legends**

**Supplemental Fig. S1. Clustering of all 63,049 peaks in the ATAC-seq dataset**. ATAC-seq peak intensities were robustly normalized (DESeq2), z-scored and clustered using K-means clustering with Euclidean distance. Samples were ordered according to treatment condition and timepoint.

**Supplemental Fig. S2.** **PCA of Gene Expression Profiles.** Upper panels show scores plots, where the 254 RNA-seq samples are plotted as a function of all genes for principal components (PCs) 1-4. Lower panels display gene loadings for the top-75 gene contributors to PCs 1-4.

**Supplemental Fig. S3. Precision-recall of multiple priors, Gold Standard = KO+ChIP (A) or KO (B).** For each prior, the performance is plotted for several TRNs, based on *Inferelator* method (LS = mLASSO-StARS (reds), BB = BBSR-BIC (blues)), TFA estimation method (m = TF mRNA, TFA = P^+^X), and strength of prior reinforcement (none, moderate (+), and strong (++)). Random and “No Prior” control TRNs serve as references in all panels.

**Supplemental Fig. S4. Precision-recall of multiple priors, G.S. = KO+ChIP (A) or KO (B), effect of RNA-seq dataset size.**  TRNs were built from 50 RNA-seq samples (rather than the full 254). For each prior, the performance is plotted for several TRNs, based on *Inferelator* method (LS = mLASSO-StARS (reds), BB = BBSR-BIC (blues)), TFA estimation method (m = TF mRNA, TFA = P^+^X), and strength of prior reinforcement (none, moderate (+), and strong (++)). Random and “No Prior” control TRNs serve as references in all panels.

**Supplemental Fig. S5. TF-specific TRN performance for mLASSO-StARS and BBSR-BIC. (A)** For each G.S., AUPRs were calculated for each TF individually. TF-specific performance of TRNs is quantified as the log_2_-foldchange between AUPR of the TRN model relative to random. “+” indicates strength of prior reinforcement, and “m” and “TFA” denote TFA estimation from TF mRNA and prior-based TFA, respectively. **(B)** Boxplots of per-TF AUPRs for mLASSO-StARS models in **(A);** central mark indicates median, box edges indicate the 25^th^ and 75^th^ percentiles, and the whiskers extend to the most extreme data points not considered outliers (+/-2.7 standard deviations from the mean).

**Supplemental Fig. S6. Distributions of per-gene R^2^_pred_ values.** Full and zoomed-in boxplots of per-gene R^2^_pred_ values for the Th17 ATAC prior, bias = .5 and TFA = TF mRNA or prior-based TFA (TFA = P^+^X). The central red mark indicates median, the bottom and top edges of the blue box indicate the 25^th^ and 75^th^ percentiles, and the whiskers extend to the most extreme data points not considered outliers (+/-2.7 standard deviations from the mean). x-axis indicates the average number of TFs per gene at each model-size cutoff.

**Supplemental Fig. S7. Out-of-sample prediction of gene expression patterns for TFA = TF mRNA and No Prior TRNs.** Leave-out sets are defined as in **Figure 4A**. Model selection and parameter estimation of TF-gene interactions were performed in the absence of specified leave-out sets (All Th0, Late Th17, and Early Th17). TRN models were built without prior (left panels), with an ATAC (central panels) or ChIP+KO+ATAC priors (right panels), using mLASSO-StARS, bias = .5, and TFA = TF mRNA (upper panels) TFA = P^+^X (lower panels). R^2^_pred_ for each leave-out set is plotted as a function of average number of TFs per gene. In the key, the number of samples per leave-out set appears in parentheses. For reference, precision and recall of the full model are also plotted, providing intuition about the number of KO or KO+ChIP edges in the TRN at each cut off.

**Supplemental Fig. S8. (A) Edge overlaps of TRNs.** TRNs were built with the Th17 ATAC, ChIP, KO, ENCODE DHS, TRRUST, ChIP/ATAC or ChIP+ATAC+KO priors, with TFA based on target genes (“TFA”) or TF mRNA (“m”) and varying strengths of prior reinforcement (+ = moderate, ++ = strong, or none). The “No Prior” TRN was included as a control. Each TRN model size was limited to a mean of 15 TFs per gene and hierarchically clustered using 1-overlap distance and ward linkage. **(B) Network coverage by high-degree TFs.** A TF was included in the “high-degree” TF set, if it was within the top-two highest degree nodes in one or more of the TRNs. The percentage of genes regulated by TF is based on the 3578 target genes.

**Supplemental Fig. S9. Additional Th17 core TRN models.** “Core” Th17 genes and TFs were selected from the literature for visual comparison with jp_gene_viz software. Network size was limited to an average of 15 TFs per gene. Source of network and TFA method is denoted for each network. “+” denote moderate prior reinforcement with bias = .5. The edges in Inferelator TRNs are colored according to partial correlation (red positive, blue negative) and weighted relative to edge stability. Solid edges had support in the prior, while dotted edges were learned from gene expression modeling alone. Nodes are colored according to z-scored gene expression at 48h in Th17 relative to the other T Helper cell time points (red/blue = increased/decreased expression).

**Supplemental Fig. S10. Distribution of targets per TF in final ChIP+KO+ATAC TRN.** Network size was limited to mean 15 TFs per gene and network edges were further filter to remove any edge with absolute partial correlation < .01. TFs were ranked according to degree (total number of target genes) in the left panel, while the inset displays the Top 100 highest-degree TFs. TF target genes are colored according to interaction sign and whether the interaction was also in the prior (see key).

**Supplemental Fig. S11. Distribution of targets per TF in final ATAC TRN.** Network size was limited to mean 15 TFs per gene and network edges were further filter to remove any edge with absolute partial correlation < .01. TFs were ranked according to degree (total number of target genes) in the left panel, while the inset displays the Top 100 highest-degree TFs. TF target genes are colored according to interaction sign and whether the interaction was also in the prior (see key).

**Supplemental Fig. S12. De Novo Th17 Core TFs of the ChIP+KO+ATAC and ATAC-only TRNs derived from different model-size cutoffs.** TFs were included as De Novo Th17 Core TFs if, at FDR = 10%, (1) positive gene targets were enriched for up-regulated Th17 genes (red bars) or (2) negative gene targets were enriched for down-regulated Th17 genes (blue bars). The top-30 most significant “core” Th17 TFs are shown per TRN. Superscripts c and y indicate TF Th17 association from (Ciofani et al. 2012), (Yosef et al., 2013), respectively.

**Supplemental Fig. S13. Effect of model-size cutoff on high-degree TFs.** A TF was included in the “high-degree” TF set, if it was within the top-three highest-degree TFs in one or more of the TRNs. Color scale indicates the percentage of (the 3758) target genes regulated by the TF.

**Supplemental Fig. S14. Top 15 TF modules for ChIP+KO+ATAC TRN.** TFs were clustered into modules based on shared positive target genes between TFs (see **Methods**). The left panel shows enrichment of positive TF target interactions with up-regulated Th17 genes (“Th17” for potential Th17-promoting TFs) or down-regulated Th17 genes (“Non-Th17”, for potential Th17-repressing TFs). The lower panel shows relative TF gene expression over the Th17 and other T Helper cell time points.

**Supplemental Fig. S15. Top 15 TF modules for ATAC-only TRN.** TFs were clustered into modules based on shared positive target genes between TFs (see **Methods**). The left panel shows enrichment of positive TF target interactions with up-regulated Th17 genes (“Th17” for potential Th17-promoting TFs) or down-regulated Th17 genes (“Non-Th17”, for potential Th17-repressing TFs). The lower panel shows relative TF gene expression over the Th17 and other T Helper cell time points.

**Supplemental Fig. S16. Gene-set enrichment of ChIP+KO+ATAC TRN TF modules.** We compiled gene sets from five databases: GO, Pathway Commons, KEGG, MAPP and Signatures from MSigDB. For each TF, we calculated the significance of overlap between the TF’s target genes and gene sets (hypergeometric CDF). For Kegg and Mapp databases, the color bar maximum is 7, and, for the other databases, the maximum is 11. TFs are clustered and color-coded according to the TF-TF modules in **Fig. 5C, S14, S15, S17**. TF-TF module enrichments are consistent among gene sets and TRNs, providing robust predictions for modules in Th17 biology.

**Supplemental Fig. S17. Gene-set enrichment of ATAC-only TRN TF modules.** We compiled gene sets from five databases: GO, Pathway Commons, KEGG, MAPP and Signatures from MSigDB. For each TF, we calculated the significance of overlap between the TF’s target genes and gene sets (hypergeometric CDF). For Kegg and Mapp databases, the color bar maximum is 7, and, for the other databases, the maximum is 11. TFs are clustered and color-coded according to the TF-TF modules in **Fig. 5C, S14-S16**. TF-TF module enrichments are consistent among gene sets and TRNs, providing robust predictions for modules in Th17 biology.

**Supplemental Fig. S18. (A) Centrality analysis of the full Th17 TRN.** TFs are plotted as a function of out degree (fraction of target genes regulated by the TF) and betweenness (fraction of shortest paths (from TFs to genes) containing the TF). **(B) NFKB2 targets are enriched in chronic inflammatory disease genes**. In the left panel, each arrow corresponds to a single TF. Arrow source is TF’s centrality (out degree, betweenness) in the full Th17 TRN **(A)** and arrow points to the TF’s centrality in the chronic inflammatory disease subnetwork (where target genes are limited to the 38 shared the Th17 TRN and GWAS set). NFKB2 (pink arrow) has significant increase in degree centrality (FDR=10%); other TFs (RORC, STAT3, FOXB1) also increase, but not significantly. The right panel features the subnetwork connecting NFKB2 to its target genes in the chronic inflammatory diseases. Node color indicates log_2_(fold-change) in Th17 48h condition relative to other Th timepoints (red = increased, blue = decreased), while red / blue indicate positive / negative regulation. Solid edges have support in the ChIP+KO+ATAC prior, while dotted edges do not. “Chronic inflammatory diseases” is an abbreviation of the trait “Chronic inflammatory diseases (ankylosing spondylitis, Crohn's disease, psoriasis, primary sclerosing cholangitis, ulcerative colitis) (pleiotropy)”. **(C) ETS1 targets are enriched in the phenotype “neutrophil % of granulocytes” genes.** Analysis is displayed as in **(B)**, with the following exception: gene expression in the ETS1 subnetwork corresponds to the Th17 1h timepoint, as *Ets1* expression is highest at early timepoints (Th0 1h, Th17 1h and no media control timepoints).

**Supplemental Fig. S19.** Representative outputs from ATACseqQC, displaying nucleosome-length periodicity in fragment lengths and signal distribution at the TSS for **(A)** naive CD4 T cells (2h), **(B)** Th17 (2h), **(C)** Th17 (16h), and **(D)** Th17 (48h).

**Supplemental Fig. S20. Comparison of CisBP with and without ENCODE-derived motifs.** Priors were derived from the Th17 48h (Th17) or all T Helper (All Th) ATAC-seq samples using CisBP + human ENCODE motifs or CisBP. Two raw p-value cutoffs were tested. mLASSO-StARS was run with TF mRNA or prior-based TFA at bias = .5.

**Supplemental Fig. S21**. **Comparison of peak-gene association rules.** Priors were constructed from the Th17 48h ATAC-seq data using different peak-gene association rules and two raw p-value cutoffs for motifs from CisBP (note that ENCODE motifs were not included in this analysis). mLASSO-StARS was run with TF mRNA or prior-based TFA at bias = .5. (Inf = infinity.)

**Supplemental Fig. S22. The effect of TFA and prior reinforcement on recovery of prior edges in TRN models.** % prior edges in the TRNs are plotted as a function of TRN size (mean TF predictors per gene) for the two Inferelator methods (BBSR-BIC and mLASSO-StARS), the two methods for estimating TF activities (TFA = P^+^X and TF mRNA), and varying levels of prior reinforcement (-, +, ++ correspond to no, moderate, and high prior reinforcement, respectively).

**Supplemental Fig. S23**. **Comparison of steady-state versus time-lagged treatment of time-series data.** The gene expression matrix includes a small fraction of time-series samples (15 out of 254). Using the time-lag parameter tau (30 minutes) from (Ciofani et al. 2012), we compared performance between TRNs built with and without time-lag (mLASSO-StARS with the Th17 ATAC prior, moderate prior reinforcement (bias $=.5$), and either TFA = TF mRNA or prior-based TFA. Precision-recall of the **(A)** KO+ChIP and **(B)** KO G.S.’s did not change between time-lagged and steady-state models.

**Supplemental Fig. S24**. **(A) Per-gene and (B) network-level average instability paths.** Average instabilities were calculated on per-gene and network level over a range of LASSO penalties, $\lambda$. Results are shown for the No Prior TRN using 50 subsamples.

**Supplemental Fig. S25**. **Distribution of nonzero subsamples per TF-gene edge.** Distribution of nonzero subsamples per TF-gene, using per-gene and network average instability cutoffs of .05, .1, and .2. Results are shown for the No Prior TRN using 50 subsamples. The dotted-red line marks the instability cutoff in terms of nonzero subsamples, and the average model size at that cutoff appears as text in the upper right hand.

**Supplemental Fig. S26**. **Comparison of edge-ranking methods**. Edges are ranked according to **(A)** nonzero subsamples or **(B)** nonzero subsamples plus the absolute value of edge partial correlation (**Equation 4**) for network- and gene-level average instabilities at cutoffs .05, .1 and .2. Right and left panels denote full and zoomed-in curves, respectively. Results are shown for the Th17 ATAC prior, bias = .5, and prior-based TFA, using the KO + ChIP G.S.

**Supplemental Fig. S27.** **StARS parameterization: precision-recall of the KO+ChIP G.S.** TRNs were built using the Th17 ATAC prior with prior-based or TF mRNA TFA, no, moderate or strong prior reinforcement, network- and gene-level average instabilities at cutoffs .05, .1 and .2. Edges are ranked according to nonzero subsamples or **Equation 4**.

**Supplemental Fig. S28**. **StARS parameterization: precision-recall of the KO G.S.** TRNs were built using the Th17 ATAC prior with prior-based or TF mRNA TFA, no, moderate or strong prior reinforcement, network- and gene-level average instabilities at cutoffs .05, .1 and .2. Edges are ranked according to nonzero subsamples or **Equation 4**.

**Supplemental Fig. S29**. **StARS parameterization: out-of-sample gene expression prediction.** TRNs were built using the Th17 ATAC prior with prior-based or TF mRNA TFA, no, moderate or strong prior reinforcement, network- and gene-level average instabilities at cutoffs .05, .1 and .2. Out-of-sample gene expression prediction was tested on the leave-out “Early Th17” set (8 samples). **(A)** and **(B)** show overall R^2^_pred_ or the median-gene’s R^2^_pred_ as a function of average model size, using **Equation 4** to rank TF-gene interactions.

**Supplemental Fig. S30**. **Computational speed-up with bStARS lambda bounds.** **(A)** Network instabilities were estimated using 50 subsamples for Th17 ATAC prior with prior-based TFA and moderate reinforcement over a range of lambda penalties containing the lambda corresponding to the target instability cutoff .05 (vertical black line). bStARS upper and lower instability bounds, based on **(B)** two or **(C)** five subsamples shorten the lambda search space (orange arrows, and black lines indicate lower and upper bounds for lambda corresponding to the target instability cutoff .05). Solving the LASSO is very slow for smaller lambda penalties; thus, increasing the lower bound (as in **(C)**) results in significant speed-up (~2-fold), while looser bounds (as in **(B)**) do not.

**Supplemental Fig. S31**. **Effect of individual target genes on prior-based TFA estimation.** The empirical cumulative distribution function (CDF) of Pearson correlation coefficients between TFA estimates from **Equation 2** versus **Equation 6**, using the Th17 ATAC prior. For each of the 2093 target genes with edges in the prior $P$, **Equation 6** is solved for A^-i^. For each gene i, correlations are limited to those TFs k for which $P\left( i,k \right)\neq0$, yielding 10,8850 correlation coefficients.

**Supplemental Fig. S32. Effect of individual target genes on prediction using prior-based TFA.** For the three out-of-sample prediction challenges, gene models were built using Equation 2 (“TFA”) or **Equation 6** (“TFA^-gene i^"), using mLASSO-StARS, Th17 ATAC prior, and moderate prior reinforcement. (A) Overall R^2^_pred_ and median per-gene R^2^_pred_ for all genes or limited to those genes with edges in the prior. (B) The CDFs of per-gene R^2^_pred_ are plotted for average model size of 15 TFs/gene. Thus, in this context, evaluation of out-of-sample gene expression prediction can be accomplished using **Equation 2** (versus the more computationally intensive **Equation 6**).

**Supplemental Fig. S33**. **Rank-combination of ATAC TRNs.** The precision-recall of individual ATAC TRNs (at moderate prior reinforcement “b=50” for prior-based and TF mRNA TFA) are compared to performance average- or maximum-combination of TRNs.

**Supplemental Fig. S34**. **Rank-combination of TRNs from different priors.** The precision-recall of TRNs from individual priors and pairs of priors (at moderate prior reinforcement “b=50” for both prior-based and TF mRNA TFA) are compared, using **(A)** maximum- or **(B)** average-combination of TRNs.

**Supplemental Fig. S35**. **Ranking of TF-TF Clusters.** Heuristic significance estimates versus cluster size for TF-TF modules built from **(A)** positive and **(B)** negative TF-TF edge overlaps. Results are shown for the “final” ChIP+KO+ATAC TRN.

**Supplemental Table Legends**

**Table S1.** **Statistics on priors and gold standards. (A)** and **(B)** were generated using the initial and updated mouse TF lists, respectively (see **Methods**).

**Table S2. Normalized gene expression matrix.** Data were normalized as described in **Methods**, and final units are DESeq2 VSD counts.

**Table S3. Target gene and candidate regulator lists.**

**Table S4. Lists of sample names corresponding to each leave-out set.**

**Table S5. Final KO+ChIP+ATAC and ATAC-only TRNs.**

**Table S6. TF target enrichment in GWAS phenotype genes.**

**Supplemental Codebases**

**infTRN_lassoStARS.** Workflows and codebase for inference of transcriptional regulatory network inference from gene expression and prior information (e.g., ATAC-seq). The codebase is hosted at https://github.com/emiraldi/infTRN_lassoStARS.

**jp_gene_viz.** iPython Jupyter notebook codebase for interactive visualization of transcriptional regulatory networks and genomics data. The codebase is available from https://github.com/simonsfoundation/jp_gene_viz.

**Th17_TRN_Networks.** The iPython Jupyter notebooks integrating the 36 TRNs, including final ATAC+KO+ChIP as well as ATAC-only Th17 TRNs, for interactive visualization. Notebooks and networks are available from https://github.com/simonsfoundation/Th17_TRN_Networks.

**Supplemental References**

Bonneau R, Reiss DJ, Shannon P, Facciotti M, Hood L, Baliga NS, Thorsson V. 2006. The Inferelator: an algorithm for learning parsimonious regulatory networks from systems-biology data sets de novo. *Genome Biol* **7**: 1.

Castro DM, Veaux N de, Miraldi ER, Bonneau R. 2018. Multi-study inference of regulatory networks for more accurate models of gene regulation. *bioRxiv* 279224.

Ciofani M, Madar A, Galan C, Sellars M, Mace K, Pauli F, Agarwal A, Huang W, Parkurst CN, Muratet M, et al. 2012. A validated regulatory network for Th17 cell specification. *Cell* **151**: 289‑303.

Derrien T, Estellé J, Sola SM, Knowles DG, Raineri E, Guigó R, Ribeca P. 2012. Fast computation and applications of genome mappability. *PLoS One* **7**: e30377.

Dobin A, Davis CA, Schlesinger F, Drenkow J, Zaleski C, Jha S, Batut P, Chaisson M, Gingeras TR. 2013. STAR: Ultrafast universal RNA-seq aligner. *Bioinformatics* **29**: 15‑21.

Faith JJ, Hayete B, Thaden JT, Mogno I, Wierzbowski J, Cottarel G, Kasif S, Collins JJ, Gardner TS. 2007. Large-scale mapping and validation of Escherichia coli transcriptional regulation from a compendium of expression profiles. *PLoS Biol* **5**: e8.

Han H, Shim H, Shin D, Shim JE, Ko Y, Shin J, Kim H, Cho A, Kim E, Lee T, et al. 2015. TRRUST: a reference database of human transcriptional regulatory interactions. *Sci Rep* **5**: 11432.

Kittler J, Hater M, Duin RPW. 1996. Combining classifiers. *Proc - Int Conf Pattern Recognit* **2**: 897‑901.

Kurtz ZD, Müller CL, Miraldi ER, Littman DR, Blaser MJ, Bonneau RA. 2015. Sparse and Compositionally Robust Inference of Microbial Ecological Networks. *PLoS Comput Biol* **11**: e1004226.

Love MI, Huber W, Anders S. 2014. Moderated estimation of fold change and dispersion for RNA-seq data with DESeq2. *Genome Biol* **15**: 550.

Marbach D, Costello JC, Küffner R, Vega NM, Prill RJ, Camacho DM, Allison KR, Aderhold A, Bonneau R, Chen Y. 2012. Wisdom of crowds for robust gene network inference. *Nat Methods* **9**: 796.

Müller CL, Bonneau R, Kurtz Z. 2016. Generalized Stability Approach for Regularized Graphical Models. *arXiv* 1605.07072.

Parisi F, Strino F, Nadler B, Kluger Y. 2014. Ranking and combining multiple predictors without labeled data. *Proc Natl Acad Sci U S A* **111**: 1253‑8.

Stergachis AB, Neph S, Sandstrom R, Haugen E, Reynolds AP, Zhang M, Byron R, Canfield T, Stelhing-Sun S, Lee K, et al. 2014. Conservation of trans-acting circuitry during mammalian regulatory evolution. *Nature* **515**: 365‑370.

Whitlock MC. 2005. Combining probability from independent tests: the weighted Z-method is superior to Fisher’s approach. *J Evol Biol* **18**: 1368‑73.
