# Supplementary material for: Leveraging chromatin accessibility for transcriptional regulatory network inference in T Helper 17 Cells
Source: Genome Res. 2019 Mar;29(3):449–63. doi: 10.1101/gr.238253.118 (PMC6396413; doi:10.1101/gr.238253.118)
Supplement: Supplemental Material [file supp_gr.238253.118_Supplemental_Table_S1.pdf]

Table S1

A

	Full Prior (All Genes + TFs)			Prior Limited to Model TFs & Genes			Overlap ChIP G.S.		Overlap KO G.S.		Overlap KO+ChIP G.S.	
**Prior**	**# Edges**	**# TFs**	**# Genes**	**# Edges**	**# TFs**	**# Genes**	**#TFs**	**% Edges**	**#TFs**	**% Edges**	**#TFs**	**% Edges**
ATAC (Th17)	1,120,541	842	17,141	112,866	357	2,804	9	43%	23	20%	9	42%
ATAC (aTh)	1,906,421	831	20,721	191,508	351	3,201	9	54%	23	28%	9	51%
TRRUST	7,176	582	2,174	1,529	215	512	8	1%	20	0%	8	1%
ENCODE DHS	1,552,681	546	17,471	197,156	245	3,362	7	29%	17	24%	7	33%
ChIP	70,652	9	14,945	13,925	9	2,869	9	87%	9	34%	9	89%
ChIP/A(Th17)	1,163,624	842	19,549	121,350	357	3,217	9	87%	23	40%	9	89%
ChIP/A(Th17) + KO	1,178,955	844	19,686	126,661	359	3,258	9	90%	25	100%	9	100%
ChIP/A(Th17) + KO + A(aTh)	2,070,038	844	21,890	212,534	359	3,353	9	94%	25	100%	9	100%
ChIP	22,334	9	7,107	6,174	9	1,696	9	100%	9	20%	9	75%
KO	22,693	26	7,088	8,875	25	1,766	9	29%	25	100%	9	100%
KO + ChIP	4,249	9	2,223	2,375	9	996	9	29%	9	27%	9	100%

B

	Full Prior (All Genes + TFs)			Prior Limited to Model TFs & Genes			Overlap ChIP G.S.		Overlap KO G.S.		Overlap KO+ChIP G.S.	
**Prior**	**# Edges**	**# TFs**	**# Genes**	**# Edges**	**# TFs**	**# Genes**	**#TFs**	**% Edges**	**#TFs**	**% Edges**	**#TFs**	**% Edges**
ATAC (Th17)	1,083,589	811	17,138	109,513	346	2,803	9	43%	23	21%	9	42%
ATAC (aTh)	1,843,825	801	20,720	185,837	340	3,201	9	54%	23	30%	9	51%
TRRUST	6,686	488	2,123	1,448	187	502	8	1%	19	1%	8	1%
ENCODE DHS	1,525,958	536	17,472	193,365	238	3,362	7	29%	17	26%	7	33%
ChIP	70,652	9	14,945	13,925	9	2,869	9	87%	9	37%	9	89%
ChIP/A(Th17)	1,126,672	811	19,548	117,997	346	3,217	9	87%	23	43%	9	89%
ChIP/A(Th17) + KO	1,140,685	811	19,685	122,760	346	3,258	9	90%	23	100%	9	100%
ChIP/A(Th17) + KO + A(aTh)	2,004,885	811	21,890	206,103	346	3,353	9	94%	23	100%	9	100%
ChIP	22,334	9	7,107	6,174	9	1,696	9	100%	9	22%	9	75%
KO	21,375	24	7,015	8,327	23	1,759	9	29%	23	100%	9	100%
KO + ChIP	4,249	9	2,223	2,375	9	996	9	29%	9	29%	9	100%

Table S1. Statistics on Priors and Gold Standards. (A) and **(B)** were generated using the initial and updated mouse TF lists, respectively (see **Methods**).
