# Supplementary material for: Leveraging chromatin accessibility for transcriptional regulatory network inference in T Helper 17 Cells
Source: Genome Res. 2019 Mar;29(3):449–63. doi: 10.1101/gr.238253.118 (PMC6396413; doi:10.1101/gr.238253.118)
Supplement: Supplemental Material [file supp_gr.238253.118_Supplemental_infTRN_lassoStARS.zip › infTRN_lassoStARS-master/glmnet/glmnetMex.pdf]

include "fintrf.h" C glmnetMex.F C C Lasso and elastic-net regularized generalized linear models

C [a0,ca,ia,nin,rsq,alm,nlp,jerr] = ... C glmnetMex(parm,x,y,jd,vp,ne,nx,nlam,flmin,ulam,thr,isd,w,ka) C [a0,ca,ia,nin,dev,alm,nlp,jerr] = ... C glmnetMex(parm,x,y,jd,vp,ne,nx,nlam,flmin,ulam,thr,isd,nc,maxit,kopt) C C Extremely efficient procedures for fitting the entire lasso or C elastic-net regularization path for linear regression, logistic and C multinomial regression models. The algorithm uses cyclical coordinate C descent in a pathwise as described in the paper on the maintainer's C website. C C NOTES: This is a MEX-file wrapper of GLMnet.f for MATLAB. Should be called C only by glmnet.m. For details about input and output arguments, see C GLMnet.f. C C LICENSE: GPL-2 C C DATE: 13 Jul 2009 C C AUTHORS: C Algorithm designed by Jerome Friedman, Trevor Hastie and Rob Tibshirani C Fortran code written by Jerome Friedman C MATLAB wrapper written and maintained by Hui Jiang, jiangh@stanford.edu C Department of Statistics, Stanford University, Stanford, California, USA. C C REFERENCES: C Friedman, J., Hastie, T. and Tibshirani, R. (2009) C Regularization Paths for Generalized Linear Models via Coordinate Descent. C Journal of Statistical Software, 33(1), 2010 C C EXAMPLE: C parm = 1.0; C x = [1 1; 2 2; 3 3]; C y = [1 3 2]'; C jd = 0; C vp = [1 1]'; C ne = 3; C nx = 2; C nlam = 100; C flmin = 0.0001; C ulam = 0; C thr = 1.0e-4; C isd = 1; C w = [1 1 1]'; C ka = 2; C [a0,ca,ia,nin,rsq,alm,nlp,jerr] = glmnetMex(parm,x,y,jd,vp,ne,nx,nlam,flmin,ulam,thr,isd,w,ka) C C DEVELOPMENT: 13 Jul 2009: Original version of glmnetMex.f written. C C———————————————————————

subroutine mexFunction(nlhs, plhs, nrhs, prhs) C——————————————————————— C (pointer) Replace integer by integer*8 on the DEC Alpha C 64-bit platform

mwpointer plhs(*), prhs(*) mwpointer mxCreateDoubleMatrix, mxGetPr mwpointer mxGetIr, mxGetJc integer nlhs, nrhs mwsize mxGetM, mxGetN, mxGetNzmax integer mxIsNumeric integer mxIsSparse

C———————————————————————

C Input real parm,flmin,thr,intr integer ka,no,ni,nc,ne,nx,nlam,isd,maxit,kopt,isparse,nzmax integer, dimension (2) :: dimx real, dimension (:), allocatable :: x,y,w,vp,ulam,cl,sr integer, dimension (:), allocatable :: ix,jx,jd,irs,jcs

mwpointer pr

C Output integer lmu,nlp,jerr real, dimension (:), allocatable :: a0,ca,alm,dev,rsq integer, dimension (:), allocatable :: ia,nin

C Temporary mwpointer temp$_prmwsizetemp_m, temp_n, temp_nzmaxintegertask, i$

C For internal parameters real fdev, devmax, eps, big, pmin, prec integer mnlam, exmx, mxit

C Check for proper number of arguments.

if(nrhs .eq. 18 .and. nlhs .eq. 8) then task = 1; elseif(nrhs .eq. 15 .and. nlhs .eq. 8) then task = 2; elseif(nrhs .eq. 0) then task = 3; elseif(nrhs .eq. 9) then task = 4; else call mexErrMsgTxt('Incorrect number of arguments.') endif

C Get input

if (task .eq. 3) then call get$_int_parms(fdev, eps, big, mnlam, devmax, pmin, exmx)callget_bnorm(prec, mxit)$

plhs(1) = mxCreateDoubleMatrix(1,1,0) temp$_pr = mxGetPr(plhs(1))callputreal(fdev, temp_pr, 1)$

plhs(2) = mxCreateDoubleMatrix(1,1,0) temp$_pr = mxGetPr(plhs(2))callputreal(devmax, temp_pr, 1)$

plhs(3) = mxCreateDoubleMatrix(1,1,0) temp$_pr = mxGetPr(plhs(3))callputreal(eps, temp_pr, 1)$

plhs(4) = mxCreateDoubleMatrix(1,1,0) temp$_pr = mxGetPr(plhs(4))callputreal(big, temp_pr, 1)$

plhs(5) = mxCreateDoubleMatrix(1,1,0) temp$_pr = mxGetPr(plhs(5))callputinteger(mnlam, temp_pr, 1)$

plhs(6) = mxCreateDoubleMatrix(1,1,0) temp$_pr = mxGetPr(plhs(6))callputreal(pmin, temp_pr, 1)$

plhs(7) = mxCreateDoubleMatrix(1,1,0) temp$_pr = mxGetPr(plhs(7))callputinteger(exmx, temp_pr, 1)$

plhs(8) = mxCreateDoubleMatrix(1,1,0) temp$_pr = mxGetPr(plhs(8))callputreal(prec, temp_pr, 1)$

plhs(9) = mxCreateDoubleMatrix(1,1,0) temp$_pr = mxGetPr(plhs(9))callputinteger(mxit, temp_pr, 1)elseif(task.eq.4)th$ $mxGetPr(prhs(1))callgetreal(temp_pr, fdev, 1)$

temp$_pr = mxGetPr(prhs(2))callgetreal(temp_pr, devmax, 1)$

temp$_pr = mxGetPr(prhs(3))callgetreal(temp_pr, eps, 1)$

temp$_pr = mxGetPr(prhs(4))callgetreal(temp_pr, big, 1)$

temp$_pr = mxGetPr(prhs(5))callgetinteger(temp_pr, mnlam, 1)$

temp$_pr = mxGetPr(prhs(6))callgetreal(temp_pr, pmin, 1)$

temp$_pr = mxGetPr(prhs(7))callgetinteger(temp_pr, exmx, 1)$

temp$_pr = mxGetPr(prhs(8))callgetreal(temp_pr, prec, 1)$

temp$_{p}r = mxGetPr(prhs(9)) call getinteger(temp_{p}r, mxit, 1)$

call chg$_{f}ract_{d}ev(fdev) call chg_{d}ev_{m}ax(devmax) call chg_{m}in_{f}lmin(eps) call chg_{b}ig(big) call chg_{m}in_{l}ambdas(mnlam) call chg$

else

temp$_{p}r = mxGetPr(prhs(1)) call getreal(temp_{p}r, parm, 1)$

c

isparse = mxIsSparse(prhs(2)) if (1 .eq. 1) then c

temp$_{n}zmax = mxGetNzmax(prhs(2))c$

$$nzmax = temp_{n}zmaxc$$

$temp_{p}r = mxGetPr(prhs(18))c$

$$call getinteger(temp_{p}r, dimx, 2)c$$

$no = dimx(1)c$

$$ni = dimx(2)c$$

$allocate(sr(1 : nzmax))c$

$$allocate(irs(1 : nzmax))c$$

$allocate(jcs(1 : (ni + 1)))c$

$$temp_{p}r = mxGetPr(prhs(2))c$$

$call getreal(temp_{p}r, sr, nzmax)c$

$$temp_{p}r = mxGetIr(prhs(2))c$$

$call getinteger(temp_{p}r, irs, nzmax)c$

$$doi = 1, nzmaxc$$

$irs(i) = irs(i) + 1c$

$$enddoc$$

$temp_{p}r = mxGetJc(prhs(2))c$

$$call getinteger(temp_{p}r, jcs, ni + 1)c$$

$doi = 1, (ni + 1)c$

$$jcs(i) = jcs(i) + 1c$$

enddo

allocate(sr(1:5)) allocate(irs(1:5)) allocate(jcs(1:5))

no = 4; ni = 4

sr(1) = 1; sr(2) = 2; sr(3) = 10; sr(4) = 3; sr(5) = 4; irs(1) = 1; irs(2) = 2; irs(3) = 4; irs(4) = 3; irs(5) = 4; jcs(1) = 1; jcs(2) = 2; jcs(3) = 4; jcs(4) = 5; jcs(5) = 6;

else temp$_{p}r = mxGetPr(prhs(2)) temp_{m} = mxGetM(prhs(2)) no = temp_{m} temp_{n} = mxGetN(prhs(2)) ni = temp_{n} allocate(x(1 : no * ni)) call getreal(temp_{p}r, x, no * ni)$

endif

temp$_{p}r = mxGetPr(prhs(4)) temp_{m} = mxGetM(prhs(4)) temp_{n} = mxGetN(prhs(4)) allocate(jd(temp_{m} * temp_{n})) call getinteger(temp_{p}r, jd, temp_{m} * temp_{n})$

temp$_{p}r = mxGetPr(prhs(5)) allocate(vp(1 : ni)) call getreal(temp_{p}r, vp, ni)$

temp$_{p}r = mxGetPr(prhs(6)) call getinteger(temp_{p}r, ne, 1)$

temp$_{p}r = mxGetPr(prhs(7)) call getinteger(temp_{p}r, nx, 1)$

temp$_{p}r = mxGetPr(prhs(8)) call getinteger(temp_{p}r, nlam, 1)$

temp$_{p}r = mxGetPr(prhs(9)) call getreal(temp_{p}r, flmin, 1)$

temp$_{p}r = mxGetPr(prhs(10)) temp_{m} = mxGetM(prhs(10)) temp_{n} = mxGetN(prhs(10)) allocate(ulam(1 : temp_{m} * temp_{n})) call getreal(temp_{p}r, ulam, temp_{m} * temp_{n})$

temp$_pr = mxGetPr(prhs(11))callgetreal(temp_pr, thr, 1)$

temp$_pr = mxGetPr(prhs(12))callgetinteger(temp_pr, isd, 1)$

if (task .eq. 1) then temp$_pr = mxGetPr(prhs(3))allocate(y(1 : no))callgetreal(temp_pr, y, no)$

temp$_pr = mxGetPr(prhs(13))allocate(w(1 : no))callgetreal(temp_pr, w, no)$

temp$_pr = mxGetPr(prhs(14))callgetinteger(temp_pr, ka, 1)$

temp$_pr = mxGetPr(prhs(15))allocate(cl(1 : 2 * ni))callgetreal(temp_pr, cl, 2 * ni)$

temp$_pr = mxGetPr(prhs(16))callgetinteger(temp_pr, intr, 1)$

temp$_pr = mxGetPr(prhs(17))callgetinteger(temp_pr, maxit, 1)$

elseif (task .eq. 2) then temp$_pr = mxGetPr(prhs(13))callgetinteger(temp_pr, nc, 1)$

temp$_pr = mxGetPr(prhs(14))callgetinteger(temp_pr, maxit, 1)$

temp$_pr = mxGetPr(prhs(15))callgetinteger(temp_pr, kopt, 1)$

temp$_pr = mxGetPr(prhs(3))allocate(y(1 : no*(max(2, nc))))callgetreal(temp_pr, y, no*(max(2, nc)))endif$

C Allocate memory for output allocate(ia(1:nx)) call zerointeger(ia,nx) allocate(nin(1:nlam)) call zerointeger(nin,nlam) allocate(alm(1:nlam)) call zeroreal(alm,nlam) if (task .eq. 1) then allocate(a0(1:nlam)) call zeroreal(a0,nlam)

allocate(ca(1:nx*nlam)) call zeroreal(ca,nx*nlam)

allocate(rsq(1:nlam)) call zeroreal(rsq,nlam) elseif (task .eq. 2) then allocate(a0(1:nc*nlam)) call zeroreal(a0,nc*nlam)

allocate(ca(1:nx*nc*nlam)) call zeroreal(ca,nx*nc*nlam)

allocate(dev(1:nlam)) call zeroreal(dev,nlam)

endif

C Call glmnet lmu = 0 nlp = 0 jerr = 1 if (task .eq. 1) then if (isparse .ne. 1) then call elnet(ka,parm,no,ni,x,y,w,jd,vp,cl,ne,[illegible] $ulam, thr, isd, intr, maxit, lmu, a0, ca, ia, nin, rsq, alm, nlp$,jerr) else call spelnet(ka,parm,no,ni,sr,jcs,irs,y,w,jd,vp,cl,ne,nx, $nlam, flmin, ulam, thr, isd, intr, maxit, lmu, a0, ca, ia, nin,$ rsq,alm,nlp,jerr) endif elseif (task .eq. 2) then call lognet(parm,no,ni,nc,x,y,jd,vp,ne,nx,nlam,flmin,ulam,t $hr, isd, maxit, kopt, lmu, a0, ca, ia, nin, dev, alm, nlp, jerr)endif$

C Prepare output plhs(3) = mxCreateDoubleMatrix(nx,1,0) temp$_pr = mxGetPr(plhs(3))callputinteger(ia, temp_pr, nx)$

plhs(4) = mxCreateDoubleMatrix(lmu,1,0) temp$_pr = mxGetPr(plhs(4))callputinteger(nin, temp_pr, lmu)$

plhs(6) = mxCreateDoubleMatrix(lmu,1,0) temp$_pr = mxGetPr(plhs(6))callputreal(alm, temp_pr, lmu)$

plhs(7) = mxCreateDoubleMatrix(1,1,0) temp$_pr = mxGetPr(plhs(7))callputinteger(nlp, temp_pr, 1)$

plhs(8) = mxCreateDoubleMatrix(1,1,0) temp$_pr = mxGetPr(plhs(8))callputinteger(jerr, temp_pr, 1)$

if (task .eq. 1) then plhs(1) = mxCreateDoubleMatrix(lmu,1,0) temp$_pr = mxGetPr(plhs(1))callputreal(a0, temp_pr, lmu)$

plhs(2) = mxCreateDoubleMatrix(nx,lmu,0) temp$_pr = mxGetPr(plhs(2))callputreal(ca, temp_pr, nx * lmu)$

plhs(5) = mxCreateDoubleMatrix(lmu,1,0) temp$_pr = mxGetPr(plhs(5))callputreal(rsq, temp_pr, lmu)elseif(task.eq.2)t$[illegible] $mxCreateDoubleMatrix(nc, lmu, 0)temp_pr = mxGetPr(plhs(1))callputreal(a0, temp_pr, nc * lmu)$

plhs(2) = mxCreateDoubleMatrix(nx*nc,lmu,0) temp$_pr = mxGetPr(plhs(2))callputreal(ca, temp_pr, nx* nc * lmu)$

plhs(5) = mxCreateDoubleMatrix(lmu,1,0) temp$_pr = mxGetPr(plhs(5))callputreal(dev, temp_pr, lmu)endif$

C Deallocate memory deallocate(x) deallocate(y) deallocate(jd) deallocate(vp) deallocate(ulam) deallocate(a0) deallocate(ca) deallocate(ia) deallocate(nin) deallocate(alm)

C For logistic elastic net if (task .eq. 1) then deallocate(w) deallocate(rsq) deallocate(cl) if (isparse .eq. 1) then deallocate(sr) deallocate(irs) deallocate(jcs) endif elseif (task .eq. 2) then deallocate(dev) endif endif

return end

C End of subroutine mexFunction

subroutine real8toreal(x, y, size) integer size real*8 x(size) real y(size) do 10 i=1,size y(i)= x(i) 10 continue return end

subroutine realtoreal8(x, y, size) integer size real x(size) real*8 y(size) do 20 i=1,size y(i)= x(i) 20 continue return end

subroutine real8tointeger(x, y, size) integer size real*8 x(size) integer y(size) do 30 i=1,size y(i)= x(i) 30 continue return end

subroutine integertoreal8(x, y, size) integer size integer x(size) real*8 y(size) do 40 i=1,size y(i)= x(i) 40 continue return end

subroutine getreal(pr,x,size) mwpointer pr integer size real x(size) real*8, dimension (:), allocatable :: temp allocate(temp(1:size)) call mxCopyPtrToReal8(pr,temp,size) call real8toreal(temp,x,size) deallocate(temp) return end

subroutine getinteger(pr,x,size) mwpointer pr integer size integer x(size) real*8, dimension (:), allocatable :: temp allocate(temp(1:size)) call mxCopyPtrToReal8(pr,temp,size) call real8tointeger(temp,x,size) deallocate(temp) return end

subroutine putreal(x,pr,size) mwpointer pr integer size real x(size) real*8, dimension (:), allocatable :: temp allocate(temp(1:size)) call realtoreal8(x,temp,size) call mxCopyReal8ToPtr(temp,pr,size) deallocate(temp) return end

subroutine putinteger(x,pr,size) mwpointer pr integer size integer x(size) real*8, dimension (:), allocatable :: temp allocate(temp(1:size)) call integertoreal8(x,temp,size) call mxCopyReal8ToPtr(temp,pr,size) deallocate(temp) return end

subroutine zeroreal(x,size) integer size real x(size) do 90 i=1,size x(i) = 0 90 continue return end

subroutine zerointeger(x,size) integer size integer x(size) do 100 i=1,size x(i) = 0 100 continue return end
